# Residues Ser83, Arg85, Tyr88, Asn124, and Lys192 of C-Terminal Lipid-Associated Membrane Hemagglutinin Affect *Mycoplasma synoviae* Agglutination of Erythrocytes

**DOI:** 10.3390/microorganisms14092031

**Published:** 2026-09-12

**Authors:** Duoduo Si, Shijun Bao, Lei Guo, Xiuhong Chen, Fengqin Wen, Xiaoxiao He, Qing Wang, Yuan Shi, Shenghu He, Jidong Li

**Affiliations:** 1 Animal Infections Disease Laboratory, College of Veterinary Medicine, Gansu Agricultural University, Lanzhou 730070, China; s956173936@163.com (D.S.); bsjdy@126.com (S.B.); fengqinw@163.com (F.W.); 15117161449@163.com (X.H.); 17393189362@163.com (Q.W.); syuanhi@163.com (Y.S.); 2 Clinical Veterinary Laboratory, College of Animal Science and Technology, Ningxia University, Yinchuan 750021, China; guoleinxyc@163.com; 3 Ningxia Xiaoming Agriculture & Animal Husbandry Co., Ltd., Yinchuan 750011, China; 4 Ningxia Hui Autonomous Region Veterinary Drug Feed Supervision Institute, Yinchuan 750011, China; chenxiuhong2540@163.com

**Keywords:** *Mycoplasma synoviae*, lipid-associated membrane hemagglutinin, key amino acid residues, hemagglutination activity, molecular dynamics simulation

## Abstract

*Mycoplasma synoviae* (*M. synoviae*) is an avian pathogen responsible for respiratory disease and synovitis. The VlhA (variable lipoprotein hemagglutinin) family of surface adhesins plays a critical role in host cell attachment, yet the specific residues and structural determinants governing this interaction remain incompletely understood. In this study, we selected the C-terminal lipid-associated membrane hemagglutinin (LAM HA) domain within the VlhA family as the bait protein, based on its conserved C-terminal region. Yeast two-hybrid screening identified 18 LAM HA-interacting host proteins, and molecular docking pinpointed five residues (S83, R85, Y88, N124, K192) as the most frequent interaction hotspots. To assess their collective functional relevance, we constructed a combined deletion mutant lacking these five candidate residues. At the optimal pH of 6.0–6.5, wild-type LAM HA showed the highest titer (1:2), significantly exceeding that at pH 7.0–7.5 (no activity) and pH 5.0–5.5 (1:1) (*p* < 0.05), whereas the deletion mutant displayed a reduced titer (from 2 to 1; *p* = 0.05). Secondary structure analysis at the same pH revealed decreased α-helix content (from 7.90% to 7.57%), along with increased β-sheet (from 38.92% to 38.83%) and random coil (from 10.83% to 10.44%). Molecular dynamics simulations revealed that the deletion mutant exhibited elevated RMSF (from 2.83 ± 1.88 Å to 4.18 ± 2.65 Å) and Rg (from 40.48 ± 1.31 Å to 54.16 ± 4.47 Å) at pH 6.0–6.5, while RMSD showed a slight decrease (11.17 ± 1.33 Å vs. 12.14 ± 0.21 Å), collectively indicating compromised structural stability of the mutant. Collectively, these findings suggest that the LAM HA domain contributes to hemagglutination activity through pH-sensitive conformational stability and specific residue clusters—a property that may be relevant to *M. synoviae* colonization in the acidic microenvironments of the respiratory tract and synovial fluid during infection.

## 1. Introduction

*Mycoplasma synoviae* is an important avian pathogen that causes arthritis and air sac inflammation in chickens and turkeys, resulting in considerable economic losses to the poultry industry worldwide. Due to the lack of a cell wall, several mycoplasma membrane components can directly interact with host cells. Adhesion is the initial step in pathogenic infection and colonization, and cell adhesion-related proteins may therefore play a critical role in pathogenesis [1].

Hemagglutinin has been extensively studied as a key adhesion protein in viral pathogenesis [2,3]. The globular head of influenza virus hemagglutinin adopts a β-sandwich structure formed by antiparallel β-sheets, which contains the receptor-binding site [4]. Different hemagglutinin subtypes exhibit marked variability in receptor binding specificity, which directly affects viral pathogenicity and transmissibility. For example, specific amino acid mutations in H5N1 hemagglutinin can switch its receptor preference from avian to human receptors, representing a critical event for cross-species transmission [5,6]. The hemagglutinin cleavage site also serves as a critical determinant of viral virulence; alterations in its amino acid sequence—such as those observed in the H5N2 subtype—can markedly enhance pathogenicity and transmission [6,7]. In *M. synoviae*, hemagglutinin is an abundant surface-exposed lipoprotein generated via post-translational cleavage of the *vlhA* gene [8]. The VlhA family comprises two major antigenic variants: MSPA (Major Surface Protein A) and MSPB (Major Surface Protein B), which share a conserved N-terminal region encoded by the 5′ end of the *vlhA* gene. MSPA possesses hemagglutinin activity and primarily mediates mycoplasma binding to erythrocytes, representing the first characterized adhesin of this family [9]. In contrast, MSPB contains a proline-rich repeat at its amino terminus that exhibits high strain-to-strain length polymorphism and has been shown to induce nitric oxide secretion in chicken macrophages, as well as IL-6 and IL-1β in chicken monocytes, suggesting a role in modulating host immune responses [10,11]. Recombination between the 5′ *vlhA* and pseudogenes in the genome occurs throughout the *vlhA* locus except for the conserved 5′ region, driving antigenic variation within the C-terminal two-thirds of both MSPB and MSPA. This recombination-based diversity consequently alters the domains involved in erythrocyte binding, contributing to immune evasion and functional variability of the VlhA adhesins [12]. A conserved motif, P-X-(BCAA)-X-F-X-(BCAA)-X-A-K-X-G, has been identified as a sialic acid-binding motif that may mediate hemagglutinin-dependent attachment to host cells. The C-terminal region of MSPA elicits the highest titers of antibodies capable of inhibiting hemagglutination, hemadsorption, and metabolism, suggesting its key role in functional activity [13,14]. Notably, short-chain VlhA variants, which carry a truncated MSPA region, exhibit reduced or altered hemagglutination ability, whereas long-chain forms retaining the complete C-terminal domain are more likely to mediate effective adhesion [14]. The surface of *M. synoviae* is enriched in lipid-associated membrane proteins (LAMP), which play critical roles in adhesion and invasion. The *vlhA*-encoded products, MSPA and MSPB, have been characterized as major membrane proteins of *M. synoviae* [9]. In our recent study, MSPA was identified as a member of the LAMP family [15]; its C-terminal fragment, designated as LAM HA in this study, exhibited differential expression profiles during host cell infection. Among these hemagglutinins, some represent the major adhesins responsible for host cell attachment and erythrocyte agglutination [9,12]. However, the specific residues and structural determinants underlying this activity remain poorly understood.

*M. synoviae* colonizes the respiratory tract and synovial fluid, where inflammation can lower local pH [16]. This acidic environment may influence the conformational stability and receptor-binding activity of the hemagglutinin adhesin, a phenomenon also observed for hemagglutinins in other mycoplasma species [11] and for pH-regulated adhesins in other bacterial pathogens. Mechanistically, pH modulates protein conformation and activity by altering the protonation states of ionizable residues [17], and such pH-induced conformational changes can affect secondary structure elements essential for function [18]. In particular, β-sheets often serve as the structural scaffold for carbohydrate recognition in lectin-like proteins, providing a platform for target binding [19]. To bridge these static structural features with biological function, we employed molecular dynamics (MD) simulations as a complement to traditional experiments, enabling the capture of transient and cryptic conformational states [20,21].

In this study, we characterized the structural features of three LAM HA domains and screened for host proteins interacting with LAM HA. Through identification of these interactors, we sought to elucidate the role of five candidate residues—designated as potential key amino acids (KeyAA)—in erythrocyte agglutination and to investigate the pH dependence of this function. The secondary structure compositions of LAM HA and the combined deletion mutant (ΔKeyAA LAM HA) were analyzed by Fourier transform infrared (FTIR) spectroscopy under different pH conditions, and the conformational stability of each protein under varying pH conditions was assessed by molecular dynamics (MD) simulations. Collectively, in-depth characterization of these functional sites will provide a critical theoretical foundation for monitoring *M. synoviae* infection and for the development of vaccines and targeted therapeutics.

## 2. Materials and Methods

### 2.1. Bacterial Strains and Plasmids

The *M. synoviae* strain used in this study is WVU1853T (ATCC 25204), which was originally isolated from the synovial fluid of a chicken in West Virginia, USA. The gene encoding the bait protein (locus tag VY93_RS01465) is annotated as a “GA module-containing protein” in the NCBI database; however, sequence analysis identifies it as a member of the VlhA family, with the N-terminus corresponding to MSPB and the C-terminus corresponding to MSPA. The C-terminal region, which corresponds to the lipid-associated membrane hemagglutinin, is designated as LAM HA in this study, and was used as the bait for yeast two-hybrid screening. The yeast two-hybrid assay was performed using Y187 and Y2HGold yeast strains and pGADT7/pGBKT7 vectors maintained in our laboratory (Clinical Veterinary Laboratory, Ningxia University, Yinchuan, China), all of which are standard Matchmaker™ system materials (Takara Bio Inc., Kusatsu, Shiga, Japan). For prokaryotic expression, the candidate gene was subcloned into pGEX-4T-1, which is also preserved in the Clinical Veterinary Laboratory of Ningxia University. The gene synthesis service is credited to Sangon Biotech (Shanghai, China).

### 2.2. Hemagglutinin Family of M. synoviae Domain Analysis

According to the hemagglutinin family-related information integrated in the genome re-annotation [22] and lipid-related membrane protein identification experiments [15], to identify conserved amino acid residues potentially involved in haemagglutination, the VlhA protein sequences of 14 *M. synoviae* strains were retrieved from the NCBI Genome database (https://www.ncbi.nlm.nih.gov/datasets/genome/; accessed on 29 September 2024). These sequences correspond to the hemagglutinin encoded by the *vlhA* gene, which belongs to a large multigene family and undergoes antigenic variation through recombination with pseudogenes. Their conserved domains were analyzed with Multiple EM for Motif Elicitation (MEME) (https://meme-suite.org/meme/ (accessed on 29 September 2024)).

### 2.3. Bacterial Two-Hybrid Library Construction and Screening

DF-1 (chicken embryo fibroblast) and HD11 (chicken macrophage-like) cell lines were each infected with *M. synoviae* at a multiplicity of infection (MOI) of 100. At 0 h, 4 h, 8 h, 12 h, and 24 h post-infection, the culture medium was removed, and the cells were washed three times with PBS. Total RNA was extracted from each sample using TRIzol reagent, and RNA purity was assessed by measuring the OD260/280 ratio. To obtain comprehensive coverage for library construction, RNA samples from the two cell lines were pooled at a 1:1 ratio (*v*/*v*), reverse transcribed into cDNA, and used to construct a yeast two-hybrid library according to the Gateway method by CloneMiner II cDNA Library Construction Kit (Invitrogen, Carlsbad, CA, USA) [23].

For yeast two-hybrid screening, the LAM HA gene with BamH I and Xho I restriction enzyme sites was amplified and cloned into the bait plasmid pGBKT7 to obtain pGBKT7-LAM HA. The pGBKT7-LAM HA and pGADT7 empty vector were co-transformed into Saccharomyces cerevisiae Y2HGold strain for self-activation detection. The bait plasmid pGBKT7-LAM HA was used to screen the constructed chicken cell cDNA library for candidate interacting proteins. Positive clones were isolated on SD/-Trp/-Leu/-His/-Ade selective medium containing 200 ng/mL Aureobasidin A (AbA) and 40 μg/mL X-α-Gal. After three rounds of screening, the recovered positive clones were enriched by cultivation in SD/-Trp/-Leu liquid medium for 3 days. Plasmids were extracted using a yeast plasmid extraction kit (TIANGEN, Beijing, China). The cDNA inserts of positive clones were amplified by PCR using vector-specific primers targeting the GAL4 AD region of pGADT7 (forward: 5′-GATGATGAAGATACCCCACCAAACCC-3′; reverse: 5′-GAGGCCGAGGCGGCCGACATG-3′). The amplified fragments were sequenced and aligned to the chicken genome. For point-to-point verification, remaining positive clones with verified sequences were co-transformed into Y2HGold competent cells SD/-Trp/-Leu/-His/-Ade/X-α-Gal/AbA. After confirmation, the corresponding prey plasmids were subjected to sequencing analysis. Gene sequencing services were provided by Sangon Biotech (Shanghai, China).

### 2.4. Molecular Docking of LAM HA with Host Proteins

Homology modeling of LAM HA and its interacting host proteins was performed using the SWISS-MODEL server (https://swissmodel.expasy.org/) (accessed on 29 September 2024). The resulting structural models were validated using ProSA and ERRAT. Cluspro (https://cluspro.org/help.php (accessed on 29 September 2024)) [24] was employed to perform molecular docking between LAM HA and the successfully modeled interacting host protein. In the initial docking step, the PDB file of the target molecule was loaded, with the host protein designated as the receptor and LAM HA as the binding partner. The resulting interaction complex was visualized using PyMOL (version: 3.0.3), and amino acid residues involved in hydrogen bonding within a cutoff distance of 3.5 Å were identified and counted. The residue most frequently participating in hydrogen bond formation within the complex was predicted as the key amino acid (KeyAA). Prior to knockout experiments, the sequence conservation of KeyAA was analyzed based on the protein sequences listed in the Supplementary Data S1.

### 2.5. Cloning and Purification of Recombinant Proteins in E. coli

The wild-type LAM HA gene was excised from the pGBKT7-LAM HA plasmid by double digestion with BamHI and XhoI and subcloned into the corresponding sites of the pGEX-4T-1 vector to generate the recombinant plasmid pGEX-4T-LAM HA, which expresses the protein as a GST fusion protein. The ∆KeyAA LAM HA gene, lacking the codons encoding Ser83, Arg85, Tyr88, Asn124, and Lys192, was synthesized by Sangon Biotech (Shanghai, China) and cloned into pGEX-4T-1 using the same restriction sites, yielding the plasmid pGEX-4T-∆KeyAA LAM HA. Both constructs were verified by Sanger DNA sequencing. The recombinant plasmids pGEX-4T-LAM HA, pGEX-4T-∆KeyAA LAM HA, and the empty vector pGEX-4T-1 were transformed into *E. coli* BL21(DE3) competent cells. Single colonies were inoculated into LB broth containing 100 μg/mL ampicillin and cultured at 37 °C with shaking at 220 rpm until the optical density at 600 nm (OD600) reached 0.7. Protein expression was induced by adding isopropyl β-D-1-thiogalactopyranoside (IPTG) to a final concentration of 0.5 mM, and the cultures were further incubated at 16 °C for 5 h with shaking at 180 rpm. Cells were harvested by centrifugation at 10,000× *g* for 8 min at 4 °C, washed three times with phosphate-buffered saline (PBS), and resuspended in PBS. The cell suspension was sonicated on ice for 10 min using a Fisher Scientific™ Model 50 Sonic Dismembrator (Thermo Fisher Scientific, Waltham, MA, USA) with intermittent pulse cycles (5 s on/5 s off) and 1 min cooling intervals after every 2 min of sonication to prevent overheating. After centrifugation at 12,000× *g* for 10 min at 4 °C, the supernatant was collected and the GST-tagged recombinant proteins were purified using GST affinity magnetic beads (Beyotime, Shanghai, China) following the manufacturer‘s instructions. The purified proteins were then desalted by dialysis (Solarbio, Beijing, China), and their purity was assessed by SDS-PAGE.

### 2.6. Hemagglutination Test

LAM HA and ∆KeyAA LAM HA were diluted to 260 μg/mL. In a 96-well V-bottom microtiter plate (Corning, Corning, NY, USA), 25 μL of PBS was added to wells 2 through 8 in each row. The first well was added with 25 μL (containing 6.5 μg of protein) of LAM HA or ∆KeyAA LAM HA, and the solution was gently mixed by pipetting up and down three to five times. An aliquot of 25 µL of protein solution was transferred from the second well to the third well of the same 96-well plate. After mixing, 25 μL of protein was transferred from the second well to the third well, and then serially diluted two-fold to the seventh well. From the seventh well, 25 μL was removed and discarded. The eighth well served as the PBS control. Subsequently, 25 μL of PBS was added to each well, followed by 25 μL of 1% (*v*/*v*) chicken erythrocyte suspension. The plate was gently mixed and incubated at 37 °C for 40 min. The results were recorded when the erythrocytes in the control well formed a distinct button-like pellet. The hemagglutination titer was determined as the highest dilution showing 100% agglutination. To examine the effect of pH on erythrocyte agglutination, the pH of LAM HA and ΔKeyAA LAM HA was adjusted to approximately 6.0 and 5.0, and the experiment was repeated following the same procedure. All assays were performed in three independent replicates. Statistical comparisons between wild-type and mutant at the same pH were performed using the Mann–Whitney U test for independent samples. Comparisons across different pH conditions within the same genotype were analyzed using the Friedman test for repeated measures. All statistical analyses were conducted using GraphPad Prism (version 10.4.2).

### 2.7. Fourier Transform Infrared Spectroscopy

The secondary structures of the wild-type LAM HA and ∆KeyAA LAM HA sequences were predicted using the NovoPro online secondary structure prediction tool (https://www.novopro.cn/tools/secondary-structure-prediction.html (accessed on 15 October 2024)), and the resulting profiles were compared manually by visual inspection of the predicted α-helix, β-sheet, and random coil distributions. The proteins (5 mg/mL) at different pH values were analyzed using Nicolet™ iS50 FTIR spectroscopy (Thermo Fisher Scientific, Waltham, MA, USA). The dispersion medium was deionised phosphate-buffered saline. Scanning was performed using a scanning infrared spectrometer at points between 400 and 4000 cm^−1^. Subsequently, data between 1600 and 1700 cm^−1^ were extracted for a Gaussian fit using Peakfit 4.12 software (Systat Software Inc., San Jose, CA, USA). The contents of the α-helix, β-sheet, β-turn and random coil were then calculated and visualized by GraphPad Prism (version 10.2.0).

### 2.8. Conformational Analysis of LAM HA and Δ Key AA LAM HA at Different pH Values

Based on its amino acid sequence, the three-dimensional structure of the ΔKeyAA LAM HA deletion mutant was also modeled by homology modeling using the SWISS-MODEL server. The structures of LAM HA and ∆KeyAA LAM HA in different pH were analyzed by Amber software (Version Amber 25). The specific steps are as follows: in the obtained PDB file of each protein, HIS was changed to HID and named LAM HA pH 7.0 and ∆KeyAA LAM HA pH 7.0, respectively. HIS was changed to HIP and ASP was changed to ASH, and these were named LAM HA pH 6.0 and ∆KeyAA LAM HA pH 6.0, respectively. HIS was changed to HIP, GLU was changed to GLH, and ASP was changed to ASH, and these were named LAM HA_pH 5.0 and ∆KeyAA LAM HA pH 5.0, respectively. After loading the leaprc.protein.ff19SB force field and balancing the charges, the topology and trajectory files for each protein were successfully generated. Then the root mean square deviation (RMSD), root mean square fluctuation (RMSF), radius of gyration (Rg), solvent-accessible surface area (SASA) and hydrogen bond (Hb) analyses of each structure were analyzed by CPPTRAJ software package [25]. All results were derived from the equilibrated 100–200 ns portion of the trajectory (10,000 Frames). Mean values and standard deviations (SD) were calculated from the equilibrated last 50% of the simulation trajectories. Subsequently, qtgrace software (qtgrace_v026_Win7) was used for visualization.

## 3. Results

### 3.1. Genome-Wide Identification of Two Distinct Hemagglutinin Length Variants in M. synoviae

**Figure 1:**
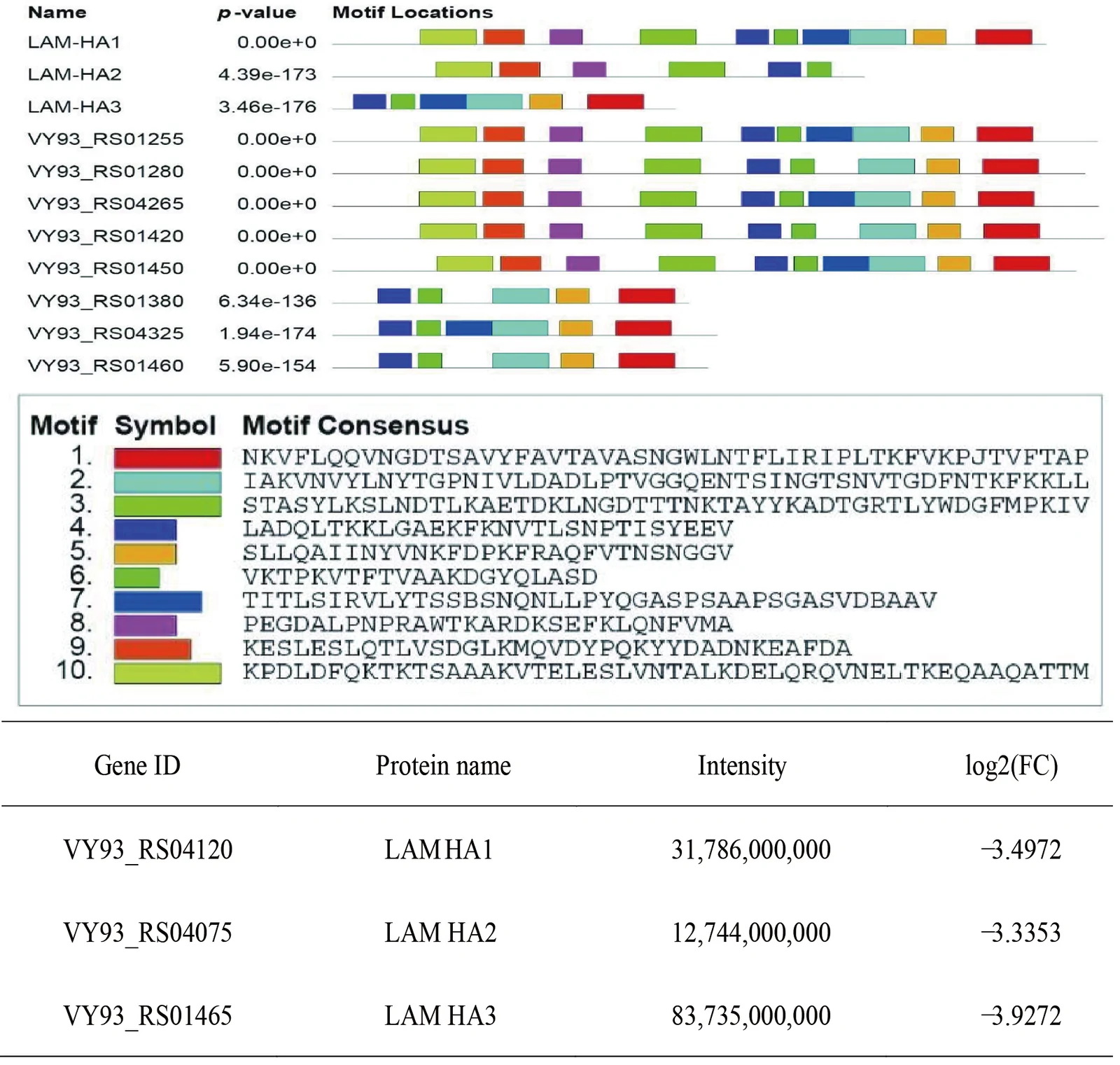
Domain organization of LAM HA proteins from *M. synoviae* strain WVU1853 identified by MEME. Each colored block represents a conserved motif. The first column lists the protein names (LAM-HA1–LAM-HA3) or NCBI locus tags (VY93_RS01255, VY93_RS01280, VY93_RS04265, VY93_RS01420, VY93_RS01450, VY93_RS01380, VY93_RS04325, VY93_RS01460). *p*-value in the second column (e.g., “4.39e-173” and “6.34e-136”) are automatically generated by MEME software and are embedded in the figure as the original, unmodified software output; lower p-values indicate higher statistical significance (greater confidence) for each motif. The third column shows the motif locations within each protein sequence, and the consensus sequences of the ten identified motifs (Motifs 1–10) are listed below.

Sequence analysis of hemagglutinins from 14 *M. synoviae* strains revealed two distinct length variants within this limited dataset: a long-chain form comprising 246–468 amino acid residues (average 341) and a short-chain form comprising 124–196 residues (average 155), with the number of ORFs per strain in the long-chain group (6–20) approximately twice that in the short-chain group (3–11) (Supplementary Data S2). While no conserved motifs were shared between the two groups, conserved structural features were observed within each group across the strains examined (Supplementary Data S3; Figures S1 and S2). Among the long-chain variants, three lipid-associated membrane hemagglutinins (NCBI accession numbers VY93_RS04120, VY93_RS04075, and VY93_RS01465) were identified. Of these, LAM HA (VY93_RS01465), which contains a complete C-terminal conserved domain, was selected for yeast two-hybrid screening of interacting host proteins (Figure 1). This observation, based on a limited dataset, is not intended for species-wide generalization, but rather served as a preliminary screen to identify conserved features in long-chain variants, providing the rationale for selecting LAM HA.

### 3.2. Yeast Two-Hybrid Screening Identifies 18 Host Proteins Interacting with LAM HA

**Figure 2:**
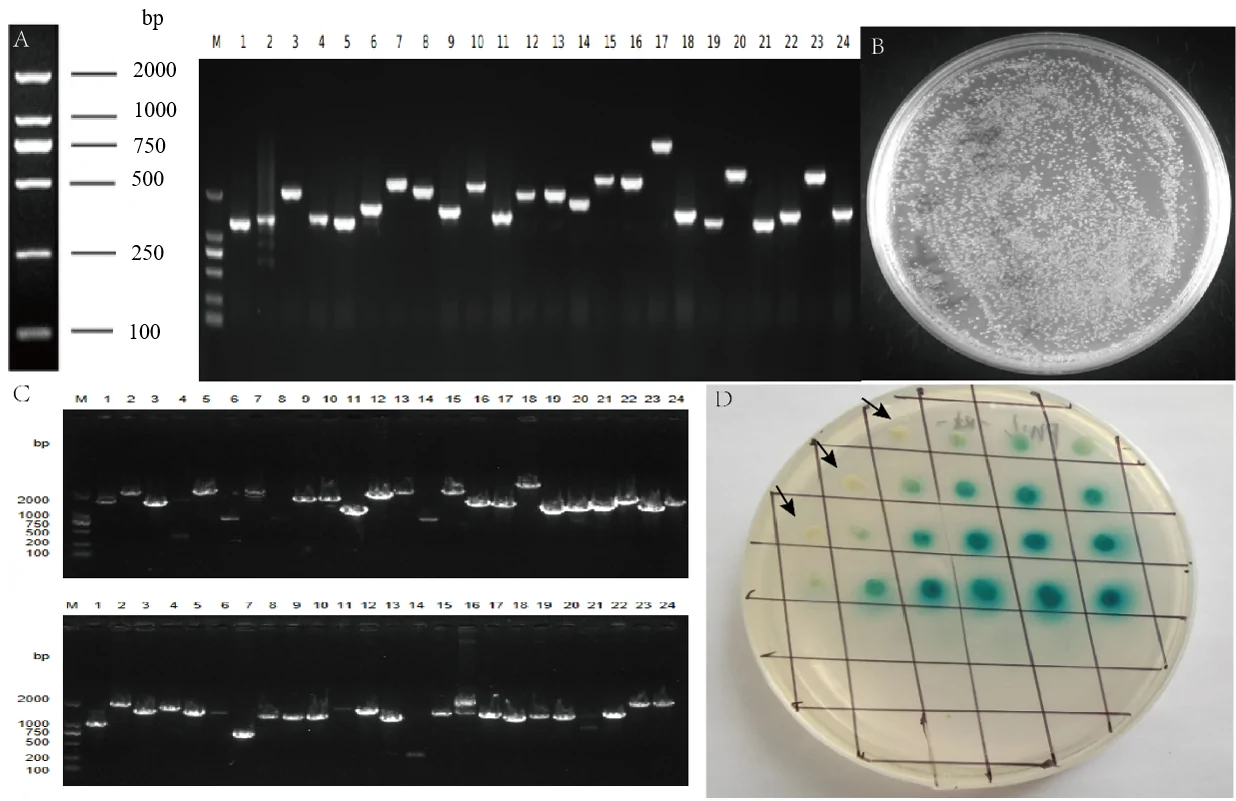
Construction and screening of the yeast two-hybrid cDNA library for LAM HA-interacting host proteins. (**A**) Determination of recombination rate via PCR analysis of insert fragments. Lanes 1–24 represent PCR products from randomly selected colonies. Inserts >1000 bp were counted as valid. (**B**) Library capacity determined from serial dilutions plated on LB medium. (**C**) PCR products from positive clone cultures. Lanes 1–24 show PCR products from candidate positive colonies. (**D**) Point-to-point verification. Arrows indicate three negative control colonies; all others are positive.

A yeast two-hybrid cDNA library was successfully constructed from host cells induced by *M. synoviae*, with a recombination rate of 100% (Figure 2A). The library capacity was determined to be 1.44 × 10^7^ CFU (Figure 2B). Yeast strain Y2HGold harboring pGBKT7-LAM HA was used as bait and screened against the cDNA library through three rounds of selection on dropout media. PCR products from positive clone cultures were analyzed to confirm the presence of insert fragments; only those with products greater than 500 bp were considered positive and subsequently sequenced for gene identification (Figure 2C). Subsequently, point-to-point verification confirmed 18 positive clones (Figure 2D). Sequencing and NCBI analysis identified 18 unique host proteins, covering cytoskeletal regulators (SDCBP, MAP4, ARHGAP12), membrane trafficking components (RUFY1, ERGIC2, ARFGAP2), ubiquitin-proteasome members (UBE2V2, PSMG2), redox/metabolic enzymes (PRDX6, ACADL, SPTSSA), and transcriptional regulators (ARID1B, CHAF1A) (Table 1 and Table 2).

### 3.3. Five Residues Critical for LAM HA-Mediated Hemagglutination Are Identified and Validated by Mutagenesis

**Figure 3:**
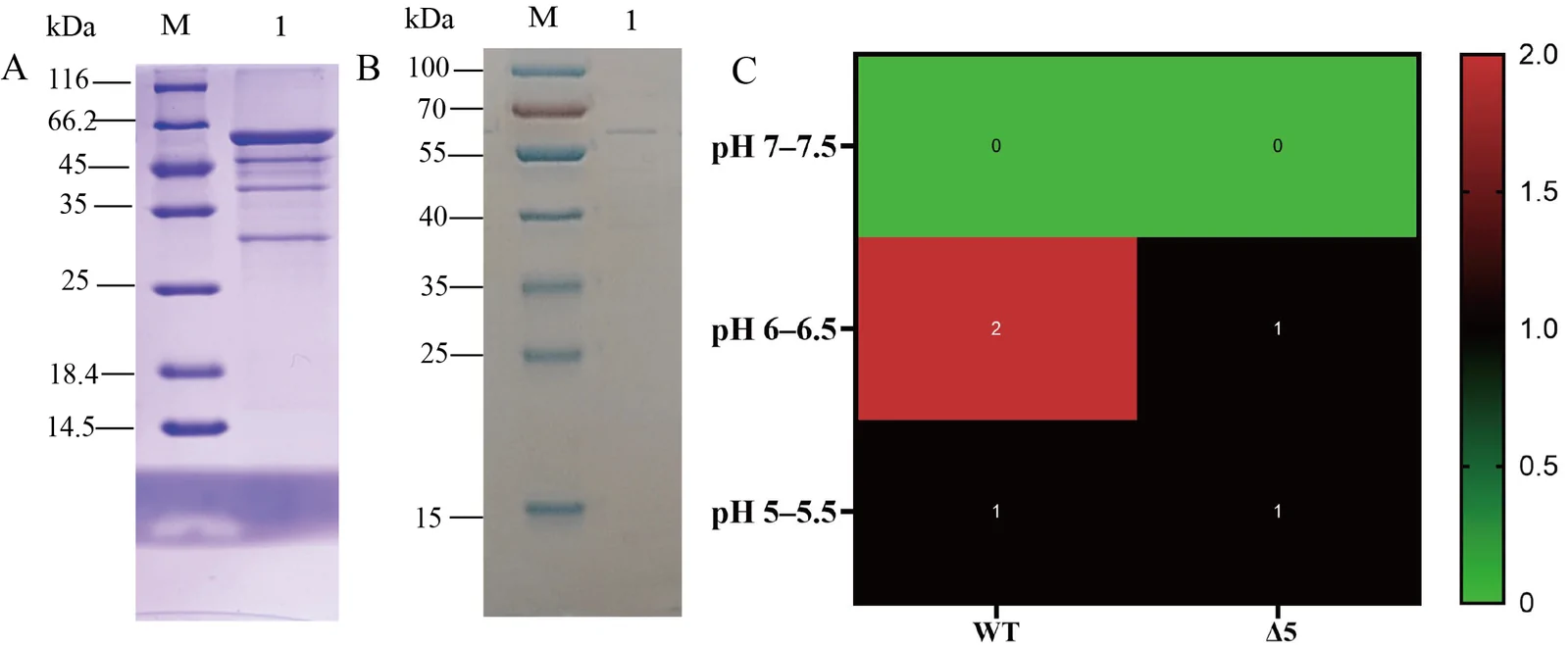
Identification of key amino acid residues in LAM HA involved in erythrocyte agglutination. (**A**,**B**) SDS-PAGE analysis of purified GST-tagged LAM HA proteins. (**Left**) wild-type; (**right**) ΔS83-R85-Y88-N124-K192 deletion mutant. M: protein marker; 1: recombinant protein. (**C**) Quantitative hemagglutination titers of wild-type LAM HA and the Δ5 deletion mutant under different pH conditions. Titers are expressed as reciprocal dilutions (e.g., 2 = 1:2). WT, wild-type; Δ5, ΔS83-R85-Y88-N124-K192 deletion mutant.

All generated homology models displayed good stereochemical quality, with ProSA Z-scores within the range of −5.0 to −10.0, ERRAT overall quality factors above 80%, and Clash Scores below 2.0, indicating good stereochemical quality of all generated models. Molecular docking analysis revealed that residues N15, S83, and Y88 participated in hydrogen bond formation on three occasions, while R85, N124, and K192 appeared four times each, identifying them as the most frequently interacting amino acid residues (Supplementary Data S4). To evaluate the evolutionary conservation of the five deleted residues, multiple sequence alignment of LAM HA was performed across representative *M. synoviae* strains. The analysis revealed that residues S83, R85, Y88, N124, and K192 are highly conserved among all examined strains (Supplementary Data S5). To validate their functional contribution, we constructed a pentuple deletion mutant (ΔS83-R85-Y88-N124-K192), which was successfully expressed and purified as a ~60 kDa protein (Figure 3B). Hemagglutination assays showed that wild-type LAM HA exhibited significantly higher activity at pH 6.0–6.5 (titer 1:2) compared to pH 7.0–7.5 (no activity) (*p* < 0.05). At the same optimal pH, the deletion mutant showed a reduced titer (1:1) compared to the wild-type (1:2) (*p* = 0.05). No significant differences were observed at pH 5.0–5.5 or between genotypes at other pH conditions (*p* > 0.05) (Figure 3C).

### 3.4. pH-Dependent Secondary Structure Changes Distinguish Wild-Type from Deletion Mutant LAM HA

**Figure 4:**
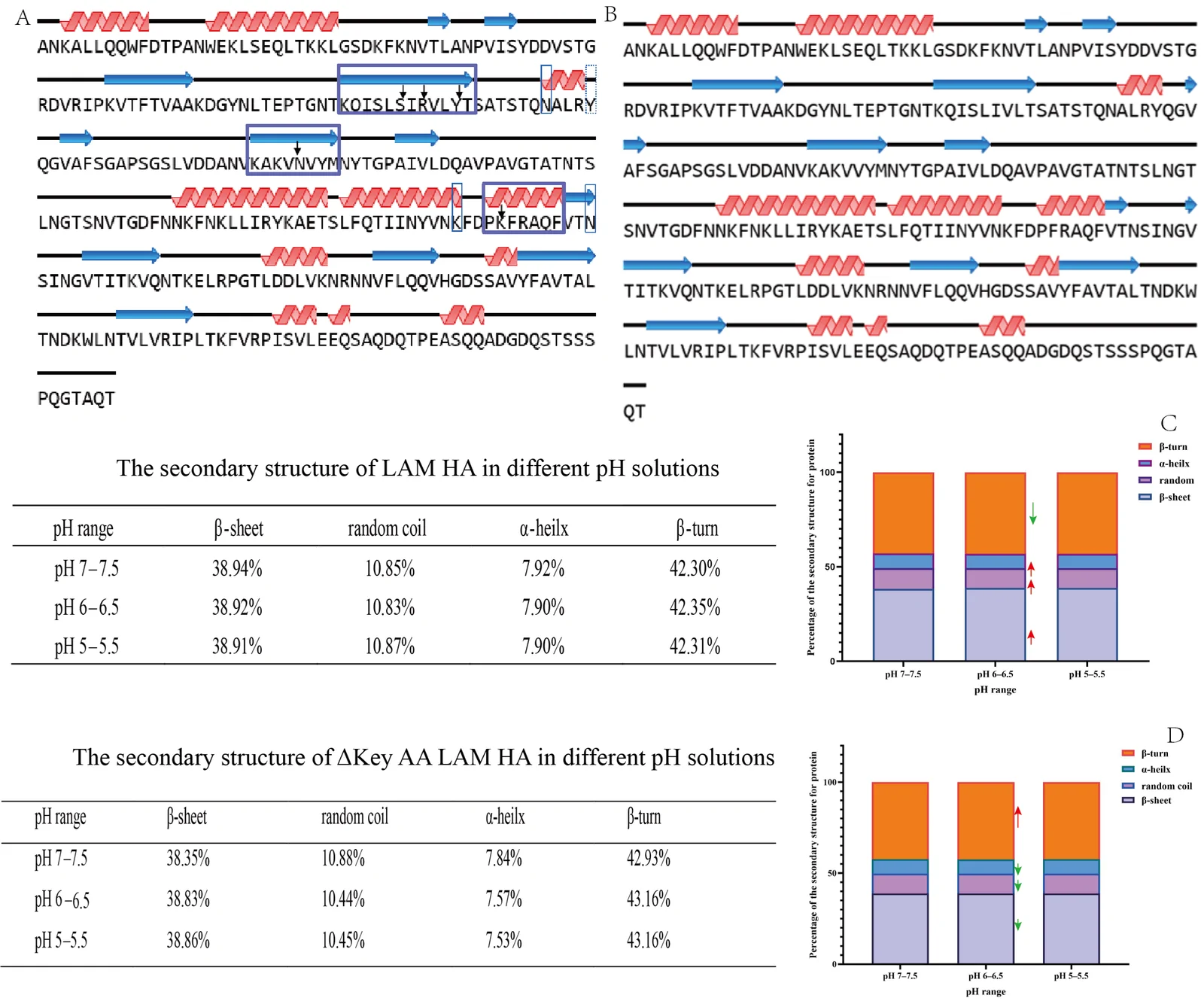
The effect of pH on the secondary structure of LAM HA and ΔS83-R85-Y88-N124-K192-LAM HA. (**A**) The secondary structure prediction of LAM HA. The arrow indicates the missing amino acid residue, and the purple box indicates the domain where the missing amino acid is located. The blue solid frame is an extra secondary structure, and the dotted frame indicates a secondary structure that is less extensive than that of ΔS83-R85-Y88-N124-K192 LAM HA. (**B**) The secondary structure prediction of Δ S83-R85-Y88-N124-K192 LAM HA. (**C**) Quantitative secondary structure composition of wild-type LAM HA. (**D**) Quantitative secondary structure composition of the deletion mutant. In panels (**C**,**D**), red arrows indicate structural components that are increased in the mutant relative to wild-type, while green arrows indicate components that are decreased.

Secondary structure prediction revealed that the sialic acid-binding motif PKVTFTVAAKDG predominantly adopts a β-sheet conformation, with residues S83, R85, Y88, and N124 located within this region, whereas residue K192 resides in an α-helical segment (Figure 4A). Deletion of residues S83, R85, Y88, N124, and K192 resulted in decreased α-helix content accompanied by increased β-sheet and random coil proportions (Figure 4B). At pH 6.0–6.5, circular dichroism spectroscopy further showed a reduction in α-helix content, a slight increase in β-turn structures, and a decrease in β-sheet content (Figure 4C). The deletion mutant showed a slight increase in β-turn structures (Figure 4D).

### 3.5. Molecular Dynamics Reveals pH-Dependent Flexibility Hotspots and Long-Range Conformational Coupling in LAM HA

**Figure 5:**
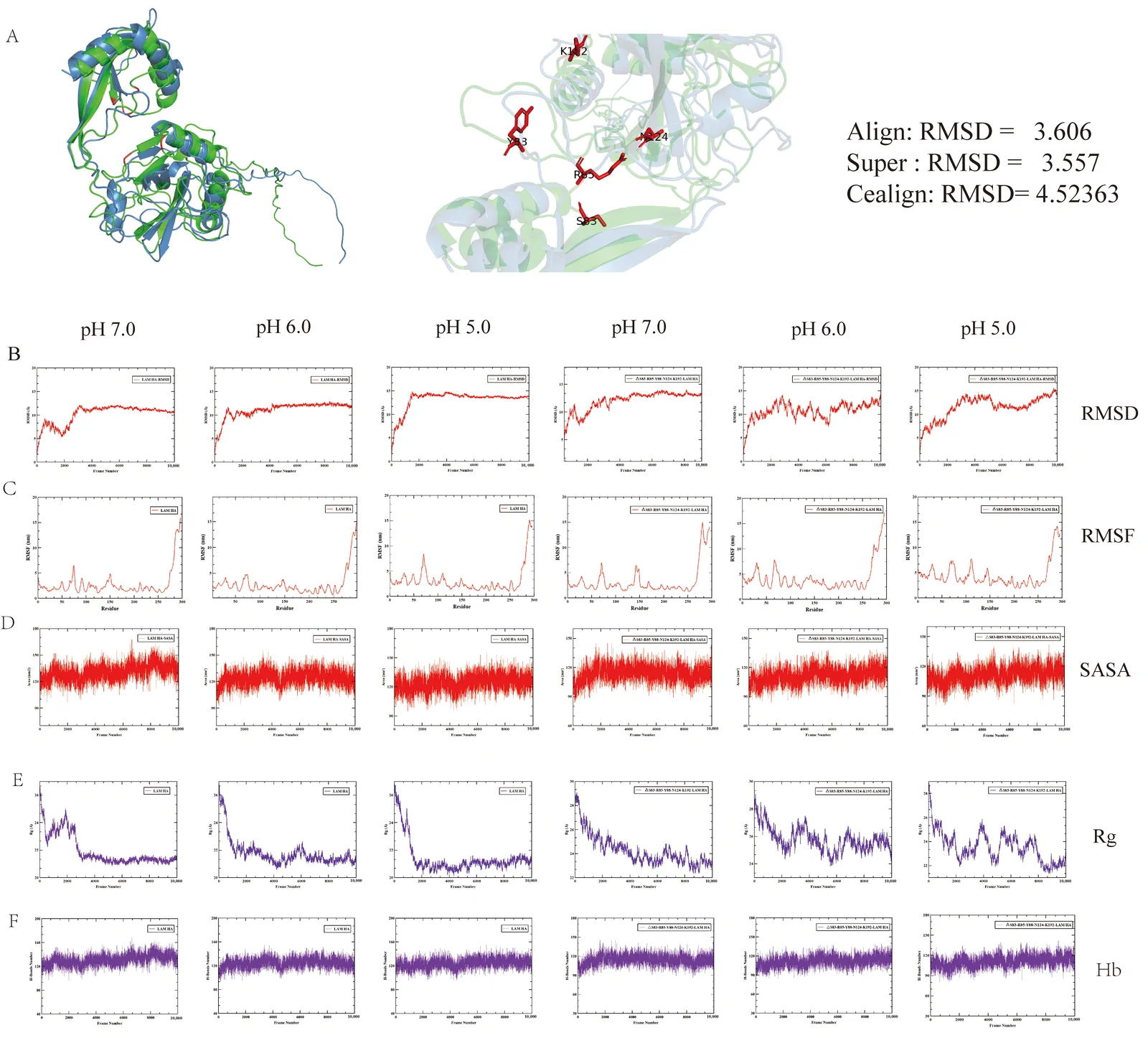
Structural dynamics of wild-type LAM HA and the ΔS83-R85-Y88-N124-K192 deletion mutant under different pH conditions (pH 5.0, 6.0, and 7.0). (**A**) Structural superposition of wild-type and mutant proteins based on PyMOL alignment; RMSD values are indicated. The distribution of Cα atomic distances (Peak Positions, δ) is shown for each alignment method (Align, Super, Cealign). (**B**) RMSD trajectories over MD simulations (x-axis: Frame number; y-axis: RMSD in Å). (**C**) RMSF per residue (x-axis: Residue number; y-axis: RMSF in Å). (**D**) Solvent-accessible surface area (SASA) (x-axis: Frame number; y-axis: SASA in nm^2^). (**E**) Radius of gyration (Rg) (x-axis: Frame number; y-axis: Rg in Å). (**F**) Number of intramolecular hydrogen bonds (Hb) (x-axis: Frame number; y-axis: Hb count).

PyMOL-based structural alignment revealed RMSD values exceeding 3 Å between the wild-type and the ΔS83-R85-Y88-N124-K192 deletion mutant, indicating substantial conformational differences (Figure 5A). Trajectory analysis showed that the deletion mutant exhibited consistently higher RMSD and RMSF values than the wild-type across all pH conditions, with a prominent flexibility peak in the 67–74 region (DGYNLTEPTG) at pH 5.0 that was absent at pH 6.0 (Figure 5B). The deletion mutant also displayed additional flexibility peaks in the 24–32 (TKKLGSDKF), 108–116 (PSGSLVDDANV), and 141–150 (GTATNTSLNGT) regions (Figure 5C). Quantitatively, the deletion mutant showed elevated RMSF (from 2.83 ± 1.88 Å to 4.18 ± 2.65 Å) and Rg (from 40.48 ± 1.31 Å to 54.16 ± 4.47 Å), with Rg abnormally increasing to 54.16 Å at pH 6.0–6.5, suggesting a cooperative unfolding event (Figure 5D,E). In contrast, RMSD showed a slight decrease in the mutant (11.17 ± 1.33 Å vs. 12.14 ± 0.21 Å). SASA analysis revealed that the deletion mutant exhibited nearly constant values (~113.5 nm^2^) across all pH conditions, in contrast to the pH-dependent behavior observed for the wild-type (125.63 ± 7.76 nm^2^ at pH 6.0–6.5; Figure 5D). Similarly, hydrogen bond counts remained largely unchanged in the deletion mutant (~113.5), further indicating disrupted pH responsiveness (Figure 5E). All statistical data are presented in Supplementary Data S6.

## 4. Discussion

The yeast two-hybrid (Y2H) system has been instrumental in dissecting pathogen–host interaction networks owing to its capacity to detect weak or transient protein associations [26,27,28]. To obtain a comprehensive host cDNA library that reflects infection-responsive gene expression, we constructed a Y2H library using RNA extracted from host cells infected with *M. synoviae* at multiple time points. Screening this library with LAM HA as bait identified 18 host protein interactors, which are enriched in cytoskeletal regulators (SDCBP, MAP4, ARHGAP12), endosomal trafficking components (RUFY1, ERGIC2, ARFGAP2), and ubiquitin-proteasome pathway members (UBE2V2, PSMG2)—functional categories commonly exploited by bacterial adhesins to promote host cell entry, intracellular survival, and immune evasion [29,30,31]. The presence of redox/metabolic enzymes (PRDX6, ACADL) and transcriptional regulators (ARID1B, CHAF1A) further suggests that LAM HA may modulate host metabolic states and gene expression to create a permissive environment for infection. Collectively, these interactions position LAM HA as a multifunctional adhesin that may coordinate multiple steps of the *M. synoviae* infection cycle, though these hypotheses require experimental validation. Given that experimentally determined complex structures are often lacking, computational modeling remains essential for understanding the molecular basis of these protein–protein interactions [24].

The hemagglutination assay used in this study serves as an in vitro indicator of the receptor-binding capacity of LAM HA. To identify the residues potentially responsible for this activity, molecular docking analyses were performed, which highlighted six residues—N15, S83, R85, Y88, N124, and K192—as candidate functional determinants. Of these, five conserved C-terminal residues (S83, R85, Y88, N124, and K192) were selected for combined deletion analysis to assess their collective contribution to hemagglutination. Molecular docking identified six candidate contact residues (N15, S83, R85, Y88, N124, and K192). However, N15 resides at the N-terminus of LAM HA and lies outside the functionally characterized C-terminal region. Since our study focuses on the conserved C-terminal cluster, N15 was excluded from further deletion analysis. S83, R85, and Y88 are located in β-sheet regions that, in other hemagglutinins, mediate heme binding and affect erythrocyte function [32,33], whereas N124 and K192 lie within α-helical structures, which are known to mediate protein adhesion [34]. Targeted deletion of S83, R85, Y88, N124, and K192 significantly reduced, but did not completely abolish, hemagglutination, confirming the importance of these residues while also suggesting that additional residues contribute to the overall activity.

The pH-dependent activity of LAM HA was confirmed by hemagglutination assays, with maximal binding at pH 6.0, reduced activity at pH 5.0, and complete loss at neutral pH—a profile reminiscent of viral fusion glycoproteins [35,36,37]. FTIR analysis showed that pH 6.0 drove maximal β-turn content and minimal random coil in both wild-type and deletion mutant proteins, indicating global conformational ordering. Despite this similar secondary structure propensity, the deletion mutant exhibited reduced hemagglutination activity, suggesting that β-turn formation alone is insufficient for full receptor binding. Since *M. synoviae* colonizes inflamed respiratory and synovial sites [38,39,40] (pH ~5.5–6.5), the optimal activity of LAM HA at pH 6.0 suggests that this acidic environment may promote its active conformation, facilitating host cell attachment and colonization, while its inactivity at neutral pH may favor persistence in inflamed niches. It should be noted that this pH range reflects local inflammatory conditions rather than systemic homeostasis. To elucidate the molecular basis of this pH-dependent behavior of LAM HA, MD simulations were performed under different pH conditions. A flexibility hotspot was identified in the 67–74 region (DGYNLTEPTG) of the wild-type at pH 5.0, which was absent at pH 6.0. This loop contains Asp/Glu residues (pKa ~4.0–4.5) that mediate electrostatic clamping when deprotonated at pH 6.0, restricting loop mobility; at pH 5.0, partial protonation relieves this constraint [18,41]. The deletion mutant exhibited elevated RMSF and Rg values across all pH conditions, with flexibility peaks extending to regions distal to the deletion site, indicating that Y88 functions as a long-range structural stability hub. Notably, Rg analysis revealed an abnormal increase in the deletion mutant at pH 6.0–6.5, suggesting cooperative unfolding that correlated with reduced hemagglutination. Unlike the wild-type, the deletion mutant displayed nearly constant SASA and hydrogen bond counts across all pH conditions, indicating disrupted pH responsiveness. Together, these findings demonstrate that pH 6.0 provides the optimal balance of conformational stability and flexibility, while the Y88 hub ensures proper spatial positioning of β-turn regions essential for full activity. Based on these results, we propose a dual-layer regulatory mechanism: a local pH-sensitive electrostatic switch at the 67–74 loop and a remote structural stability hub centered at Y88. At pH 6.0, both layers act synergistically—the loop is restrained by electrostatic clamping and the hub ensures its proper positioning—enabling optimal receptor binding. Disruption of either layer (pH deviation from 6.0 or hub deletion) compromises function. This mechanism may have biological significance, and the identified structural targets represent promising candidates for therapeutic intervention and vaccine development [42,43]. Several limitations of this study should be acknowledged, which also inform future directions. First, conventional MD simulations employ fixed protonation states and may not fully capture pH-dependent conformational changes, as the protonation equilibria of titratable residues are inherently coupled to local structural dynamics. Beyond this technical limitation, a major experimental constraint is the use of a combined deletion mutant of all five candidate residues. While this proof-of-concept strategy was chosen for rapid screening of the C-terminal cluster’s collective function before committing to single-point mutagenesis, it does not permit dissection of individual contributions or exclude the possibility that the observed functional impairment reflects global structural disruption rather than specific loss of individual contacts. Nevertheless, docking analysis supported cooperative effects among these residues, and future single-site mutagenesis will be required to define the hierarchical role of each residue. Additionally, constant pH MD simulations will be employed in future work to further explore the electrostatic switching mechanism governing the 67–74 loop, thereby overcoming the technical limitation of fixed protonation states in conventional MD simulations. These future studies will refine the structural targets for therapeutic and vaccine development and together with structure-guided approaches and rational mutagenesis for live attenuated vaccines, hold significant promise for combating *M. synoviae* infections.

## 5. Conclusions

In summary, our findings establish that LAM HA hemagglutination is governed by a dual-layer mechanism: a pH-sensitive electrostatic switch at the 67–74 loop and a structural stability hub centered at Y88. At pH 6.0, these layers synergize to provide optimal conformational balance for receptor binding, whereas disruption of either layer compromises function. The high conservation of the hub residues across *M. synoviae* strains further underscores their functional importance and supports Y88 as a conserved structural determinant. This architecture positions LAM HA among bacterial adhesins that exploit pH and long-range coupling for spatiotemporal control of adhesion.

## Figures and Tables

**Table 1:** Host proteins identified as LAM HA interactors by yeast two-hybrid screening.

Gene Name	Protein Name	Amino Acid Length (AA)
*SDCBP*	syndecan binding protein (SDCBP)	294
*ACADL*	acyl-CoA dehydrogenase, long chain (ACADL)	392
*HMBS*	hydroxymethylbilane synthase (HMBS)	358
*MAP4*	microtubule associated protein 4 (MAP4)	693
*RUFY1*	RUN and FYVE domain containing 1 (RUFY1)	632
*RPS21*	ribosomal protein S21 (RPS21), mRNA	83
*MRPL28*	mitochondrial ribosomal protein L28 (MRPL28)	251
*PRDX6*	peroxiredoxin 6 (PRDX6)	224
*SPTSSA*	serine palmitoyltransferase small subunit A (SPTSSA)	68
*ERGIC2*	endoplasmic reticulum-Golgi intermediate compartment protein 2 isoform X1	377
*UBE2V2*	ubiquitin-conjugating enzyme E2 variant 2 isoform 1	144
*ARHGAP12*	Rho GTPase activating protein 12 (ARHGAP12	746
*ARID1B*	AT-rich interaction domain 1B (ARID1B)	1341
*PSMG2*	proteasome assembly chaperone 2 (PSMG2)	273
*ARFGAP2*	ADP ribosylation factor GTPase activating protein 2 (ARFGAP2)	445
*CHAF1A*	chromatin assembly factor 1 subunit A (CHAF1A)	937
*CFAP20*	cilia and flagella associated protein 20 (CFAP20)	389
*MAP2K1*	mitogen-activated protein kinase kinase 1 (MAP2K1)	389

**Table 2:** Host proteins interacting with LAM HA and their potential roles in pathogenic processes.

Functional Category	Host Proteins	Potential Role in Pathogenic Processes
Cytoskeletal remodeling	SDCBP, MAP4, ARHGAP12	Promote host cytoskeletal rearrangement, facilitate invasion into non-phagocytic cells
Endocytic trafficking	RUFY1, ERGIC2, ARFGAP2	Exploit host endocytic pathways to enable intracellular survival and immune evasion
Protein synthesis and proteostasis	RPS21, MRPL28, UBE2V2, PSMG2	Modulate host protein homeostasis, potentially affecting antigen presentation and immune recognition
Metabolism and redox regulation	ACADL, PRDX6, SPTSSA	Regulate host metabolic microenvironment, resist oxidative stress
Transcriptional and chromatin regulation	ARID1B, CHAF1A	Modulate host gene expression, potentially suppressing immune responses
Signal transduction	HMBS, MAP2K1	Interfere with host signaling pathways to promote infection

## Data Availability

The data are available in the article or its Supplementary Materials.

## Supplementary Materials

The following supporting information can be downloaded at: https://www.mdpi.com/article/10.3390/microorganisms14092031/s1, Supplementary Data S1: Information on *M. synoviae* strains and hemagglutinin sequences. Supplementary Data S2: Sequence analysis of hemagglutinins from 14 *M. synoviae* strains. Supplementary Data S3: Conserved structural features of long- and short-chain hemagglutinins. Supplementary Data S4: Molecular docking analysis of interacting residues and hydrogen bonds. Supplementary Data S5: Multiple sequence alignment of LAM HA showing conserved residues S83, R85, Y88, N124, and K192. Supplementary Data S6: Statistical data for molecular dynamics simulations.
