# Supplementary material for: Residues Ser83, Arg85, Tyr88, Asn124, and Lys192 of C-Terminal Lipid-Associated Membrane Hemagglutinin Affect Mycoplasma synoviae Agglutination of Erythrocytes
Source: Microorganisms. 2026 Sep 12;14(9):2031. doi: 10.3390/microorganisms14092031 (PMC13609628; doi:10.3390/microorganisms14092031)
Supplement: Supplementary file 1 [file microorganisms-14-02031-s001.zip › Supplementary Data S1-Information on M. synoviae strains and hemagglutinin sequences.pdf]

Table S1. Information on the Mycoplasma synoviae strains used in this study.

**Strain**	**Submitting Institution**	**Country**	**Submission Year**	**Isolation Source**	**Host**	**Remarks**
WVU1853T	University of Florida	USA	2015	Not specified	Chicken (*Gallus gallus domesticus*)	Type strain (ATCC 25204)
5-9	Yangzhou University	China	2021	Not specified	Chicken	—
18DW	Konkuk University	South Korea	2021	Tracheal swab	Layer chicken (*Gallus gallus*)	—
51SH	Konkuk University	South Korea	2021	Tracheal swab	Layer chicken (*Gallus gallus*)	—
53	Not specified	Not specified	2005*	Not specified	Not specified	Published in J Bacteriol 2005
86079/7NS	University of Melbourne	Australia	2018	Not specified	Chicken	Formerly *M. synoviae* 7 NS
A4	Konkuk University	South Korea	2021	Synovial fluid	Layer chicken (*Gallus gallus*)	—
BS4S2	Konkuk University	South Korea	2021	Tracheal swab	Layer chicken (*Gallus gallus*)	—
FJ-01	Fujian Agriculture and Forestry University	China	2021	Synovial fluid	Chicken (*Gallus gallus*)	Isolated in Fuzhou, Fujian, China

Strain	Submitting Institution	Country	Submission Year	Isolation Source	Host	Remarks
G3	Konkuk University	South Korea	2021	Tracheal swab	Layer chicken (*Gallus gallus*)	—
HN01	Jiangsu Academy of Agricultural Sciences	China	2018	Not specified	Chicken	Isolated in Henan, China
MS-H	University of Melbourne	Australia	2015	Vaccine	Not specified	Vaccine strain (NTG-induced mutant)
NCTC10124	Wellcome Trust Sanger Institute	UK	2018	Arthritic joint	Chicken	Type strain (NCTC 10124)
WF18	Shandong Academy of Agricultural Sciences	China	2021	Articular cavity	Broiler	Isolated in Jinan, Shandong, China

---

Notes:

- The submission year for strain "53" is inferred from its publication year (2005).
- All strains are currently classified under the genus *Mycoplasmopsis* (formerly *Mycoplasma*).
- Abbreviations: T = type strain; NCTC = National Collection of Type Cultures; ATCC = American Type Culture Collection.
- "Not specified" indicates that the information was not provided in the available record.
- "*M. synoviae*" refers to the former name *Mycoplasma synoviae*.
- NTG = N-methyl-N'-nitro-N-nitrosoguanidine (chemical mutagen).

>LAM HA

ANKALLQQWFDTPANWEKLSEQLTKKLGSDKFKNVTLANPVISYDDVSTGRDVRIPKVT
FTVAAKDGYNLTEPTGNTKQISLSIRVLYTSATSTQNALRYQGVAFSGAPSGSLVDDANVK
AKVNVYMNYTGPAIVLDQAVPAVGTATNTSLNGTSNVTGDFNNKFNKLLIRYKAETSLFQ
TIINYVNKFDPKFRAQFVTNSINGVTITKVQNTKELRPGTLDDLVKNRNNVFLQQVHGDSS
AVYFAVTALTNDKWLNTVLVRIPLTKFVRPISVLEEQSAQDQTPEASQQADGDQSTSSSPQ
GTAQT

Mycoplasmopsis synoviae ATCC 25204 strain WVU1853T

>VY93_RS01155-320
MLALYWDGFMPKIVLEGFDSRWTNVNDNKSKLQTWFNTKANWSN
LEDQLVKKLGSEKFKNVVLSQPNVTLETFTWNRQTWGVPTVTFNLAAKEGYEKAKNST
NTIALKIRVVYNSNNTNEILFPIQGASSSAAPSGANTADHAKTIKDVNVYLNYTGPAI
VLDATLPEVGKVDNTTTNGTSNVTDDLNTKFKDLVTSDQRTASLFFQAIQKYVNTFDP
KYPANPVSDQKNGVTWARVENKALKIANLNNPNAVFVQQMKDDSEAVYLPVIGVANNR
WLNTFLIRIPLTKFVKPLTVFTATSASTPTQDSSASNTSPQTTT

>VY93_RS04290-334
MLLIYWDGFMPKIVVDDGYVEDTEENNKENNKNTNQQKLEAWFK
ANQDKFTLVADQLTRKLGSDKFKNVTLTNPQVSYEEVTVNSNTWKNPKVTFNIQTKPG
YQLTEPTTEESQISLSIRVLYSSQESNQNLLTIQGASPTAAPSGSSVDDAKVKAKVNV
YMNYTGPNIVLDADLPTVGGKENTSINGTSNVEGTFNDKFKELLVDNNRVPREFLSTV
INYVNKFDPKFRAQLVTDTNGVAITSVQSKKELRIGNLNDLLYNKKVFLQQVNGDSSA
VYFAVNGVTSEGWLNTFLIRIPLTKFVRPLTAFTAPPASAPTQTDSGTGAQTQPEAQS

>VY93_RS01190-362
MLLIYWDGFMPKIVIDDYVADGYDVANNAENNRAQHEAANRPLL
EQWFKVNQDKLSLVAEQLTKKLGEEKFKNMTLSNPEISWDEVKFSNKVSNKGYLTPKV
TFNLTPKEDYKKASDSSETVTLTIRNLYKEANSDTNVFATQGASSSTTPNNASQANDA
TTIEKVNVYLNYTGSNIELDADLPRVGEQENTSLNGTSNVTDDFNNKFNKLLIRYKAE
SSLFQAIINYVNKFDPKFRAQLVTDINGVTVTKVEKIKELRPGTLDDLLKNRNNVFLQ
QMKNDTKAVYFAVTAIASNGWLNTFLIRIPLTKFVKPLSVFTAPVVTPPAEEATGGSQ
TQEARLQPAGYSNSDSSGSTEQTPPSTT

>VY93_RS01205-358
MLIKFWDGFMPKIVVQGYEADGEEKGKATHKEANQKKLEDWFAA
NQDKLSLVADQLTRKLGKDKFKNMVLSTPTITWDEVEFSNKGSNKVYLTPKVTFNLAA
KEGYALAQDSATSVTLTIRVLYKDSNLTRNVLSTQGAYYTAAPKNAIVDDANVKAKVN
VYMNYTGPNIELDADLPTVGGKENTSINGTSNVEGTFNDKFKKLLVNVVGESITQTSL
LQAIINYVNKFDPKFPAEFVLKTNGVTVASVQNNTQLRIGSLNDFLYNNKGFLQQVNT
DTKAVYFAVMGVTSNGWVNTFLIRIPLTKFVRPISVLEEQTTEEETQESQEGQENQEQGST
TQTNTESSGTGQSSDLTEVSA

>VY93_RS04305-360
MLSLYWDGFMPKITLESFTASQTNVEDNKSKLQKWFNTKANWSN
LEDQLVKKLGSKKFKNVKLTNPQVDWDIVNSNQYRPKVTFDVTQKEGYELQSGSEFSQ
SLTMVIRVLYTTQSPNANVLQMQGASSLAVPNDTTTVSDANVLRTVNVYLNYTGPNIV
LDADLPTVGGQENTSINGTSNVTGAFNNAFRGNPTSGLLFTNRYPNPLLKSVINYVNK
FDPKFKAEFVTNSKNGVTITKVEKNKKQKREELRPGTLDDLLSNKDIVFLQQIKGDTE
AVYFAVSAITNDRWLNTVLVRIPLTKFVRPISVLEAPQQEETQEDGEEMQQGNQDGTQ
AGQTDETAGSGTPQASSPQTMPNSSQ

>VY93_RS01340-362
MLLIYWDGFMPKIVIDDYVADGYDVANNAENNRAQHEAANRPLL
EQWFKVNQDKLSLVAEQLTKKLGEEKFKNMTLSNPEISWDEVKFSNKVSNKGYLTPKV
TFNLTPKEDYKKASDSSETVTLTIRNLYKEANSDTNVFATQGASSSTTPNNASQANDA
TTIEKVNVYLNYTGSNIELDADLPRVGEQENTSLNGTSNVTDDFNNKFNKLLIRYKAE
SSLFQAIINYVNKFDPKFRAQLVTDINGVTVTKVEKIKELRPGTLDDLLKNRNNVFLQ
QMKNDTKAVYFAVTAIASNGWLNTFLIRIPLTKFVKPLSVFTAPVVTPPAEEATGGSQTQEA
RLQPAGYSNSDSSGSTEQTPPSTT

>VY93_RS04145-322
MLLIYWDGFMPKIVVDDGYVEDTEENNKENNKNTNQQKLEAWFK
ANQDKFTLVADQLTRKLGSDKFKNVTLTNPTISWDEVRFSKGNVTKLYLTPKVTFNLAAK
EGYALAQDSATSVTLTIRVLYKDSNPEVNVFQTQGSSPSATPNGANSANHAQTIKD
VNVYLNYTGPNIELNAELPKVGSQENTSLNGTSNVDGDFNTKFKKLLVNVVKEGHAES
SLFQAIINYVNKFDPKFRAQFVTNSINGVTITKVQNTKELRPGTLDDLVKNDRDNVFLQQ
MKNDTEAVYFAVSAIASNGWLNTFLIRIPLTKFVKPLTEFQAQS

>VY93_RS01370-367
MLLIYWDGFMPKIVIDDYVADGYDVANNAENNRAQHEAANRPLL
EQWFKVNQDKLSLVAEQLTKKLGEEKFKNMTLSNPTISWDEVRFSKGNVTKLYLTPKV
TFNLAAKEGYALAQDSATSVTLTIRVLYKDSNPEVNVFQTQGSSPSATPNGANSANHA
QTIKDVNVYLNYTGSNIELDADLPRVGEQENTSLNGTSNVDGDFNTKFKKLLVNVVKEG
HAESSLFQAIINYVNKFDPKFRAQFVTNSINGVTITKVQNTKELRPGTLDDLLKNRN
NVFLQQMKNDTKAVYFAVTAIASNGWLNTFLIRIPLTKFVKPLSVFTAPVVTPPAEEATGGS
QTQEARLQPAGYSNSDSSGSTEQTPPSTT

>VY93_RS04310-383
MASTASYLKSLSDSLKAETDKLNGDTTENKTAYYKVDTGRTLYW
DGFMPKIVVQGYVADTTGSSKDTNENANRPLLQQWFEANQDKLSLVADQLTKKLGSDK
FKNVVLTSPMVSYEDTSSNTRVPKVTFTLQGKEGYELQTTDGASATLSLSIRVLYTSE
NSTQNVLRYQGASSSAAPNRASNPNNATTIKDVNVYLNYTGPNIELNAEAPTVGSQDN
TSINGTSNVDGDFNTKFKNLLVNVVGGDHAESSLFQAIINYVNKFDPKYRAAFVTNSR
DGVTITKVQNTKELRPGTLDDLLSNKNKVFLQQIKGDTEAVYFAVTAIASNNWLNTFLIRI
PLTKFVRPISVLEEQSAQDQTPEASQQAPEGGSLASGTAPTEGTQS

>VY93_RS04325-354
MLALYWDGFMPKIVVEGYVASDDARVDEKTHEDANTKLLQQWFD
TPANWEKLSEQLTKKLGADKFKNVTLSSPKVTYETISTGKNVKIPKVTFTLQGKEGYN
LQIDNGASQSLTLLIRVLYTSADATENALRYQGASSSTAPGSPTVNDANVKAKVNVYL
NYTGPSIVLDEALPTVGGKENTLINGTSNVEGTFNDKFKKLLVNVVGENITQTSLLQA
IINYVNKFDPKFPASFVLKTNGVAIASVQNNTQLRIGSLNDFLWNKKGFLQQVNGDSS
AVYFAVNGVTSQGWLNTFLIRIPLTKFVKPLTEFRPTTHTSPSSDTQQEQTEQTGGNQDASG
GASESGTASQPQQAAS

>VY93_RS04245-342
MLLIYWDGFMPKIVLDNFDSGWTNDLDNQNKIKAWFGEAENWEG
LSDQLTKKLGTERFKNVVLSNPTISVETVTLSSGSVKFPKVTFRVAAKDGYQLASGEG
TTTTLELKIRVLYSSSNPNTLLLPYQGASPSAAPAGSTPSNNAAVIRDVNVYMNYTGP
NIELDADLPRVGEQENTSINGTLNVDGDFNTKFKKLLVNVVGESITQTSLLQAIINYV
NKFDPKFPAEFVLKTNGVALTSVQSGTQLKIGSLNDFLYNNKGFLQQVNGDSSAVYFA
VTAIASNNWLNTFLIRIPLTKFVKPVTAFMPAAPIAPAQQESQENTQTTQGSQQAPEGGSST
SGTG

3864
11, 320-383

>VY93_RS01455-151
MLLIYWDGFMPKIVLEGFDSTWGRNSDNQDKIRAWFGDAANWVG
LSDQLTKKLGAETFKNVKLTYKEVTFSTNAVKTPTVTFTVEAQDGYTLDNSANEISLVVR
VLYNRGSENVIQLPRQGASSSAAPRGATVNDANVKAKVNVYLNYTDI

>VY93_RS04660-196
MLNLYWDGFMPKIVLEGFDKTWEQNSEENERKIRKWFDNAANWV
GLSDQLTKKLGAERFKNVKLTYKEVTFSTNAVKTPTVTFTVEAQDGYTLDNSANEISL
VVRVLYNRGSENVIQLPTQGASSSAAPAGSTPSNNAAVIRDVNVYMNYTGPALILNAALPT
VGNAANTSLNGTSNVTDEDFNAALEDCYLPIDM

>VY93_RS01260-156
MLALYWDGFMPKIVLTDFDKTWGQNSEENERKIRKWFDNAANWA
SLSEQLTKKLGAERFKNVKLTFKSARFSTDPAKVPTVTFTVAAKEGYTLTTGDNASAT
EISLVVRVLYNRESESTIQMPTQGASSSAAPRGATVNDAKAKAKVNVYLNYTDI

>VY93_RS01295-157
MLLLYWDGFIPKIVLDNFDKTWGQNTANQDKIRVWFRDAANWAG
LSDQLTKKLGAETFKNVKLTFKEASFSGENNIKTPTVTFTVAAKEGYTLTTGDNASAT
EISLVVRVLYNRGSENVIQLPRQGASSSATPNNAKNANDKTTKRTVNVYLNYTDI

NASATEISLVVRVLYNRGSENVIQ

>VY93_RS01315-170
MLALYWDGFMPKIVVNDYKIVVDGYVTNTDEVKKEADKTTNQEK
LQNWFNDPANWKKLAEQLTKKLGEEKFKNMVLSTPTITWDEVEFSGTHYLTPKATFNL
ATKEGYALAQDSANTVTLTIRVLCKDSNPEVNVFQTQGSSPSAAPSNSSVNDANVKAKVN
VYLNYTDI

830
5, 151-196

Mycoplasmopsis synoviae strain 5-9

>LA081_RS01110-384
MSEEVAAEAETQTPAPTPAADLASTASYLKSLNDTLKAETDKLN
GDTTENKTAYYKADTTRTLYWDGFMPKIVVQGYQADGAGNGKATHEAANRPLLQQWFE
TPANWEKLSEQLTKKLGSDKFKNVVLTAPTVSYEDGGSNTRIPKVTFTLQGKDGYILQ
TDNGATSSLTLSIRVLYTSASSTQNALRYQGASSSAAPSGANTADHAKTIKDVNVYLN
YTGPAIVLDATLPVVGKVDNTTTNGTSNVTDDLNTKFKDLVTSDQRTASLFFQAIQKY
VNTFDPKYPANSISDQKNGVTWARVENKALKIANLNNPNAVFVQQMKDDSEAVYLPVI
GVANNRWLNTFLIRIPLTKFVKPLTVFTATPASTPTQDSSASNTSPQTTT

>LA081_RS01125-343
MLIKFWDGFMPKIVVQGYQADGYVADGQGNNRAAHEDANKTKLQ
NWFNAPANWEKLSEQLTKKLGADKFKNVTLSSPTVTYETISTGRDVRIPKVTFTVTVK
NGYNLIAPEGDAKQISLSIRVLYTSASSTENALRYQGVSFSATPKDASVENPATAIKN
VNVYLNYTGPSIVLDQAVPAVGTANNTSINGTSNVEGTFNTKFKKLLVNVVRGGHAES
SLFQAVINYVNKFDPKFRAQFVTNSINGVTITKVESGTQLRPGTLDDLVKNDRNNVFL
QQMKNDTEAVYLPVTALTNDKWLNTVLVRIPLTKFVKTLSVFQATTAAPQDAAQTSEA
QGSQPAGQS
PEGDAKQISLSIRVLYTSASSTENA

>LA081_RS01135-325
MPTYEPDTTDDNKAAHEEANKTKLEAWFTNSANWAGLEDQLTKR
LGSDKFKNVKLTYASLTLTDFTASRGVTRWNPTVKFSIKANEGYTLDTNSTKEIDLVV
RVLYKSAEANAANVFTHEGASSSAAPKNTMTPNNAEVIKNVNVYLNYTGPSIVLDEAL
PTVGGKENTSINGTSNVDGAFNNAFRGNPSKGLLFTDRYPNPLLKSVINYVNKFDPKY
RATFVTDSRDGVTISNVTNNVQLRPGTLDDLLSNKNKVFLQQIKGDTEAVYFAVTAIA
SNNWLNTFLIRIPLTKFVRPLTEFQAQSEAAPTEETSGEQDSAPTGTGL

>LA081_RS01140-343
MLALYWDGFMTKIVLEGFDSRWTNVNDNKSKLQTWFNTKANWSN
LEDQLVKKLGSEKFKNVVLSQPNVTLETFTWNRQTWGVPTVTFNLAAKEGYEKAKNST
NTIALKIRVVYNSNNTNEILFPIQGASSSAAPSGANTADHAKTIKDVNVYLNYTGPAI
VLDATLPKVGKVDNTTTNGTSNVTDDLNTKFKDLVTSDQRTASLFFQAIQKYVNTFDP

KYPANSISDQKNGVTWARVENKALKIANLNNPNAVFVQQMKDDSEAVYLPVIGVANNR
WLNTFLIRIPLTKFVKPLTVFLLLQLGLLLKIHQLQIQAHKQQLKVHNNKTKNMPQNV
FLILRRVYT

>LA081_RS01145-370
MLIKFWDGFMPKIVIDDYVADGYDVANNAENNRAQHEAANRPLL
EQWFKVNQDKLSLVAEQLTKKLGEEKFKNMTLSNPEISWDEVKFSNKVSNKGYLTPKV
TFNLTPKEDYKKASDSSEKVTLTIRNLYKEANSDTNVFATQGAYYTATPSDNATTIKK
VNVYLNYTGPNIELDADLPRVGEQENTSLNGTSNVTDDFNNKFNKLLIRYKAETSLFQ
TIINYVNKFDPKFPAKFVTNSINGVTITKVQSDTQLRPGTLDDLLKNRNNVFLQQMKN
DTKAVYFAVNGVTSQGWLNTLLIRIPLTKFVRPISVFTAPVVTPPTQDDSQEDSSNAG
NTNENTETPVEDQTGQESSESRSTQTDQAQQESPQT

>LA081_RS01150-347
MPKIVVEDYVSDGDGNNRAQHEAANQQKLQEWFNTSSNWEKLSE
QLTKKLGADKFKNVTLTNPQVSYEEVTVNSNAWKNPKVTFNIQAKPGYQLTEPTTEES
QISLSIRVLYSSQESNQNLLTIQGASPTAAPNNTTMVSDPNVLRTVNVYLNYTGPNIE
LDAELPQVGNQNNTSINGTSNVEGTFNDKFKKLLVNVVGESITQTSLLQAIINYVNKF
DPKFPASFVLKTNGVAIASVQNNTQLRIGSLNDFLYNNKGFLQQVNTDTKAVYFAVMG
VTSNGWVNTFLIRIPLTKFVRPISVLEEQTTEEETQESQEGQENQEQGSTTQTNTESS
GTGQSSDLTEVSA

>LA081_RS01190-357
MPKIVVEDYQPAGHGNNKDTNETTNQQKVQEWFNTSSNWEKLAE
QLTKKLGANKFKNVVLTAPTVSYEDGGSNTRIPKVTFTLHGKDGYTLQTDNGATSSLT
LSIRVLYTSASSTQNALRYQGASSSAAPSGSTPSNNAAVIRNVNVYMNYTGPNIELDT
ELPQVGNQNNTSINGTSNVDGDFNTKFKNLLVNVVGGSFIQTSLLQAIINYVNKFDPK
FRAAFVTSINGVALTSVQNDTQLRIGSLNDFLYNNKGFLQQVNGDSSAVYFAVNGVTS
EGWLNTFLIRIPLTKFVRPITPFTASAVMIPTTEDYQEDSSNAGNTNENTETPVEDQT
GQESSESGSTQTDQAQQESPQTQ

>LA081_RS01220-382
MALYWDGFMPKIVVSLLTTKAANQEYDASVRQGGVVFWKKRNGE
ILDVWFAANQDKFTLVADQLTKKLGEEKFKNMTLSNPTITWDELTIPEGTILTPKVTF
TLTPKDGYTLADNSENTVTLTIRNLYKAADPNTNLFATQGASPSAAPKNTKTPNDAEV
IKNVNVYLNYTGPSIVLDQDLPTVGGQENTSINGTSNVTGDFNNAFRGNPSSGLLFTN
RYPNPLLKSVINYVNKFDPKFRAEFVTNATNGVTITKVQSGTQLRPGTLDDLNKNNVF
LQQIKGDTEAVYFAVSAIASNGWLNTFLIRIPLTKFVRPISVLQTPDQQQTQEGGEEA
TQPQNAGSGTASTSEVQSGQSNGPVGSGTGSTQQAQSSSTTGTSSSSN

>LA081_RS01235-368
MLNLYWDGFMPKIVVDDGYVEDTEENNKENNKNTNQQKLEAWFA
ANQDKFTLVADQLTRKLGEEKFKNMTLSNPTISWDESVWKTKTILTPKVTFNLTPKEG
YEKATGSLETVTLTIRNLYKEANPDTNVFATQGASSSATPNNASQANDATTIEKVNVY

LNYTGPSIVLDEALPTVGATPNTSINGTSNVTGDFNTKFKKLLVSGTTQTPLLQTIIN
YVNKFDPKFPASFVLKTNGVAIASVQNNTQLKIGSLNDFLWNNKGFLQQVNTDTKAVY
FAVIGVASNGWLNTFLIRIPLTKFVRSITPFTAPAVMTPTTEDSQEESRENSSQADNT
EQNAENQADGQSGGEATETQEAQGDSASQSSPQT

>LA081_RS01240-309
MLSLYWNGFMPRIVVEGYQPAGHGSNKEAHETTNQNQEKLQNWF
NAPANWEKLAEQLTKKLGADKFKNVTLTNPIVTYEEITVDKYTWKNPKVTFAVEAKPG
YQLASGAGTSTTLELKIRVLYTSESSTQNLLTIQEASSSAAPSGANTADHAKTIKDVN
VYLNYTGPAIVLDTTLPEVGNANNTIINGTSPITDETLAAKVKTLFNKTTNTNDPTEL
MLAITKYVQTFDPKYLSTRTDNGYRAWVSWMWTNRSALSQRELKQPADIFLQQMKDDT
EAVYLPIGGTTDDKWLNTFLIRIPLNKFVKAIL

>LA081_RS01255-350
MALYWDGFMPKIVLTDFDKTWGQNTANQTKIRDWFREASNWEGL
SDQLTKKLGVERFKNVVLSQPNVTFEIINWNGQTWRVPTVTFNLAAKEGYELIGSTNT
IALKIRVVYDSNDANAILFPIQGASSSAVPNRASNPNDATTIKNVNVYLNYTGPAIVL
NADLPTVGGQENTSINGTSNVDGTFNTAFRGNPSSGLLFTHRYANPLLKSVINYVNKF
DPKFRAEFVTDSKNGVTISNVTNNVQLRPGTLDDLLSNKNKVFLQQIKGDTEAVYFAV
NGVTSEGWLNTFLIRIPLTKFVRPLTEFQAQSATVPTQEGQTTQGQENSAGSGALGST
TGTQTTSGGSSQASGS

>LA081_RS01275-349
MLALYWDGFMPKIVVDDGYVEDTEENNKENNKNTNQKKLEAWFK
ANQDKFTLVADQLTRKLGEEKFKNMTLSNPTISWDEVKFSKGNVTKLYLTPKVTFNLA
AKEEYEKSSDSLETVTLTIRNLYKETNPDTNVFATQGASSSATPNNASQANDATAIEK
VNVYLNYTGPSIVLDEALPTVGATPNTSLNGTSNVTGEFNTKFRKLLVSVFRQGHAES
SLFQAVINYVNKFDPKYRATFVTNSINGVTITKVQNTKELRPGTLDDLVKNDRNNVFL
QQIQRDTEAVYLPVTAVTIDKWLNTVLVRIPLTKFVRSITPFTAPVVTPPAEEATEGS
QTQASTPQTMPNSSQ

>LA081_RS01290-349
MLALYWDGFMPKIVVDDGYVEDTEENNKENNKNTNQKKLEAWFK
ANQDKFTLVADQLTRKLGEEKFKNMTLSNPTISWDEVKFSKGNVTKLYLTPKVTFNLA
AKEEYEKSSDSLETVTLTIRNLYKETNPDTNVFATQGASSSATPNNASQANDATAIEK
VNVYLNYTGPSIVLDEALPTVGATPNTSLNGTSNVTGEFNTKFRKLLVSVFRQGHAES
SLFQAVINYVNKFDPKYRATFVTNSINGVTITKVQNTKELRPGTLDDLVKNDRNNVFL
QQIQRDTEAVYLPVTAVTIDKWLNTVLVRIPLTKFVRSITPFTAPVVTPPAEEATEGS
QTQASTPQTMPNSSQ

>LA081_RS01345-339
MLALYWDGFMPKIVVEGYEADGEGNGKATHEDANKALLQQWFDT
PANWEKLSEQLTKKLGSDKFKNVTLANPVISYDDVSTGRDVRIPKVTFTVAAKDGYNL
TEPTGNTKQISLSIRVLYTSATSTQNALRYQGVAFSGAPSGSLVDDANVKAKVNVYMN

YTGPAIVLDQAVPAVGTATNTSLNGTSNVTGDFNNKFNKLLIRYKAETSLFQTIINYV
NKFDPKFRAQFVTNSINGVTITKVQNTKELRPGTLDDLVKNRNNVFLQQVHGDSSAVY
FAVTALTNDKWLNTVLVRIPLTKFVRPISVLEEQSAQDQTPEASQQADGDQSTSSSPQ
GTAQT

4915
14, 309-384

>LA081_RS01120-169
MLALYWDGFMPKIVVDDGYVENTEENNKENNKNTNQQKLEAWFK
ANQDKFTLVADQLTRKLGEEKFKNMTLSNPTISWDEVKFSKGNVTKLYLTPKVTFNLA
AKEGYEKASDSSETVTLTIRNLYKEANPDTNVFATQGASSSAAPRGATVNDTNVKAKV
NVYLNYTDI

>LA081_RS01165-173
MPKIVVEGYQPTSHGRNKENDETTNQNQEKLQNWFNDPANWEKL
SEQLTKKLGADKFKNVTLSSPKVTYETISTGSEVKIPKVTFTVTAKDGYNLTAPEGDT
KQISLSIRVLYTSASSTQNVLRYQGVSYSGSPGGANIANHAQTIKDVKVYLNYTGPAI
VLNADLPAVGTTW

>LA081_RS01185-157
MLSLYWDGFMPKIVLEGFDSTWGRNSENQTKIREWFGNAANWEG
LSDQLTKKLGAERFKNVKLTYKEVTFSTNAVKTPTVTFTVAAKDGYTLVDSGKNASST
EISLVVRVLYNRDNESIIQLPTQGASSSAAPNNAKNANDKTTKRTVNVYLNYTDI

>LA081_RS01205-157
MLALYWDGFMPKIVLTDFDKTWEQNSEENERKIRKWFDNAANWV
GLSDQLTKKLGAERFKNVKLTYKEVTFSTNAIKTPTVKFTVAAKEGYTLTTGDNASGT
EISLVVRVLYNRGSENVIQLPTQGASSSAAPNNAKNANDKTTKRTVNVYLNYTDI

>LA081_RS01270-162
MLALYWDGFMPKIVLEGFDSTWRENSEENERKIREWFREASNWA
GLSDQLTKKLGAERFKNVKLTFKEASFSSDNIKTPTVTFTVAAKDGYTLTTGDNASAT
EISLVVRVLYNHDDESTIQLPTQGASSSAAPNTASANNPALAIKNVNVYLNYINQFKL
TN

>LA081_RS01285-162
MLALYWDGFMPKIVLEGFDSTWRENSEENERKIREWFREASNWA
GLSDQLTKKLGAERFKNVKLTFKEASFSSDNIKTPTVTFTVAAKDGYTLTTGDNASAT
EISLVVRVLYNHDDESTIQLPTQGASSSAAPNTASANNPALAIKNVNVYLNYINQFKL
TN

>LA081_RS01300-161
MLALYWDGFMPKIVLEGFDSTWRENSANQDKIRAWFGDAANWAG

LSDQLTKKLGAERFKNVKLTFKEASFSSDNFKTPTVTFTVAAKDGYTLTTGDNASATE
ISLVVRVLYNHDDESTIQLPTQGASSSAAPNTASANNPALAIKNVNVYLNYINQFKLT
N

>LA081_RS01320-157
MPKIVVDDGYVEDTEENNKENNKNTNQKKLEAWFGDAANWAGLE
DQLTKRLGSDKFKNVKLTYTSVTLTDFTAGRGVTRWNPTVKFSVKANEGYTLDTNSTK
EIDLVVRVLYKSAEANAANVFTHEGASSSATSRGATFNDPNVKSKVNVYLNYTDI

>LA081_RS01325-149
MPRIVLSPLPEEVARANKEYDASIRQGGVVFWKTRNGEILDVWF
AANQDKFTLVADQLTRKLGEEKFKNMTLSNPTISWDEVKFSKGNVTKLYLTPKVTFNL
AAKEGYEKATDSLEKVTLTIRNLYKEANPDTNVFATQGTASSSVSEV

>LA081_RS01335-165
MLSLYRDGFMPKIVVSGYEADGAGVNKAQHEEANKTKLQQWFAT
ESNWEKLSEQLTKKLGADKFKNVKLTYKSITLTDFTVDRATRWYPTVKFSVEANEGYT
LNTNSTKEIDLVVRVLYKSAEANAANVFTHEGASSSATPNNAKNANDKTTKRTVNVYL
NYTDI

>LA081_RS01365-166
MPKIVTTALPTTVAPMNREYNESPTGGHEFWKGRNKQVLDAWFA
ANQDKLSLVAEQLTKKLGKDKFKNMVLSTPTITWDEVEFSGRRYLTSKVTFTLTPKDG
YEKVSDSSETVTLTIRNLYKEANPDTNIFATQGTSSSAAPDNTTEPNNATVIKNVNVYLNYT
DI

>LA081_RS01250-147
MPKIVLTDFDKTWGQNSEENERKIREWFREASNWAGLSDQLTKK
LGAERFKNVKLTYKEVTFSTNAVKTPTVTFTVAAKDGYTLTTGDNASATEISLVVRVLYNH
DDESTIQLPTQGASPSAAPSGSSVNDAKVKAKVNVYLNYTDI

1763
12,147-173

Mycoplasmopsis synoviae strain 18DW

>K6987_RS02520-383
MQTAPAAETPAADLASTASYLKSLSDSLKAETDKLNGDTTENKT
AYYKTDTTRTLYWDGFMPKIVVPGYEAVDAGANKAQHETENTRLLQQWFDTPANWEKL
SEQLTKKLGSDKFKNVVLTAPTVSYEKVTVGRYTWKNPKVTFNIQAKPGYQLTEPTTN
PKQISLTIRVLYENQTSTQNLLTIQGASPSAVPSDANAENTSPTVKNVNVYLNYTGPN
IELDAELPTVGSQDNTSINGTSNVTGAFNTAFRGLLFTKRLVNPLMQSVINYVNKFDP
KYRAEFVTDSKNGVTITKVQKNRELRPGTLDDLNNNNVFLQQIKGDTEAVYFAVTAIA
SNNWLNTFLIRIPLTKFVKPLTVFTAPPASAPTQTDSGTGAQTQPEAQS

>K6987_RS02560-359
MLALYWDGFMPKIVVKDYQPAGHGNNKDTNETANKTKLQEWFAT
SSNWEKLSEQLTKKLGSDKFKNVTLTNPQVSYEEVTVNTYTWKNPKVTFNIQAKPGYQ
LTEPTTNPKQISLTIRVLYSSQESNQNLLTIKGASPSEAPSNSSVDDANVKAKVNVYM
NYTGPDIVLDQAVPAVGTANNTSLNGTSNVTGEFNTKFKKLLVNVVSRGHAESSLFQA
VINYVNKFDPKFRAQFVTNSINGVTITKVQNTKELRPGTLDDLVKNDRDNVFLQQIQG
DTEVVYLPVTALTNDKWLNTVLVRIPLTKFVRPLTEFQDQSATAPTQEGQTTQGQENS
AGSGALGSTTGAQTPSGGSSQASGS

>K6987_RS02565-357
MLALYWDGFMPKIVVDGYVADGAGANKAQHEDANKTKLQEWFKT
SSNWEKLAEQLTKKLGASKFKNVVLTAPTVSYEDDESNTRIPKVTFTLRGKDGYTLQT
DNGASQSLTLSIRVLYTTENQTENALKYQGASASASAVPNNTTTVNDENVSRTVNVYL
NYTGPAIVLDQVVPAVGTAANTLINGTSNVDGAFNTAFRGKLSKGLLFTNRYANPLLK
SVINYVNKFDPKYRAEFVTNSINGVTITKVQNTKELRPGTLDDLDKDNVFLQQIQGDT
EAVYFAVTAIASNNWLNTVLVRIPLTKFVRPLTEFQDQSATAPTQEGQTTQGQENSAG
SGALGSTTGAQTPSGGSSQASGS

>K6987_RS02575-321
MPKIVVQGYVADTTEDNKEEIKEANKTKLQNWFKAPANWEKLSE
QLTKKLGADKFKNVKLTYKSITLTDFTVAGATRWNPTVKFSVEANEGYTLDTNSTREI
DLVVRVLYKSAEANAANVFTHEGASPSAAPGNTKTPNDPKVIKNVNVYLNYTGPSIVL
DEALPTVGSQDNTSINGTSNVTSDFNNAFRGNPSSGLLFTDRYPNPLLQSVINYVNKF
DPKYRAAFVTDSKNGVTITKVQNTRELRPGTLDDLNGNNVFLQQMKNDTEAVYLPVTA
IASNGWLNTVLVRIPLTKFVRPVAPFAPAAPAQPQGGQTETSQPA

>K6987_RS02595-353
MPKIVVPTYEPDTTDDNKAAHEEANKTKLEAWFTNSANWAGLED
QLTKRLGSDKFKNVKLTYKSITLTDFTVDGATRWNPTVKFSVKAKDDYALDSTSASEI
DLVVRVLYKSAEANAANVFTHEGASSSAAPKNTKTPNDPKVIKNVNVYLNYTGPSIVL
DEALPTVGSQDNTSINGTSNVTGDFNNAFRGNPSSGLLFTDRYPNPLLKSVINYVNKF
DPKFRATFVTDSKNGVTISNVTDKVQLRSGTLDDLLSNKNKVFLQQIKGDTEAVYFAV
TAIASNNWLNTVLVRIPLTKFVRPVAPFAAAAPAEPNSDTQEQAGEIGGSQDGSGSAS
KSGTEGQSSPSTGTSSSSN

>K6987_RS02600-416
MADLASTVSYLKSLNDTLKATTDALNGDNPTTKTAYYKADTGRT
LYWDGFMPKIVTTLLEGETNYREYNPKPLTGSFIFVPNRNKRIIAQWLRNNPDKLALV
AEQLTRKLGKDKFKNMTLTNPVISMDELIRSEGRYYTPKVTFNLTPKDGYTLADNSEN
TVTLTIRNLYKEANPDTNVFATQGTSFSAVPSGSSVDDANVKAKVNVYLNYTGPSIVL
DQDLPTVGTANNTSINGTSNVTGDFNTKFKNLLINVVGESITQTSLLQAIINYVNKFD
PKFPASFVLNTNGVALTSVQSGTQLRIGSLNDFLYNNKGFLQQVHGDSTAVYFAVNGV
TSQGWLNTFLIRIPLTKFVKPVTVFTPPVVTPPKQDDSQEDSSNAGNTNENTETPVED

QTGQESSESGSAQTDQAQQESPQT

>K6987_RS02615-334
MPKIVIDDYVADGYHVANNTKNNRAQHEAANRPLLEQWFKVNQD
KLSLVAEQLTKKLGEEKFKNMTLSNPEISWDEVKFSNKVSNKGYLTPKVTFNLTPKED
YKKASDSSEKVTLTIRNLYKEANSDTNVFATQGAYYTATPSDNATTIKKVNVYLNYTG
PNIVLDADLPRVGEQENTSLNGTSNVTDDFNNKFNKLLIRYKAETSLFQTIINYVNKF
DPKFKAAFVTSINGVAIASVQNGTQLRIGNLNDLLWNNKVFLQQVNGDSSAVYFAVNG
VTSEGWLNTFLIRIPLTKFVRPLTEFQAQSPAAPTEEASGDQAQADPQATQMGTQAQS

>K6987_RS02620-336
MVTETIEHNTKLQINKNYKNDLTPQAIEKKLSEQLTKKLGADKF
KNVTLTNPQVSYEEVTVNSNAWKNPKVTFNIQAKPGYQLTEPTTEESQISLSIRVLYS
SQESNQNLLTIQGASPTAAPNNTTMVSDPNVLRTVNVYLNYTGPNIELDAELPQVGNQ
NNTSINGTSNVEGTFNDKFKKLLVNVVGESITQTSLLQAIINYVNKFDPKFPASFVLK
TNGVAIASVQNNTQLRIGSLNDFLYNNKGFLQQVNTDTKAVYFAVMGVTSNGWVNTFL
IRIPLTKFVRPISVLEEQTTEEETQESQEGQENQEQGSTTQTNTESSGIGQSSDLTEV
SA

>K6987_RS02625-328
MPKIVIDDYVADGYHVANNTKNNRAQHEAANRPLLEQWFKVNQD
KLSLVAEQLTKKLGEEKFKNMTLSNPEISWDEVKFSNKVSNKGYLTPKVTFNLTPKED
YKKASDSSEKVTLTIRNLYKEANSDTNVFATQGAYYTATPSDNATTIKKVNVYLNYTG
PNIVLDADLPTVGGKENTSINGTSNVEGTFNDKFKELLVDNNRVPREFLSTVINYVNK
FDPKFRAQLVTDTNGVAITSVQSKKELRIGNLNDLLYNKKVFLQQVHGDSSAVYFAVN
GVTSEGWLNTFLIRIPLTKFVRPLTAFTATPASAPTQTDSGTGAQTQPEAQS

>K6987_RS02630-347
MPKIVVEDYVSDGDGNNRAQHEAANQQKLQEWFNTSSNWEKLSE
QLTKKLGADKFKNVTLTNPQVSYEEVTVNSNAWKNPKVTFNIQAKPGYQLTEPTTEES
QISLSIRVLYSSQESNQNLLTIQGASPTAAPNNTTMVSDPNVLRTVNVYLNYTGPNIE
LDAELPQVGNQNNTSINGTSNVEGTFNDKFKKLLVNVVGESITQTSLLQAIINYVNKF
DPKFPASFVLKTNGVAIASVQNNTQLRIGSLNDFLYNNKGFLQQVNTDTKAVYFAVMG
VTSNGWVNTFLIRIPLTKFVRPISVLEEQTTEEETQESQEGQENQEQGSTTQTNTESS
GIGQSSDLTEVSA

>K6987_RS02655-338
MLALHWDGFMPKIVVPTYEPDTTDDNKAAHEEANKTKLEAWFTN
SANWAGLEDQLTKRLGSDKFKNVKLTYASVTLTDFTAGRGVTRWNPTVKFSVKANEGY
TLDTNSTKEIDLVVRVLYKSAEANAANVFTHEGASSSAAPKNTMTPNNAEVIKNVNVY
LNYTGPSIVLDQDLPTVGGKENTSINGTSNVDGAFNTAFRGNPSKGLLFTNRYVNPLL
QSIINYVNKFDPKYRAEFVTDARDGVTISNVTNNVQLRPGTLDDLDKNNVFLQQIKGD
KEAVYFAVTAVASNNWLNTFLIRIPLTKFVKPLTEFRPTTPTSPSSDTQQQGTAQTQS
NQGS

>K6987_RS02660-328
MLALYWDGFMPKITLESFTASQTNVEDNKSKLQTWFNTKANWSN
LEDQLVKKLGSEKFKNVVLSQPNVTLETFTWNRQTWGVPTVTFNLAAKEGYEKAKNST
NTIALKIRVVYNSNNTNEILFPIQGASSSAAPNGANTADHAKTIKDVNVYLNYTGPAI
VLDATLPVVGKVDNTTTNGTSNVTDDLNTKFKDLVTSDQRTASLFFQAIQKYVNTFDP
KYPANPISDQKNGVTWARVENKALKIANLNNPNAVFVQQMKDDSEAVYLPVIGVANNR
WLNTFLIRIPLTKFVKSLTVFTATPASAPTQPEAYNQEASTSGTAPQTTQGS

>K6987_RS02665-324
MLALYWDGFMPKIVVPGYEADGAGNGKATHEAANRPLLQQWFET
PANWEKLSEQLTKKLGSDKFKNVVLTAPTVSYEKVTVGKYTWKNPKVAFNIQAKPGYE
LTEPTTNPKQISLTIRVLYSSQESNQNLLTIQGAYPTAAPSDANADNPASAIKNVNVY
LNYTGPAIVLNADLPEVGKVDNTTINGTSNVNGAFNNAFRGNPTSGLLFTNRYPNPLL
KSVINYVNKFDPKYRAEFVTDARDGVTITKVQNTRELRPGTLDDLNGNNVFLQQIKGD
TEAVYFAVSAIASNGWLNTFLIRIPLTKFVRPLTEFQAQSSPTTGTSS

>K6987_RS02670-334
MLIKFWDEFMPKIVLEGFDKTWEQNSEENERKIRKWFDNAANWV
GLSDQLTKKLGAERFKNVKLTYKEVTFSTNAIKTPTVTFTVEAQDGYTLTTGDNASAT
EISLVVRVLYNRGSENVIQLPTQGASSSAAPAGSTPSNNAAVIRDVNVYMNYTGPSIV
LDEAHPTVGATPNTSINGTSNVIGDFNTKFKKLLVNVLSGGSFTQTSLLQAIINYVNK
FDPKFPASFVLKTNGVAIASVQNNTQLRIGSLNDFLYNNKGFLQQVNTDTKAVYFAVN
GVTSQGWLNTFLIRIPLTKFVKPLIEFRPTTPTNTSSDTQQQDTQQQGTAQTQSNQGS

>K6987_RS02675-380
MLALYWDGFMPKIVLTDFHKTWEQNSEENERKIREWFDNAVNWA
GLSDQLTKKLGAERFKNVKLTYKEVTFSTNAIKTPTVTFTVAAKDGYTLVDSGENASA
TEISLVVRVLYNRDNENTIQMPTQGASSSAVPNRASSPNDETTIKTVNVYLNYTGPSI
VLDAELPQVGSQDNTSINGTSNVTGAFNTAFRGNPSRGLLFTNRYPNPLLKSVINYVN
KFDPKYRAEFVTNSKNGVTITKVQSNTQLRPGTLDDLVKNRDNVFLQQVHDDSSAVYF
AVNGVGDNKWLSTFLIRIPLTKFVKPLTEFRPVVPSTPASPENSEEETQQDSQENNEA
EQGTTGQTENNSTEEATEDSGSGSSNTDSSQNSDQQSQQSPQSQQQ

>K6987_RS02690-385
MLNLYWDGFMPKIVVDDYVADTTENNKEEIKEANKTKLQNWFKA
PANWEKLSEQLTKKLGADKFKNVKLTYKSITLTDFTVDRATRWYPTVKFSVEANEGYT
LNTNSTREIDLVVRVLYKSAKSSAANVFTHEGASPSAAPSDSSVDDANVKAKVNVYLN
YTGPSIVLDEAHPTVGGQENTSINGTSNVEGTFNDKFKELLVSVLSGGHAQSSLFQAI
INYVNKFDPKFRAAFVTNSKNGVTITKVENGTQLRPGTLDDLVKNRDNVFLQQVHDDS
SAVYFAVNGVGDNKWLSTFLIRIPLTKFVKPLTEFRPVVPSTPASPENSEEETQQDSQ
ENNEAEQGTTGQTENNSTEEATKDSGSGSSNTDSSQNSDQQSQQSPQSQQQ

>K6987_RS02730-339

MLALYWDGFMPKIVVEGYEADGEGNGKATHEDANKALLQQWFDT
PANWEKLSEQLTKKLGSDKFKNVTLANPVISYNDVSTGRDVRIPKVTFTVAAKDGYNL
TEPTGNTKQISLSIRVLYTSATSTQNALRYQGVAFSGAPSGSLVDDANVKAKVNVYMN
YTGPAIVLDQAVPAVGTATNTSLNETSNVTGDFNNKFNKLLIRYKAETSLFQTIINYV
NKFDPKFRAQFVTNSINGVTITKVQNTKELRPGTLDDLVKNRNNVFLQQVHGDSSAVY
FAVTALTNDKWLNTVLVRILLTKFVRPISVLEEQSAQDQTPEASQQEDGDQSTSSSPQ
GTAQT

>K6987_RS02740-300
MPKIVLTDFDKTWAQNSENEGKIREWFGFATVSNWATLSDQLTK
KLGAERFKNVKLTFKEASFSSDNIKTPTVTFTVAAQDGYTLDNSTNEISLVVRVLYNR
DNESTIQLPTQGDSSSAAPAGSTPSNNAAVIRDVNVYMNYTGPALILNAALPTVGNAA
NTSLNGTSNVTDEDFNAAFRGLLFTNRYVNPLMQSVINYVNKFDPKYRAMFVTNAIDG
AALTKVQNKKELRPGNLEDLLYNNNVFLQQMKNDTEAVYFAVTALTIDKWLNTVLVRI
LLTKFVRPLTEFQAQSSPTTGTS

6262
19， 300-416

>K6987_RS02540-161
MLNLYWDGFMPKIVLEAFDSTWRENSANQTKIREWFREASNWAS
LSDQLTKKLGAERFKNVKLTFKNATFVGTNIKTPTVKFTVKAKEGYTLTTGDNASATE
ISLVVRVLYNRGSESTIQMPTQGASSSAAPNTASADNPASAIKNVNVYLNYIKQFKLT
N

>K6987_RS02585-166
MLALYWDGFMPKIVVQDYVADTTENDKKEANKTKNQEKLKAWFG
DAANWVGLEDQLTKRLGSDKFKNVKLTYKSITLTDFTVAEATRWNPTVKFNVKANEGY
TLDTNSAKEIDLVVRVLYNSAEANAANVFTHEGASSSAAPGNTKTPNNAEVIKNVNVY
LNYTDI

>K6987_RS02610-156
MLALYWDGFMPKIVLTDFDKTWEQNSENERKIREWFDNATNWAT
LSEQLTKKLGAERFKNVKLTYKEVTFSTNAVKTPTVTFTVAAKEGYTLVDSGENASAT
EISLVVRVLYNRDNESTIQMPTQGASSSAAPRGATVNDANVKAKVNVYLNYTDI

>K6987_RS02645-152
MPKIVVEGYQPTSHGRNKENDETTNQNQEKLQNWFNDPANWEKL
SEQLTKKLGADKFKNVTLSSPKVTYETISTSRDVRIPKVTFTVAAKDGYNLTAPTGGT
KQITLSIRVLYTSQNQTENALRYQGVSSSAAPNRAVEQTMFKLLKMLTFT

>K6987_RS02685-159
MLIKFWDGFMPKIVLEGFDKTWEQNSEENERKIRKWFDNAANWV

GLSDQLTKKLGVERFKNVKLTFKEASFSISSDNIKTPTVTFTVEAQDGYTLTTGDNAS
ATEISLVVRVLYNRGSENVIQLPTQGASSSAAPGNPTEPNNATVIKNVNVYLNYTDI

>K6987_RS02705-157
MPKIVVDDGYVEDTEENNKENNKNTNQKKLEAWFGDAANWAGLE
DQLTKRLGSDKFKNVKLTYTSVTLTDFTAGRGVTRWNPTVKFSVKANEGYTLDTNSTK
EIDLVVRVLYKSAEANAANVFTHEGASSSATSRGATFNDPNVKSKVNVYLNYTDI

>K6987_RS02710-149
MPRIVLSPLPEEVARANKEYDASIRQGGVVFWKTRNGEILDVWF
AANQDKFTLVADQLTRKLGEEKFKNMTLSNPTISWDEVKFSKGNVTKLYLTPKVTFNL
AAKEGYEKATDSLEKVTLTIRNLYKEANPDTNVFATQGTASSSVSEV

>K6987_RS02750-175
MLALYWDGFMPKIVTTALPTTVAPMNREYNESPTGGHEFWKGRN
KQVLDAWFAANQDKLSLVAEQLTKKLGKDKFKNMVLSTPTITWDEVEFSGRRYLTSKV
TFTLTPKDGYEKVSDSSETVTLTIRNLYKEANPDTNIFATQGTSSSAAPDNTTEPNNA
TVIKNVNVYLNYTDI

1275
8,149-175

Mycoplasmopsis synoviae strain 51SH

>K6989_RS02395-332
MPKIVVDGYVADGYDVANNAENNRAQHEAANRPLLEQWFKVNQD
KLSLVAEQLTKKLGEEKFKNMVLSTPTISWDEVKFSNRLYLTPKVTFNLAAKEEYEKS
SDSLETVTLTIRNLYKAADPNTNLFATQGASSSAVPNRASSPNDETTIKTVNVYLNYT
GPNIELNAELPKVGFQENTSLNGTSNVTDDFNNKFNKLLIRYKAETSLFQTIINYVNK
FDPKYRATFVTDSKNGVTISNVTDKVQLRHGTLDDLNKNNVFLQQIKGDTEAVYFAVT
AIASNNWLNTFLIRIPLTKFVRPLTEFRPTTPTSPSSDTQQQTGETQDSQPAGQNQ

>K6989_RS02430-348
MPKIVVDGYVADGAGANKAQHEDANKTKLQEWFKTSSNWEKLAE
QLTKKLGASKFKNVVLTAPTVSYEDDESNTRIPKVTFTLRGKDGYTLQTDNGASQSLT
LSIRVLYTTENQTENALKYQGASASASAVPNNTTTVNDENVSRTVNVYLNYTGPAIVL
DQVVPAVGTAANTLINGTSNVDGAFNTAFRGKLSKGLLFTNRYANPLLKSVINYVNKF
DPKYRAEFVTNSINGVTITKVQNTKELRPGTLDDLDKDNVFLQQIQGDTEAVYFAVTA
IASNNWLNTVLVRIPLTKFVRPLTEFQDQSATAPTQEGQTTQGQENSAGSGALGSTTG
AQTPSGGSSQASGS

>K6989_RS02440-321
MPKIVVQGYVADTTEDNKEEIKEANKTKLQNWFKAPANWEKLSE
QLTKKLGADKFKNVKLTYKSITLTDFTVAGATRWNPTVKFSIEANEGYTLDTNSTREI

DLVVRVLYKSAEANAANVFTHEGASPSAAPGNTKTPNDPKVIKNVNVYLNYTGPSIVL
DEALPTVGSQDNTSINGTSNVTSDFNNAFRGNPSSGLLFTDRYPNPLLQSVINYVNKF
DPKYRAAFVTDSKNGVTITKVQNTRELRPGTLDDLNGNNVFLQQMKNDTEAVYLPVTA
IASNGWLNTVLVRIPLTKFVRPVAPFAPAAPAQPQGGQTETSQPA

>K6989_RS02455-408
MADLASTVSYLKSLNDTLKATTDALNGDNPTTKTAYYKADTGRT
LYWDGFMPKIVTTLLEGETNYREYNPKPLTGSFIFVPNRNKRIIAQWLRNNPDKLALV
AEQLTRKLGKDKFKNMTLTNPVISMDELIRSEGRYYTPKVTFNLTPKDGYTLADNSEN
TVTLTIRNLYKEANPDTNVFATQGTSFSAVPSGSSVDDANVKANVNVYLNYTGPSIVL
DQDLPTVGTANNTSLNGTSNVTGDFNTKFKNLLINVVGESITQTSLFQAVINYVNKFD
PKFRAQFVTNSRDGVTITKVQSNTQLRPGTLDDLVKNDRDNVFLQQIQGDTEVVYLPV
TALTNDKWLNTVLVRIPLTKFVRPLTEFQDQSATAPTQEGQTTQGQENSAGSGALGST
TGAQTPSGGSSQASGS

>K6989_RS02460-238
MLYTSADATENALRYQGVSFSAAPNKAKEANHNQTIKDVDVYLN
YTGPNIELDAEAPTVGATPNTSINGTSNVKGTFNDKFKKLLVNVVGENITQTSLLQAI
INYVNKFDPKFRAEFVTSINVVTVASVQNNTQLRIGSLNDFLWNKKGFLQQVNGDSSA
VYFAVNGVTSQGWLNTFLIRIPLTKFVRPISVLEEQTTEEETQESQEGQENQEQGSTT
QTNTESSGTGQSSDLTEAAA

>K6989_RS02475-334
MPKIVIDDYVADGYHVANNTKNNRAQHEAANRPLLEQWFKVNQD
KLSLVAEQLTKKLGEEKFKNMTLSNPEISWDEVKFSNKVSNKGYLTPKVTFNLTPKED
YKKASDSSEKVTLTIRNLYKEANSDTNVFATQGAYYTATPSDNATTIKKVNVYLNYTG
PNIELDADLPRVGEQENTSLNGTSNVTDDFNNKFNKLLIRYKAETSLFQTIINYVNKF
DPKFRAAFVTSINGVAIASVQNGTQLRIGNLNDLLWNNKVFLQQVNGDSSAVYFAVNG
VTSEGWLNTFLIRIPLTKFVRPLTEFQAQSPAAPTEEASGDQAQADPQATQMGTQAQS

>K6989_RS02490-342
MPKIVVQGYEADGEEKGKATHKEANQKKLEDWFAANQDKLSLVA
DQLTKKLGEEKFKNMVLSTPTITWDEVEFSNKGSNKVYLTPKVTFNLAAKDGYALAEG
SAATVTLTIRVLYKDSNLTRNVLSTQGAYYTAAPKNAIVDDANVKAKVNVYMNYTGPN
IVLDADLPTVGEQENTSINGTSNVEGTFNTKFKNLLVNVLRRGHAESSLLQAIINYVN
KFDPKFPAEFVLKTNGVTVASVQNNTQLRIGSLNDFLYNNKGFLQQVNGDSTAVYFAV
MGVTSNGWVNTFLIRIPLTKFVRPISVLEEQSAQDQTPEASQQAGGDQSTSSSPQETA
QTQSNQGS

>K6989_RS02520-355
MPKIVVSLLTTKPANQEYDANPRTTSVTFWKQRNGEILDVWFAA
NQDKFTLIADQLTKKLGKDKFKNMILSNPRITWDELTIPEGTILTPKVTFNLTPKEGY
EKASDSSEKVTLTIRNLYKDSNPEVNVFQTQGSSPSTEPAGSTPSNNAAVIIDINVYM
NYTGPSIVLDEALPTVGGKENTSINGTSNVTGAFNNAFRGNPTKGLLFTNRYPNPLLQ

SVINYVNKFDPKFRAAFVTEKNGVALTSVQSGTQLKIGSLNDFLYNNKGFLQQVHGDS
TAVYFAVNGVTSQGWLNTFLIRIPLTKFVKPLTEFRPTTPTSPSSDTQQQVTEQTSGN
QDASGSASESGIASQPQQAAS

>K6989_RS02525-345
MPKIVTTALPTTVAPMNREYNESPTSGYEFWKTRNALILTTWFN
QNQDKLSLVAEQLTKKLGEEKFKNMTLSNPTISWDEVRFSKGNVTKLYLTPKVTFNLA
AKEGYALAQDSATSVTLTIRVLYKDSNPEVNVFQTQGSSPSAAPSGSSVDDANVKAKV
NVYLNYTGPSIVLDEALPTVGGKENTSINGTSNVDGAFNNAFRGNPTSGLLFTNRYPN
PLLKSVINYVNKFDPKYRATFVTNSRDGVTITKVEKTKELRPGTLDDLVKNRNIVFLQ
QMKNDTESVYLPVTALTNDKWLNTVLVRIPLTKFVRPISVFQATTAAPQDAAQASEGQ
GSQPDQTGQTA

>K6989_RS02530-332
MPKIVVPGYEADGEGKGKATHEEANKTKLEAWFASNQDKFTLVA
DQLTRKLGSDKFKNVTLTNPQVSYEEVTVNSNAWKNPKVTFNIQAKPGYQLTEPTTDS
KQISLTIRVLYENQASTQNLLTIQGASPTAAPNTASVGNPATAIKSVNVYLNYTGPAI
MLNADLPTVGGQENTLINGTSNVTGAFNNAFRGNPTKGLLFTNRYPNPLLQSVINYVN
KFDSKFSAKFVTNSRDGVTITKVEKTKELRPGTLDDLVKNRNIVFLQQIKGDTEAVYF
TVTALTNDKWLNIVLVRIPLTKFVRPLTEFQAQSATAPTQEGQTTQGQENLQVQVL

>K6989_RS02560-329
MPKIVVQGYEADGEGNGKATHKEANQKKLEDWFAANQDKLSLVA
DQLTKKLGADKFKNVTLTNPQVSYEEVTVNTYTWKNPKVTFNIQAKPGYQLTEPTTDS
KQISLTIRVLYENQTYTQNLLTIQGASPTAAPNTASVGNPATAIKSVNVYLNYTGPAI
MLNADLPTVGGQENTLINGTSNVTGEFNTKFRKLLVSVFRQGHAQSSLFQAVINYVNK
FDSKFSAKFVTNSRDVVTITKVQNTKELRPGTLDDLNNNNVFLQQIKGDTEAVYFTVT
ALTNDKWLNIVLVRIPLTKFVRPLTEFQAQSATAPTQEGQTTQGQENLQVQVL

3684
11,238-408

>K6989_RS02410-152
MPKIVLEAFDSTWRENSANQTKIREWFREASNWASLSDQLTKKL
GAERFKNVKLTFKNATFVGTNIKTPTVKFTVKAKEGYTLTTGDNASATEISLVVRVLY
NRGSESTIQMPTQGASSSAAPNTASADNPASAIKNVNVYLNYIKQFKLTN

>K6989_RS02450-157
MPKIVVQDYVADTTENDKKEANKTKNQEKLKAWFGDAANWVGLE
DQLTKRLGSDKFKNVKLTYKSITLTDFTVAEATRWNPTVKFNVKANEGYTLDTNSAKE
IDLVVRVLYNSAEANAANVFTHEGASSSAAPGNTKTPNNAEVIKNVNVYLNYTDI

>K6989_RS02470-147
MPKIVLTDFDKTWEQNSENERKIREWFDNATNWATLSEQLTKKL

GAERFKNVKLTYKEVTFSTNAVKTPTVTFTVAAKEGYTLVDSGENASATEISLVVRVL
YNRDNESTIQMPTQGASSSAAPRGATVNDANVKAKVNVYLNYTDI

>K6989_RS02495-124
MLAITKYIQSFDPKYLSTRTDNGYRAWVSWMWTNRSALSQRELK
QPASIFLQQMKDDSEAVYFAVMGVTSNGWVNTFLIRIPLTKFVKPVTVFIPPVVTPPA
EEATEGSQTQASTPQTMPNSSQ

>K6989_RS02515-155
MPKIVVDDYVADTAGNNRAEHEAANLEKLRTWFDTPANWEKLSE
QLTKKLGADKFKNVTLTNPVISYDDVSTGRDVRIPKVTFTVAAKDGYNLTEPTGETKQ
ITLSIRVLYTSQNQPENALRYQGVAFSAAPSGSSVGAANVKAKVNVYLNYTDI

>K6989_RS02540-147
MPKIVLDNFDSTWGRNSDNQNKIKAWFGDAANWVGLSDQLTKKL
GAERFKNVKLTFKNATFAGTNIKTPTVTFTVAAKEGYTLVDSGENASATEISLVVRVL
YNRDNESTIQMPTQGASSSAAPREATVNDAKVKAKVNVYLNYTGC

>K6989_RS02550-165
MPKIVTTALPTTVAPMNREYNESPTGGHEFWKGRNKQVLDAWFT
ANQDKLSLVAEQLTKKLGKDKFKNMVLSTPTITWDEVQFSNRLYLTPKVTFTLTPKEG
YEKVSDSSETVTLTIRNLYKEANPDTNVFATQGTFPSVAPSDSSVDDANVKAKVNVYL
NYTDI

>K6989_RS02565-124
MLAITKYIQSFDPKYLSTRTDNGYRAWVSWMWTNRSALSQRELK
QPASIFLQQMKDDSEAVYFAVMGVTSNGWVNTFLIRIPLTKFVKPVTVFIPPVVTPPA
EEATEGSQTQASTPQTMPNSSQ

>K6989_RS02580-125
MPKIVVDDYVADTAGNNRAEHEAANLEKLRTWFDTPANWEKLSE
QLTKKLGADKFKNVTLTNPVISYDDVSTGRDVRIPKVTFTVAAKDGYNLTEPTGKQNK
LLYQLEFYTQVKINQKTHLDIKV

>K6989_RS02585-165
MPKIVVDGYVADGEGNGKATHEATNQQKLQEWFATSSNWEKLSE
QLTKKLGADKFKNVTLTNPQVSYEEVTVNSNTWKNPKVTFTIEAKPGYELTQPTTDPK
QISLTIRVLYENQASTQNLLTIQGASPSAAPRNATVNDANVKAKVNVYLNYTGPNIEL
DAELP

1461
10,124-165

Mycoplasmopsis synoviae 53

>MS53_RS03930-358
MLIKFWDGFMPKIVIDDYVADGYDVANNAENNRAQHEAANRPLL
EQWFKVNQDKLSLVAEQLTKKLGEEKFKNMTLSNPTISWDEVEFSNKVSNKGYLTPKV
TFNLTPKEDYKKASDSSEKVTLTIRNLYKEANSDTNVFATQGAYYTATPSDNATTIKK
VNVYLNYTGPSIVLDADLPRVGEQENTSLNGTSNVTDDFNNKFNKLLIRYKAETSLFQ
TIINYVNKFDPKFPAKFVTNSINGVTITKVEKTKELRPGTLDDLLKNKNNVFLQQIKG
DKEAVYFAVTAIASNNWLNTVLVRIPLTKFVKPLSVFTAPVVTPPAEEATGGSQTQEA
RLQPAGYSNSDSFGSTEQTPPSTT

>MS53_RS04020-333
MLALYWDGFMPKIVLTDFDKTWGQNTANQDKIKAWFGDASNWAD
LSEQLTKKLGVDKFKNVVLSQPNVTFEIINWNGQTWRVPTVTFNLAAKEGYELTGSTN
TIALKIRVVYDSNDANAILFPVQGASSSATPNNASQANDATTIEKVNVYLNYTGPAIM
LNADLPTVGEQENTSINGTSNVDGDFNTKFKNLLVNVVGGSFTQTSLLQAIINYVNKF
DPKFPASFVLNTNGVALTSVQNNTQLRIGSLNDFLYNNKGFLQQVNGDSSAVYFAVNG
VTSQGWLNTFLIRIPLTKFVKPLIEFRPTTPTNTSSDTQQQDTQQQGTAQTQSNQGS

>MS53_RS04025-296
MLLLYWDGFMPRIVIERFDTTWTENTRNRNDLVAWFQNTANWSQ
VSEQLTKKLGVEKLRNVNLVYKNITWVNSPAGAQHKIPTVTFTVEARDGYTLADDSTR
ELSLVVRNLYNSSAVDATTLFPYQGASSSAVPSGSSVDDANVKANVNVYLNYTGSSIV
LYEALPTVGGKENTSLNGTSNITDDFNNKFNKLLIRYKAETSLLQAIINYVNKFDPKF
RAQFVTKTNGVALTSVQNGTQLKIGSLNDFLWNNKGFLQQVNGDSSAAYFAVNGVTSQ
GWLNTFLIRIPLNKFVKAIL

>MS53_RS01225-364
MLIKFWDGFMPKIVVQGYQADGYVADGQGNNRAAHEDANKTKLQ
NWFNAPANWEKLSEQLTKKLGADKFKNVTLSSPTVTYETISTGRDVRIPKVTFTVTAK
NGYNLTAPTGNTKQISLSIRVLYTSASSTENALRYQGVSFSATPKDASVENPATAIKN
VNVYLNYTGPSIVLDQAVPAVGTANNTSLNGTSNVDGDFNTKFKKLLVRVLNGGHAES
SLFQAVINYVNKFDPKFRAQFVTNSINGVTITKVESGTQLRPGTLDDLVKNDRNNVFL
QQMKNDTEAVYLPVTALTSDKWLNTVLVRIPLTKFVKPLTEFQAQSATAPTQEGQTTQ
GQENSAGSGALGSTTGTQTPSGGSSQASGS

>MS53_RS01300-338
MLALHWDGFMPKIVVEGYVEDIEENNKNTNQQKLETWFKANQDK
FTLVADQLTRKLGEEKFKNMTLSNPTISWDEVKFSNKVSNKGYLTPKVTFNLTPKEDY
KKASDSSEKVTLTIRNLYKEANSDTNVFATQGAYYTATPSDNATTIEKVNVYLNYTGP
NIELDADLPTAGNKNNTSINGTSNVTGDFNNAFRGNPSKGLLFTNRYANPLLKSVINY
VNKFDPKYRATFVTDSRDGVTITKVENTKELRPGTLDDLVKNRNKVFLQQIKGDKEAV
YFAVTAIASNNWLNIFLIRIPLTKFVRPLTEFQAQSPATPTQEASGDQAQASTPQTIP
NSSQ

>MS53_RS01355-231
MLALYWDGFMLKIVVDDGYVEDIEENNKENNKNTNQQKLEAWFK
ANQDKFTLVADQLTRKLGEEKFKNMTLSNPTISWDEVKFSNRGSNKGYLTPKVTFNLV
AKDGYTLAEESAATVTLTIRVLYKDSNLTRNVLSTQGAYYTATPSDNATTIKKVNVYL
NYTGPSIVLDQAVPAVGTANNTSLNGTSNVDNDFNNKFKKLLVSGTTQTPLLQTIINY
VNNLTLNFQLHLY

1920
6, 231-364

>MS53_RS03930-157
MLALYWDGFMPKIVLEGFDSRWTNVEDNKSKLQTWFNTKANWSN
LEDQLTKKLGAERFKNVKLTYKEVTFSGDDIKTPTVTFTVAAKEGYTLVDSGENASAT
EISLVVRVLYNRDNESTIQMPIQGASSSAAPGNTKTPNNATVIKNVNVYLNYTDI

>MS53_RS01220-169
MLALYWDGFMPKIVVDDGYVENTEENNKENNKNTNQQKLEAWFK
ANQDKFTLVADQLTRKLGEEKFKNMTLSNPTISWDEVKFSKGNVTKLYLTPKVTFNLA
AKEGYEKASDSSETVTLTIRNLYKEANPDTNVFATQGASSSAAPRGATVNDANVKAKV
NVYLNYTEI

>MS53_RS01330-169
MLLLYWDGFMPKIVVKDYQPAGHGNNKDTNENTNQQKLQNWFNT
SSNWEKLSEQLTKKLGADKFKNVVLTAPTVSYDDVSTAISTDSEVRIPKVTFTLQGKE
GYKLQTTDGASTTLSLSIRVLYTSENQTENALKYQGASSSAVPNRASSPNDETTIKTV
NVYLNYTDI

495
3,157-169

Mycoplasmopsis synoviae strain 86079/7NS

>DEH79_RS01165-358
MPKIVTTALPTTVAPMNREYNESPTGGHEFWKGRNKQVLDAWFA
ANQDKLSLVAEQLTKKLGEEKFKNMVLSNPTISWDEVKFSNRLYLTPKVTFNLAAKEE
YEKSSDSLETVTLTIRNLYKAAAPNTNLFATQGASSSAAPGNTMKPNDATTIEKVNVY
LNHTGPSIVLDAEFPQVGNQNNTSLNGTSNVTGAFNNAFRGLLFTKRLVNPLMQSVIN
YVNKFDPKFPAAFVTNATNGVTITKVQKDRQKKDRELRPGNLEDLLYNNNVFLQQMKN
DTEAVYFAVTAIASNNWLNTFLIKIPLTKFVRPISVLEEQTTEEETQEGQENQEQEST
AQTNTEFSGTAQSSPSTGTNSGSK

>DEH79_RS01180-319
MPKIVVKDYQPAGDGSNKEANKTTNQKKLEAWFGDAANWAGLED
QLTKRLGSDKFKNVKLTYKSITLTDFTVAGATRWNPTVKFSVKAKDDYALDSTSAREI

DIVVRVLYKSAEANAANVFTHEGASSSAAPENTKKPNDPKVIKNVNVYLNYTGPSIVL
DEAHPTVGATPNTSLNGTSNVTGEFNTKFRKLLVSVFRQGHAESSLFQAVINYVNKFD
PKFRAQFVTNSIDGVTITKVQSNTQLRPGTLDDLVKNDRDNVFLQQIQGDTEAVYLPV
TAIASNKWLNTFLIRIPLTKFVRPLTEFQAQSSPTTGTSSSSN

>DEH79_RS01205-358
MPKIVTTALPTTVAPMNREYNESPTGGHEFWKGRNKQVLDAWFA
ANQDKLSLVAEQLTKKLGEEKFKNMVLSNPTISWDEVKFSNRLYLTPKVTFNLAAKEE
YEKSSDSLETVTLTIRNLYKAAAPNTNLFATQGASSSAAPGNTMKPNDATTIEKVNVY
LNHTGPSIVLDAEFPQVGNQNNTSLNGTSNVTGAFNNAFRGLLFTKRLVNPLMQSVIN
YVNKFDPKFPAAFVTNATNGVTITKVQKDRQKKDRELRPGNLEDLLYNNNVFLQQMKN
DTEAVYFAVTAIASNNWLNTFLIKIPLTKFVRPISVLEEQTTEEETQEGQENQEQEST
AQTNTEFSGTAQSSPSTGTNSGSK

>DEH79_RS01220-306
MPKIVLTDFDKTWEQNSENERKIRKWFDNAVNWAGLSDQLTKKL
GAERFKNVKLTYKEVTFSTNAVKTPTVKFTVAAKEGYTLDNSANEISLVVRVLYNRDN
ESTIQLPTQGASSSAVPNRASSPNDETTIKTVNVYLNYTGPSIVLDEAHPTVGEQENT
SINGTSNVTGEFNTKFRKLLVSVFRQGHAESSLFQAAINYVNKFDPKFRAEFVTDARD
GVTITKVQSNTQLRPGTLDDLVKNDRDNVFLQQIQGDTEAVYLPVTAIASNKWLNTFL
IRIPLTKFVRPLTEFQAQSSPTTGTSSSSN

>DEH79_RS01250-331
MPKIVVDDYVADTTENNKEEIKEANKTKLQNWFKAPANWEKLSE
QLTKKLGVDKFKNVKLTYKSITSTDFTAGRGATRWNPTVKFSVKANEGYTLDINSTRE
IDLVVRVLYKSAEANTDNVFTHEGASSSAAPGNTKKPNDPKVIKNVNVYLNYTGPSIV
LDEALPTVGRQENTSINGTSNVTGEFNNAFRGNPSSGLLFTHRYANPLLKSVINYVNK
FDPKYRATFVTNSIDGVTITNVTDKVQLRPGTLDDLKGNNVFLQQIKGDTEAVYFAVT
AIASNKWLNTFLIRIPLTKFVRPISVFQATTAAPQDAAQASEGQGSQPDQTGQTA

>DEH79_RS01255-320
MPKIVVNGYVADTTGDNEAAHEEANKTKLQNWFNAPANWEKLSE
QLTKKLGADKFKNVTLSSPTVTYETISTGRDVRIPKVTFTVTAKNGYNLTAPTGNTKQ
ISLLIRVLYTSASSTENALRYQGVSFSATPKDTSVENPATAIKNVNVYLNYTGPSIVL
DQAVPAVGTANNTSLNGTSNVTGAFNNAFRGNPSRGLLFTNRYPNPLLQSVINYVNKF
DPKFPAVFVTNATNGVTITKVESDTQLRIGTLDDLVKNRNNVFLQQIQGDTEAVYLPV
TAIASNNWLNTVLVRIPLTKFVRSISVLEEQSAQDQTPEASQQS

>DEH79_RS01260-348
MPKIVVDGYVADGEENNKENNKNTNQQKLEAWFTANQDKFTLVA
DQLTRKLGKEKFKNMTLSNPRITWDEVRFSKGNVTKLYLTPKVTFNLAAKEEYEKSSD
SSEKVTLTIRNLYKEANPDTNVFATQGAYYTATPSDNATTIEKVNVYLNYTGPAIMLN
ADLPTVGGQDNTSINGTSNVDDAFNTAFRGNPSSGLLFTNRYANPLLQSVINYVNKFD
PKFRAEFVTNSINGVTITKVEKTKELRPGTLDDLLKNRNNVFLQQMKNDTEAVYLPVT

ALTNDKWLNTVLVRIPLTKFVKPLSVFTAPVVTPPAEEATGGNQTQEARLQPAGYSNS
DSSGSTEQTPPSTT

>DEH79_RS01275-328
MPKIVVDDGYVADTNEGSKEADKTTNQQKLQEWFNTSSNLEKLA
EQLTKKLGEDKFKNMVLSNPTITWDEVQFSNRLYLTPKVTFNLAAKEGYEKATGSLET
VTLTIRNLYKAADPNTNLFATQGASSSAAPGNTMKPNDAAVIKNINVYLNYTGPSIVL
DQAVPAVGTANNTSLNGTSNVTGAFNNAFRGKPSSGLLFTNRYPNPLLQSVINYVNKF
DPKYRVAFVTNSIDGVTITKVESGTQLRPGTLDDLNKNNVFLQQIQGDTEAVYFAVTA
IASNNWLNTFLIRIPLTKFVKPLTEFRPTTPTSTSSDTQQQGTAQTQSNQGS

>DEH79_RS01280-396
MGEDSAAEGAQEEDAQAPQAPTTPDLASTVSYLKSLNDTLKAAT
DALNGDNPTTKTAYYKADAGRTLYWDGFMPKITLESFTASQTNVEDNKSKLQTWFNTK
ANWSNLEDQLVKKLGSEKFKNVKLTNPQVDWDIVNSNQYRPKVTFDVTQKEGYELQSG
SEFSQSLTMVIRVLYTTQSPNANVLQMQGASASAAPAGSTPSNNAAVIRDVNVYMNYT
GPNIELDAELPQVGNQNNTSINGTSNVDGDFNTKFKNLLVNVVGGNFTQTSLLQAIIN
YVNKFDPKFRAAFVTEKNGVALTSVQSGTQLRIGSLNDFLYNNKGFLQQVNGDSSAVY
FAVNGVTSEGWLNTFLIRIPLTKFVKPLSVFTAPVVTLPAEEATEGSQTQTSTPQTMP
NSSQ

>DEH79_RS01285-367
MPKIVVEDYQPAGHGNNKDTNETTNQQKLQNWFNISSNWEKLSE
QLTKKLGADKFKNVTLSSPTVTYETISTGRDVRIPKVTFTVAAKDGYNLTKPTGETKQ
ISLSIRVLYTSQNQTENALRYQGVSFSAAPNGANSVNHAQTIKDVNVYLNYTGPSIVL
DEALPTVGTTPNTSINGTSNVEGTFNDKFKKLLVNVVRGGHAESSLFQAVINYVNKFD
PKFRAVFVTNSRDGVTITKVQSNTQLRPGTLDDLVKNDRNNVFLQQIQGDTEAVYLPV
TAIASNNWLNTVLVRIPLTKFVRPISVLQTPDQQQTQEGGEEATQPQDAGSGTTSTSE
AQSGQSNGPAGSGTGSTQQAQSSPKTGTSSSSN

>DEH79_RS01290-327
MPKIVTTALPTTVAPMNREYNESPTSGYEFWKTRNALILTTWFG
QNQDKLSLVADQLTKKLGEEKFKNMTLSNPRITCDEVKFSNKGSNEDYLTPKVTFNLA
AKKGYALAQDSATSVTLTIRVLYKDSNPEVNVFQTQGSSPSAAPKNTKTPNNAEVIKN
VNVYLNYTGPNIELNAEFPKVGSQENTSLNGTSNVTDDFNNKFNKLLIRYKAETSLFQ
TIINYVNKFDPKFPAAFVTNATNGVTITKVESGTQHRPGTLDDLDKNNVFLQQIQGDT
KAVYFAVTPIASNNWLNTVLVRIPLTKFVRPISVLEEQSAQDQTPEALQQS

>DEH79_RS03645-378
MPKIVVNGYVADTTGDNEAAHEEANKTNQKKLEAWFTNSANWAG
LEDQLTKKLGADKFKNVVLTTPTVSYEKVTVGKYTWKNPKVTFNIQAKPGYQLTEPTT
NPKQISLTIRVLYSSQESNQNLLTIKGAYPTAAPRNATVNDANVKSKVNVYVNYTGPA
IELNSDIPTVGAAQNTTINGTSKVEDSTLNNAVKALLSGNTTTDNVSRPLMDAITKYI
QTFDPKFKTNVTPSTYTNTFRAWTSWMETRQREMQGNVALVPALVQVELKMPDNLFLQ

QMKNDTEAVYFAVTAIASNGWLNTFLIRIPLTKFVKPLTVFTATPASASTQDSSASNT
SPQTQLKVLNNKTKNMPQNVSAILRRVYAENCNYSSTYYCCTNE

>DEH79_RS01310-375
MPKIVTTLLEGETNYREYNPNPLTGSFIFVPNRNKRIIAQWLRN
NPDKLALVAEQLTRKLGKDKFKNMTLTNPVISMDELIRSEGRYYTPKVTFNLTPKDGY
TLADNSENTVTLTIRNLYKDSNPEVNVFQTQGSSPSAAPSGSSVDDANVKAKVNVYLN
YTGPSIVLDQNLPTVGTANNTSLNGTSNVTGDFNTKFKKLLVRVLNGGHAESSLFQAV
INYVNKFDPKFRAVFVTNSRDGVTITKVQSNTQLRPGTLDDLVKNDRNNVFLQQIQGD
TEAVYLPVTAITSNNWLNTVLVRIPLTKFVRPISVLQTPDQQQTQEGGEEATQPQDAG
SGTTSTSEAQSGQSNGPAGSGTGSTQQAQSSPKTGTSSSSN

>DEH79_RS01335-366
MPKIVVQGYQADGEGNDKATHEEANKTKLQNWFNAPANWEKLSE
QLTKKLGADKFKNVNLTNPVISYDDVSTGRDVRIPKVTFTVTAKDGYNLIAPEGDAKQ
ISLSIRVLYTSQNQTENALRYQGVSFSAAPSGSSVDDANVKAKVNVYLNYTGPSIVLD
QNLPTVGTANNTSLNGTSNVTGDFNTKFKKLLVRVLNGGHAESSLFQAVINYVNKFDP
KFRAVFVTNSRDGVTITKVQSNTQLRPGTLDDLVKNDRNNVFLQQIQGDTEAVYLPVT
AITSNNWLNTVLVRIPLTKFVRPISVLQTPDQQQTQEGGEEATQPQDAGSGTTSTSEA
QSGQSNGPAGSGTGSTQQAQSSPSTGTSSSSN

>DEH79_RS01350-345
MPKIVVQGYEADGEEKGKATHKEANQKKLEDWFAANQDKLSLLA
DQLTRKLGKDKFKNMVLSTPTITWDEVEFSNKGSNKVYLTPKVTFNLAAKDGYALAEG
STTTVTLTIRVLYKDSNLTRNVLSTQGAYYTAAPKNAIVDDANVKAKVNVYLNYIGPS
IVLDEALPTVGEQENTSINGTSNVTGDFNTKFKKLLVRDNAQTSLLQAIINYVNKFDP
KFRAAFVTSINGVTVASVQNNTQLRIGSLNDFLWNKKGFLQQVNTDTKAVYFAVMGVT
SNGWVNTFLIRIPLTKFVRPISVLEEQTTEEETQESQEGQENQEQGSTTQTNTESSGT
GQSSDLTEAAA

5222
15, 306-395

>DEH79_RS01155-155
MPKIVVQGYVADTTGSSKDTNENANRPLLQQWFDTPANWEKLSE
QLTKKLGYDKFKNVTLTNPVISYDDVSTGRDVRIPKVTFTVAAKDGYNLTEPTGETKQ
ITLSIRVLYTSQNQTENALRYQGVAFSAAPSGSSVDDVNVKAKVNVYLNYTDI

>DEH79_RS01175-146
MPKIVLEGFDSRWTQNSENQRKIREWFREASNWAGLSDQLTKKL
GAERFKNVKLTFKEASFSGDDIKTPTVKFTVKAKEGYTLTTGDNASATEISLVVRVLY
NRGSENVIQLPRQGSSPSAAPSNSSVDDANVKAKVNVYLNYTDI

>DEH79_RS01195-155

MPKIVVQGYVADTTGSSKDTNENANRPLLQQWFDTPANWEKLSE
QLTKKLGYDKFKNVTLTNPVISYDDVSTGRDVRIPKVTFTVAAKDGYNLTEPTGETKQ
ITLSIRVLYTSQNQTENALRYQGVAFSAAPSGSSVDDVNVKAKVNVYLNYTDI

>DEH79_RS01215-146
MPKIVLEGFDSRWTQNSENQRKIREWFREASNWAGLSDQLTKKL
GAERFKNVKLTFKEASFSGDDIKTPTVKFTVKAKEGYTLTTGDNASATKISLVVRVLY
NRGSENVIQLPRQGSSPSAAPSNSSVDDANVKAKVNVYLNYTDI

>DEH79_RS01230-142
MPKIVLEGFDSTWGRNSDNQDKIRAWFGDAANWAGLSDQLTKKL
GAERFKNVKLTFKEASFSTNAVKTPTVKFTVEAKDGYTLDNSANEISLVIRVLYNRGS
ENVIQLPTQGASPSAAPSNSSVDDANVKAKVNVYLNYTDI

>DEH79_RS01270-148
MPKIVLEGFDSSWTNDLDNQTKIREWFDNAANWAGLSDQLTKKL
GAERFKNVKLTFKNATFVGTNIKTPTVTFTVAAKEGYTLVDSGENASATEISLVVRIL
YNRGSESTIQMPTQGASSSAAPGNPTEPNNATVIKNVNVYLNYTDI

>DEH79_RS03715-136
MPKIVLDNFDSSWTNANDSANQNKIKAWFREASNWASLSDQLTK
KLGAERFKNVKLTFKNATFVGTNIKTPTVTFTVAAKEGYTLAKGQAASAQEISLVVRV
LYNRDDESTIQLPTQGASSSAVLIRLQKLTMKMF

1028
7,136-155

Mycoplasmopsis synoviae strain A4

>K6990_RS01685-334
MPKIVVEGYVADTTGSSKDTNETANKTKLQTWFDTKANWAGLED
QLTKKLGADKFKNVVLTSPMVSYEEITVNSNTWKNPKVTFTVEAKPGYELTQPTTDPK
QISLTIRVLYENQTSTQNLLTIQGASPTAAPSNSSVDDANVKAKVNVYLNYTGPAIVL
DQDLPTVGTANNTSLNGTSNVEGTFNTKFKNLLVNVVGGSFTQTSLLQAIINYVNKFD
PKFRAAFVTSINGVALTSVQSGTQLKIGSLNDFLYNNKGFLQQVNGDSSAVYFAVNGV
TNKGWLNTFLIRIPLAKFVKPLTEFRPTTPTSPSSDTQQQGTEQTGETQGSQPAGQSQ

>K6990_RS01690-343
MPKIVVDGYVAAGYEADSAGTNKAQHETANQEKLQQWFEANQDK
LSLVAEQLTKKLGEEKFKNMTLSNPTISWDEVKFSNKGSNKGYLTPKVTFNLAAKDGY
TLAKESANTVTLTIRVLYKDSNLTRNVLSTQGASSSAAPAGSTPSNNAAVIRDVNVYM
NYTGPAIVLDQAVPAVVTAANTLINGTSNVDGAFNTAFRGLLFTTRYANPLLKSVINY
VNRFDPKYRAEFVTNATNGVALTKVQKDRQKKDRELRPGNLEDLLYNDNVFLQQMKND
TEAVYFAVTALTNDKWLNTVLVRIPLTKFVKPLSVFTAPVVTPPAEEDTEGSQTQTST

PQTMPNSSQ

>K6990_RS01705-336
MPKIVNPALPEEVAPRNREYNESPNGGYPFWKERNALILTTWFN
QNQDKLSLVADQLTKKLGEEKFKNMTLSNPRITWDELVRTEGTILTPKVTFNLAAKDG
YTLAEGSATTVTLTIRVLYKDSNPTRNVLSTQGASYSSAPRGATVNDANVKAKVNVYM
NYTGPSIVLDEAHPTVGATPNTSINGTSNVDGDFNTKFKNLLVNVVGGSFTQTSLLQA
IINYVNKFDPKFRAAFVLNTNGVAITKVENTKELRPGTLDDLLSNKDIVFLQQIKGDT
KAVYFAVTAIASNNWLNTFLIRIPLTKFVRPLTAFTATPASAPTQTDSGTGAQTQPEA
QS

>K6990_RS01725-468
MQFTSGSVTAAKAVKEDAEYSKVTDTTHKTTLEEKYTAAADFLT
EDSKLANLDASSNLDTTKATLESAKTALDAAVAAVKPDLDFQKTKTSAAAKVTELESL
VNTALKAELQRQVNELTKEQAAQATTMLENLTSLKDSLTSLQDLVSKGLEMQVDYPRN
YYDADNKTAFDDALLKASSVFPAFKWTAQSIMVPAPEEGALPNPRAWTKAREKSEFVL
QNFVMAPAPAPAPAQAAPATQSDSTAAAAASATVRLATGTEEAVEDSESSETQTPTPA
ADLASTVSYLKSLSDSLKAETDKLNGDTTENKTAYYKADTGRTLYWDGFMPKIVLTDF
DKTWEQNSEENERKIRKWFDNAANWVGLSDQLTKKLGAERFKNVKLTFKEASFSSDNI
KTPTVTFTVEAQDGYTLDNSANEISLVVRVLYNRGSESTIQLPTQGASSSAAPRGATV
NDANVKAKVNVYLNYTDI

>K6990_RS01785-327
MPKIVVDDGYVENTEENNKENNKNTNQQKLEAWFAANQDKFTLV
ADQLTRKLGEEKFKNMTLSNPRITWDEVEFSNKGSNKLYLTPKVTFNLAAKGGYGKAT
DSLEKVTLTIRNLYKEANPDTNLFATQGASSSAAPSGSSVDDAKVKANVNVYLNYTGP
SIVLDKAIPAVGTANNTSINGTSNVEGTFNDKFKELLVDNNLVPREFLSTVINYVNKF
DPKFRAQLVTDINGVAITRVQSKKELRIGNLNDLLYNKNVFLQQVHGDSSAVYFAVNG
VTSEGWLNTFLIRIPLTKFVRPLTAFTATPASAPTQTDSGTGAQTQPEAQS

>K6990_RS01790-328
MPKIVVEDYVSDGDGNNRAQHEAANQQKLQEWFNTSSNWEKLSE
QLTKKLGADKFKNVTLTNPQVSYEEVTVNSNAWKNPKVTFNIQAKPGYQLTEPTTEES
QISLSIRVLYSSQESNQNLLTIQGASPTAAPNNTTMVSDPNVLRTVNVYLNYTGPNIE
LDAELPQVGNQNNTSINGTSNVDGAFNTAFRGNPSRGLLFTHRYANPLLKSVINYVNK
FDPKFRAEFVTDSKNGVTISNVTNNVQLRPGTLDDLNGNNVFLQQIQGDTKAVYFAVT
AIASNGWLNTFLIRIPLTKFVRPLTEFQAQSEAAPTEETSGEQDSAPTGTGS

>K6990_RS03665-328
MPKIVLNNFDKTWGQNTTNQDKIKDWFREASNWAGLSEQLTKKL
GVDKFKNVVLSQPNVTFEIINWNGQTWRVPTVTFNLAAKEGYELTGSTNTIALKIRVV
YDSNDANAILFPIQGASSSAVPNRASSPNDEKTIKTVNVYLNYTGPNIELDAELPKVG
SQENTSLNGTSNVTDDFNNKFNKLLIRYKTETSLFQTIINYVNKFDPKFPAKFVTNSI
NGVTITKVEKIKELRPGTLDDLLKNRNNVFLQQIKGDKEAVYFAVTAVTIDKWLNTVL

VRIPLTKFVRPISVLEEQSAQDQTPEASQQAPEGGSLASGTAPTEGAQS

>K6990_RS01800-339
MPKIVVPTYEPDTTADNKAAHEETNKTKLEAWFGDAANWVGLED
QLTKRLGSDKFKNVKLTYASVTLTDFTAGRGVTRWNPTVKFRVEANEGYTLDTNSTRE
IDLVVRVLYKSAKSNAANVFTHEGASSSAAPGNTKTPNDPKVIKNVNVYLNYTGPSIV
LDEALPTVGATPNTSINGTSNVTGAFNTAFRGNPSKGLLFTSRYPNPLLQSVINYYVNK
FDPKFRAEFVTDSKNGVTITKVEKIKELRPGTLDDLNNNNVFLQQIQGDTKAIYFAVT
AIASNNWLNTFLIRIPLTKFVKPVTAFMPAAPTASVPTQDSSASNTIHKQQLKVHNNK
TKNMP

>K6990_RS01805-331
MPKIVVRLLTTKPANQEYDASVRQGGVVFRKTRNGEILDVWFAA
NQDKFTLVADQLTRKLGKDKFKNMTLSNPRITWDELTIPEGTILTPKVTFTLTPKEGY
EKASDSSETVTLTIRNLYKDSNPEVNVFQTQGSLPSAAPRGATVNDPNVKSKVNVYLN
YTGPSIVLDEALPTVGATPNTSINGTSNVEGTFNNAFRGNPSRGLLFTSRYPNPLLQS
VINYVNKFDPKYRAEFVTNSIDGVTITKVLKDKKQKDREELRPGTLDDLLSNKNKVFL
QQIKGDTEAVYFEVTAIASNNWLNTFLIRIPLTKFVRPISVFQATTEAPQDAAQN

>K6990_RS01810-311
MPKIVLEGFDSRWTNVNDNKSKLQTWFNTKANWSNLEDQLVKKL
GSEKFKNVVLSQPNVTLETFTWNRQTWGVPTVTFNLAAKEGYEKAKNSTNTIALKIRV
VYNSNNTNEILFPIQGASSSAAPSGANTADHTKTIKDVNVYLNYTGPAIILDATLPEV
GKVDNTTTNGTSNVTDDLNTKFKDLVTSDQRTASLFFQAIQKYINTFDPKYPANPISD
QKNGVTWARVENKALKIANLNNPNAVFVQQMKDDSEAVYLPVIGIANNRWLNTFLIRI
PLTKFVKPLTVFTATPASTPTQDSSASNTSPQTTT

3445
10,327-468

>K6990_RS01695-131
MPKIVVEGYVADTTGSSKDTNETANKTKLEAWFAANQDKFTLVA
DQLTRKLGSDKFKNVTLTNPIVTYEEITVNSNTWKNPKVTFNIQAKPGYQLTEPTKDP
KQISLTIRVLYKIRLLLKTCLLFKELLLL

>K6990_RS01735-162
MPKIVVEDYVSDGDGNNRAQHEAANQQKLQEWFNTSSNWEKLSE
QLTKKLGADKFKNVTLTNPQVSYEEVTVNTYTWKNPKVTFSIQAKPGYQLTEPTTNPK
QISITIRVLYLSQESNQNLLTIKGAFPSAAPNTASGDNPASVIKNVNVYLNYINQFKL
TN

>K6990_RS01750-187
MPKIVVEDYQPTSHGRNKENDETTNQNQEKLQNWFNAHANWEKL
SEQLTKKLGYDKFKNVTLTNPVISYDDVSTGRDVRIPKVTFTVAAKDGYNLTEPTGEK

KQITLSIRVLYTSQNQTENALRYQGVSFSAVPNRASNPNDANVMRNVNVYLNYTGPAI
VLDATLPEVGALIIQPLMVLLQLQMKL

>K6990_RS01760-135
MPKIVLEGFDKTWEQNSEENERKIREWFREASNWVGLSDQLTKK
LGAERFKNVKLTYKEVTFSTNAVKTPTVTFTVEAQDGYTLDNSANEISLVVRVLYNRG
SENVIQLPTQGASSSAAPGNPTEPNNATVIKKR

>K6990_RS01775-171
MADLASTASYLKSLSDSLKAETDKLNGDTTENKTAYYKADTTRT
LYWDGFMPKIVVEDYQSAGHGNNKDTNETANKTKLQNWFNTSSNWEKLAEQLTKKLGA
DKFKNVTLSSPTVTYETISTGSEVRIPKVTFTLQGKEGYELQTTDGASATLSLSIRVL
YTSENQTEKRT

786
5, 131-187

Mycoplasmopsis synoviae strain BS4S2

>K6986_RS00585-334
MPKIVVQGYEADGEEKGKATHKEANKTKLQEWFATSSNWEKLSE
QLTKKLGSDKFKNVTLSSPTVTYETISTGRDVRIPKVTFTVAAKDGYNLTVPTGNTKQ
ISLSIRVLYTSENSTQNALRYQGASSSAATSNSSVDDANVKAKVNVYLNYTGPEIMLN
AELPQVGNKNNTSINGTSNVDGDFNTKFKKLLVNVVSRGHAESSLFQAVINYVNKFDP
KFRAQFVTNSINGVTITKVQNTKELRPGTLDDLVKNDRNNVFLQQMKNDTEAVYLPVT
ALTNDKWLNTVLVRIPLTKFVRPLTEFQAQSPAAPTQEGQTTQGQENSAGSGALGSTT

>K6986_RS00590-332
MPKIVVDGYVADGYDVANNAENNRAQHEAANRPLLEQWFKVNQD
KLSLVAEQLTKKLGEEKFKNMVLSTPTISWDEVKFSNRLYLTPKVTFNLAAKEEYEKS
SDSLETVTLTIRNLYKAADPNTNLFATQGASSSAVPNRASSPNDETTIKTVNVYLNYT
GPNIELNAELPKVGFQENTSLNGTSNVTDDFNNKFNKLLIRYKAETSLFQTIINYVNK
FDPKYRATFVTDSKNGVTISNVTDKVQLRHGTLDDLNKNNVFLQQIKGDTEAVYFAVT
AIASNNWLNTFLIRIPLTKFVRPLTEFRPTTPTSPSSDTQQQTGETQDSQPAGQNQ

>K6986_RS00605-383
MQTAPAAETPAADLASTASYLKSLSDSLKAETDKLNGDTTENKT
AYYKTDTTRTLYWDGFMPKIVVPGYEAVDAGANKAQHETENTRLLQQWFDTPANWEKL
SEQLTKKLGSDKFKNVVLTAPTVSYEKVTVGRYTWKNPKVTFNIQAKPGYQLTEPTTN
PKQISLTIRVLYENQTSTQNLLTIQGASPSAVPSDANAENTSPTVKNVNVYLNYTGPN
IELDAELPTVGSQDNTSINGTSNVTGAFNTAFRGLLFTKRLVNPLMQSVINYVNKFDP
KYRAEFVTDSKNGVTITKVQKNRELRPGTLDDLNNNNVFLQQIKGDTEAVYFAVTAIA
SNNWLNTFLIRIPLTKFVKPLTVFTAPPASAPTQTDSGTGAQTQPEAQS

>K6986_RS00645-350
MPKIVVKDYQPAGHGNNKDTNETANKTKLQEWFATSSNWEKLSE
QLTKKLGSDKFKNVTLTNPQVSYEEVTVNTYTWKNPKVTFNIQAKPGYQLTEPTTNPK
QISLTIRVLYSSQESNQNLLTIKGASPSEAPSNSSVDDANVKAKVNVYMNYTGPDIVL
DQAVPAVGTANNTSLNGTSNVTGEFNTKFKKLLVNVVSRGHAESSLFQAVINYVNKFD
PKFRAQFVTNSINGVTITKVQNTKELRPGTLDDLVKNDRDNVFLQQIQGDTEVVYLPV
TALTNDKWLNTVLVRIPLTKFVRPLTEFQDQSATAPTQEGQTTQGQENSAGSGALGST
TGAQTPSGGSSQASGS

>K6986_RS00650-348
MPKIVVDGYVADGAGANKAQHEDANKTKLQEWFKTSSNWEKLAE
QLTKKLGASKFKNVVLTAPTVSYEDDESNTRIPKVTFTLRGKDGYTLQTDNGASQSLT
LSIRVLYTTENQTENALKYQGASASASAVPNNTTTVNDENVSRTVNVYLNYTGPAIVL
DQVVPAVGTAANTLINGTSNVDGAFNTAFRGKLSKGLLFTNRYANPLLKSVINYVNKF
DPKYRAEFVTNSINGVTITKVQNTKELRPGTLDDLDKDNVFLQQIQGDTEAVYFAVTA
IASNNWLNTVLVRIPLTKFVRPLTEFQDQSATAPTQEGQTTQGQENSAGSGALGSTTG
AQTPSGGSSQASGS

>K6986_RS00660-321
MPKIVVQGYVADTTEDNKEEIKEANKTKLQNWFKAPANWEKLSE
QLTKKLGADKFKNVKLTYKSITLTDFTVAGATRWNPTVKFSVEANEGYTLDTNSTREI
DLVVRVLYKSAEANAANVFTHEGASPSAAPGNTKTPNDPKVIKNVNVYLNYTGPSIVL
DEALPTVGSQDNTSINGTSNVTGDFNNAFRGNPSSGLLFTDRYPNPLLQSVINYVNKF
DPKYRAAFVTDSKNGVTITKVQNTRELRPGTLDDLNGNNVFLQQMKNDTEAVYLPVTA
IASNGWLNTVLVRIPLTKFVRPVAPFAPAAPAQPQGGQTETSQPA

>K6986_RS00680-353
MPKIVVPTYEPDTTDDNKAAHEEANKTKLEAWFTNSANWAGLED
QLTKRLGSDKFKNVKLTYKSITLTDFTVDGATRWNPTVKFSVKAKDDYALDSTSASEI
DLVVRVLYKSAEANAANVFTHEGASSSAAPKNTKTPNDPKVIKNVNVYLNYTGPSIVL
DEALPTVGSQDNTSINGTSNVTGDFNNAFRGNPSSGLLFTDRYPNPLLKSVINYVNKF
DPKFRATFVTDSKNGVTISNVTDKVQLRSGTLDDLLSNKNKVFLQQIKGDTEAVYFAV
TAIASNNWLNTVLVRIPLTKFVRPVAPFAAAAPAEPNSDTQEQAGEIGGSQDGSGSAS
KSGTEGQSSPSTGTSSSSN

>K6986_RS00685-416
MADLASTVSYLKSLNDTLKATTDALNGDNPTTKTAYYKADTGRT
LYWDGFMPKIVTTLLEGETNYREYNPKPLTGSFIFVPNRNKRIIAQWLRNNPDKLALV
AEQLTRKLGKDKFKNMTLTNPVISMDELIRSEGRYYTPKVTFNLTPKDGYTLADNSEN
TVTLTIRNLYKEANPDTNVFATQGTSFSAVPSGSSVDDANVKAKVNVYLNYTGPSIVL
DQDLPTVGTANNTSINGTSNVTGDFNTKFKNLLINVVGESITQTSLLQAIINYVNKFD
PKFPASFVLNTNGVALTSVQSGTQLRIGSLNDFLYNNKGFLQQVHGDSTAVYFAVNGV
TSQGWLNTFLIRIPLTKFVKPVTVFTPPVVTPPKQDDSQEDSSNAGNTNENTETPVED

QTGQESSESGSAQTDQAQQESPQT

>K6986_RS00690-238
MLYTSADATENALRYQGVSFSAAPNKAKEANHNQTIKDVDVYLN
YTGPNIELDAEALTVGATPNTSINGTSNVKGTFNDKFKKLLVNVVGENITQTSLLQAI
INYVNKFDPKFRAEFVTSINVVTVASVQNNTQLRIGSLNDFLWNKKGFLQQVNGDSSA
VYFAVNGVTSQGWLNTFLIRIPLTKFVRPISVLEEQTTEEETQESQEGQENQEQGSTT
QTNTESSGTGQSSDLTEAAA

>K6986_RS00695-330
MPKIVTTALPTTVAPMNREYNESPTSGYEFWKTRNALILTTWFN
QNQDKLSLVAEQLTKKLGEEKFKNMTLSNPTISWDEVRFSKGNVTKLYLTPKVTFNLA
AKEGYALAQDSATSVTLTIRVLYKDSNPEVNVFQTQGSSPSAAPSGSSVDDANVKAKV
NVYLNYTGPSIVLDEALPTVGGKENTSINGTSNVDGAFNNAFRGNPTSGLLFTNRYPN
PLLKSVINYVNKFDPKYRATFVTNSRDGVTITKVQNTKELRPGTLDDLNGNNVFLQQM
KNDTEAVYLPVTAIASNGWLNTFLIRIPLTKFVKPLTEFQAQSSPTTGTSLSSN

>K6986_RS00700-329
MPKIVVPGYEADGEGKGKATHEEANKTKLEAWFASNQDKFTLVA
DQLTRKLGSDKFKNVTLTNPQVSYEEVTVNSNAWKNPKVTFNIQAKPGYQLTEPTTDS
KQISLTIRVLYENQASTQNLLTIQGASPTAAPNTASVGNPATAIKSVNVYLNYTGPAI
MLNADLPTVGGQENTLINGTSNVTGEFNTKFRKLLVSVFRQGHAQSSLFQAVINYVNK
FDSKFSAKFVTNSRDGVTITKVQNTKELRPGTLDDLNNNNVFLQQIKGDTEAVYFTVT
ALTNDKWLNIVLVRIPLTKFVRPLTEFQAQSATAPTQEGQTTQGQENLQVQVL

3734
11，238-416

>K6986_RS00625-152
MPKIVLEAFDSTWRENSANQTKIREWFREASNWASLSDQLTKKL
GAERFKNVKLTFKNATFVGTNIKTPTVKFTVKAKEGYTLTTGDNASATEISLVVRVLY
NRGSESTIQMPTQGASSSAAPNTASADNPASAIKNVNVYLNYIKQFKLTN

>K6986_RS00670-157
MPKIVVQDYVADTTENDKKEANKTKNQEKLKAWFGDAANWVGLE
DQLTKRLGSDKFKNVKLTYKSITLTDFTVAEATRWNPTVKFNVKANEGYTLDTNSAKE
IDLVVRVLYNSAEANAANVFTHEGASSSAAPGNTKTPNNAEVIKNVNVYLNYTDI

>K6986_RS00710-165
MPKIVTTALPTTVAPMNREYNESPTGGHEFWKGRNKQVLDAWFT
ANQDKLSLVAEQLTKKLGKDKFKNMVLSTPTITWDEVQFSNRLYLTPKVTFTLTPKEG
YEKVSDSSETVTLTIRNLYKEANPDTNVFATQGTFPSVAPSDSSVDDANVKAKVNVYL
NYTDI

>K6986_RS00720-159
MPKIVVQGYEADGAGNGKATNEAANQQKLQQWFDTPANWEKLSE
QLTKKLGADKFKNVTLSSPKVTYETISTGKNVKIPKVTFTLQGKEGYNLQIDNGASQS
LTLLIRVLYTSADATENALRYQGVSFSAAPNKAKEANHNQTIKDVNVYLNYPGQILN

633
4,152-165

Mycoplasmopsis synoviae strain FJ-01

>KXD88_RS01055-384
MSEEVAAEAETQTPAPTPAADLASTASYLKSLNDTLKAETDKLN
GDTTENKTAYYKADTTRTLYWDGFMPKIVVQGYQADGAGNGKATHEAANRPLLQQWFE
TPANWEKLSEQLTKKLGSDKFKNVVLTAPTVSYEDGGSNTRIPKVTFTLQGKDGYILQ
TDNGATSSLTLSIRVLYTSASSTQNALRYQGASSSAAPSGANTADHAKTIKDVNVYLN
YTGPAIVLDATLPVVGKVDNTTTNGTSNVTDDLNTKFKDLVTSDQRTASLFFQAIQKY
VNTFDPKYPANSISDQKNGVTWARVENKALKIANLNNPNAVFVQQMKDDSEAVYLPVI
GVANNRWLNTFLIRIPLTKFVKPLTVFTATPASTPTQDSSASNTSPQTTT

>KXD88_RS01070-334
MPKIVVQGYQADGYVADGQGNNRAAHEDANKTKLQNWFNAPANW
EKLSEQLTKKLGADKFKNVTLSSPTVTYETISTGRDVRIPKVTFTVTVKNGYNLIAPE
GDAKQISLSIRVLYTSASSTENALRYQGVSFSATPKDASVENPATAIKNVNVYLNYTG
PSIVLDQAVPAVGTANNTSINGTSNVEGTFNTKFKKLLVNVVRGGHAESSLFQAVINY
VNKFDPKFRAQFVTNSINGVTITKVESGTQLRPGTLDDLVKNDRNNVFLQQMKNDTEA
VYLPVTALTNDKWLNTVLVRIPLTKFVKTLSVFQATTAAPQDAAQTSEAQGSQPAGQS

>KXD88_RS01075-328
MPKIVVEGYQPAGHGSNKEAHETENTRLLQQWFATESNWEKLSE
QLTKKLGSDKFKNVTLTNPQVSYEEVTVNSNTWKNPKVTFTVEAKPGYELTQPTTDPK
QISLTIRVLYSSQESNQNLLTIQGASSSAAPRNATVNDANVKANVNVYLNYTGPSIVL
DADLPTVGEQENTSLNGTSNVDGDFNTAFRGNPSSGLLFTNRYANPLLKSVINYVNKF
DPKYRAEFVTNSKNGVTITKVESDTQLRPGTLDDLNKNNVFLQQIQGDTEAVYFAVTA
IASNNWLNTFLIRIPLTKFVKPLTEFRPTTPTSTSSDTQQQGTAQTQSNQGS

>KXD88_RS01080-325
MPTYEPDTTDDNKAAHEEANKTKLEAWFTNSANWAGLEDQLTKR
LGSDKFKNVKLTYASLTLTDFTASRGVTRWNPTVKFSIKANEGYTLDTNSTKEIDLVV
RVLYKSAEANAANVFTHEGASSSAAPKNTMTPNNAEVIKNVNVYLNYTGPSIVLDEAL
PTVGGKENTSINGTSNVDGAFNNAFRGNPSKGLLFTDRYPNPLLKSVINYVNKFDPKY
RATFVTDSRDGVTISNVTNNVQLRPGTLDDLLSNKNKVFLQQIKGDTEAVYFAVTAIA
SNNWLNTFLIRIPLTKFVRPLTEFQAQSEAAPTEETSGEQDSAPTGTGL

>KXD88_RS01085-334

MTKIVLEGFDSRWTNVNDNKSKLQTWFNTKANWSNLEDQLVKKL
GSEKFKNVVLSQPNVTLETFTWNRQTWGVPTVTFNLAAKEGYEKAKNSTNTIALKIRV
VYNSNNTNEILFPIQGASSSAAPSGANTADHAKTIKDVNVYLNYTGPAIVLDATLPKV
GKVDNTTTNGTSNVTDDLNTKFKDLVTSDQRTASLFFQAIQKYVNTFDPKYPANSISD
QKNGVTWARVENKALKIANLNNPNAVFVQQMKDDSEAVYLPVIGVANNRWLNTFLIRI
PLTKFVKPLTVFLLLQLGLLLKIHQLQIQAHKQQLKVHNNKTKNMPQNVFLILRRVYT

>KXD88_RS01090-361
MPKIVIDDYVADGYDVANNAENNRAQHEAANRPLLEQWFKVNQD
KLSLVAEQLTKKLGEEKFKNMTLSNPEISWDEVKFSNKVSNKGYLTPKVTFNLTPKED
YKKASDSSEKVTLTIRNLYKEANSDTNVFATQGAYYTATPSDNATTIKKVNVYLNYTG
PNIELDADLPRVGEQENTSLNGTSNVTDDFNNKFNKLLIRYKAETSLFQTIINYVNKF
DPKFPAKFVTNSINGVTITKVQSDTQLRPGTLDDLLKNRNNVFLQQMKNDTKAVYFAV
NGVTSQGWLNTLLIRIPLTKFVRPISVFTAPVVTPPTQDDSQEDSSNAGNTNENTETP
VEDQTGQESSESRSTQTDQAQQESPQT

>KXD88_RS01095-347
MPKIVVEDYVSDGDGNNRAQHEAANQQKLQEWFNTSSNWEKLSE
QLTKKLGADKFKNVTLTNPQVSYEEVTVNSNAWKNPKVTFNIQAKPGYQLTEPTTEES
QISLSIRVLYSSQESNQNLLTIQGASPTAAPNNTTMVSDPNVLRTVNVYLNYTGPNIE
LDAELPQVGNQNNTSINGTSNVEGTFNDKFKKLLVNVVGESITQTSLLQAIINYVNKF
DPKFPASFVLKTNGVAIASVQNNTQLRIGSLNDFLYNNKGFLQQVNTDTKAVYFAVMG
VTSNGWVNTFLIRIPLTKFVRPISVLEEQTTEEETQESQEGQENQEQGSTTQTNTESS
GTGQSSDLTEVSA

>KXD88_RS01100-317
MPKIVLEGFDSTWGRNSDNQDKIRAWFGDAANWAGLSEQLTKKL
GAERFKNVVLSNPQIIFEDVMLSSGSVKFPKVTFTVAAKDGYQLASGEGTSTTLELKI
RVLYSSSDPNALLLPYQGASSSAAPRGATVNDPNVKSKVNVYLNYTGPNIVLDADLPT
VGGKENTSINGTSNVEGTFNDKFKELLVDNNRVPREFLSTVINYVNKFDPKFRAQLVT
DTNGVALTSVQNGIQLRIGNLNDLLYNKKVFLQQVHGDSSAVYFAVTAIASNNWLNTF
LIRIPLTKFVRPLTAFTATPASAPTQTDSGTGAQTQPEAQS

>KXD88_RS01115-308
MPKIVVSLLTTKAANQEYDASVRQGGVVFWKTRNGEILDVWFAA
NQDKFTLVADQLTKKLGEEKFKNMTLSNPTITWDELTIPEGTILTPKVTFTLTPKDGY
TLADNSENTVTLTIRNLYKAADPNTNLFATQGASPSAAPGNTTEPNNATVIKNVNVYL
NYTGPSIVLDEALPTVGGQENTSINGTSNVDGDFNTAFRGNPSSGLLFTNRYPNPLLK
SVINYVNKFDPKYRAQFVTDSKNGVTITKVESGTQLRPGTLDDLDKDNVFLQQIQGDT
EAVYFAVTAVASNRWLNTFLIRIPLTKYLLNL

>KXD88_RS01130-357
MPKIVVEDYQPAGHGNNKDTNETTNQQKVQEWFNTSSNWEKLAE
QLTKKLGANKFKNVVLTAPTVSYEDGGSNTRIPKVTFTLHGKDGYTLQTDNGATSSLT

LSIRVLYTSASSTQNALRYQGASSSAAPSGSTPSNNAAVIRNVNVYMNYTGPNIELDT
ELPQVGNQNNTSINGTSNVDGDFNTKFKNLLVNVVGGSFIQTSLLQAIINYVNKFDPK
FRAAFVTSINGVALTSVQNDTQLRIGSLNDFLYNNKGFLQQVNGDSSAVYFAVNGVTS
EGWLNTFLIRIPLTKFVRPITPFTASAVMIPTTEDYQEDSSNAGNTNENTETPVEDQT
GQESSESGSTQTDQAQQESPQTQ

>KXD88_RS01135-394
MSEEAAAEAETQTPAPTPAADLASTVSYLKSLNDTLRAATDALN
GDNPTTKTAYYKADTGRTLYWDGFMPKIVVQGYQADGAGNGKATHEAANKTKLQQWF
ATESNWEKLSEQLTKKLGSDKFKNVVLTAPTVSYEEVTVNSNTWKNPKVTFNIQAKPGY
QLTQPTTDPKQISLTIRVLYSSQESNQNLLTIQGASPTAAPSNSSVDDANVKAKVNVY
LNYTGPNIVLDADLPTVGGKENTSINGTSNVEGTFNTKFKNLLVNVVGGSFTQTSLFQ
AIINYVNKFDPKFRAAFVTSINGVALTSVQSGTQLRIGNLNDFLYNKKVFLQQVHGDS
SAVYFAVTAIASNNWLNTFLIRIPLTKFVRPLTAFTATPASAPTQTDSGTGAQTQPEA
QS

>KXD88_RS01160-374
MPKIVVSLLTTKAANQEYDASVRQGGVVFWKKRNGEILDVWFAA
NQDKFTLVADQLTKKLGEEKFKNMTLSNPTITWDELTIPEGTILTPKVTFTLTPKDGY
TLADNSENTVTLTIRNLYKAADPNTNLFATQGASPSAAPKNTKTPNDAEVIKNVNVYL
NYTGPSIVLDQDLPTVGGQENTSINGTSNVTGDFNNAFRGNPSSGLLFTNRYPNPLLK
SVINYVNKFDPKFRAEFVTNATNGVTITKVQSGTQLRPGTLDDLNKNNVFLQQIKGDT
EAVYFAVSAIASNGWLNTFLIRIPLTKFVRPISVLQTPDQQQTQEGGEEATQPQNAGS
GTASTSEVQSGQSNGPVGSGTGSTQQAQSSSTTGTSSSSN

>KXD88_RS01175-359
MPKIVVDDGYVEDTEENNKENNKNTNQQKLEAWFAANQDKFTLV
ADQLTRKLGEEKFKNMTLSNPTISWDESVWKTKTILTPKVTFNLTPKEGYEKATGSLE
TVTLTIRNLYKEANPDTNVFATQGASSSATPNNASQANDATTIEKVNVYLNYTGPSIV
LDEALPTVGATPNTSINGTSNVTGDFNTKFKKLLVSGTTQTPLLQTIINYVNKFDPKF
PASFVLKTNGVAIASVQNNTQLKIGSLNDFLWNNKGFLQQVNTDTKAVYFAVIGVASN
GWLNTFLIRIPLTKFVRSITPFTAPAVMTPTTEDSQEESRENSSQADNTEQNAENQAD
GQSGGEATETQEAQGDSASQSSPQT

>KXD88_RS01180-300
MPRIVVEGYQPAGHGSNKEAHETTNQNQEKLQNWFNAPANWEKL
AEQLTKKLGADKFKNVTLTNPIVTYEEITVDKYTWKNPKVTFAVEAKPGYQLASGAGT
STTLELKIRVLYTSESSTQNLLTIQEASSSAAPSGANTADHAKTIKDVNVYLNYTGPA
IVLDTTLPEVGNANNTIINGTSPITDETLAAKVKTLFNKTTNTNDPTELMLAITKYVQ
TFDPKYLSTRTDNGYRAWVSWMWTNRSALSQRELKQPADIFLQQMKDDTEAVYLPIGG
TTDDKWLNTFLIRIPLNKFVKAIL

>KXD88_RS01195-342
MPKIVLTDFDKTWGQNTANQTKIRDWFREASNWEGLSDQLTKKL

GVERFKNVVLSQPNVTFEIINWNGQTWRVPTVTFNLAAKEGYELIGSTNTIALKIRVV
YDSNDANAILFPIQGASSSAVPNRASNPNDATTIKNVNVYLNYTGPAIVLNADLPTVG
GQENTSINGTSNVDGTFNTAFRGNPSSGLLFTHRYANPLLKSVINYVNKFDPKFRAEF
VTDSKNGVTISNVTNNVQLRPGTLDDLLSNKNKVFLQQIKGDTEAVYFAVNGVTSEGW
LNTFLIRIPLTKFVRPLTEFQAQSATVPTQEGQTTQGQENSAGSGALGSTTGTQTTSG
GSSQASGS

>KXD88_RS01210-340
MPKIVVDDGYVEDTEENNKENNKNTNQKKLEAWFKANQDKFTLV
ADQLTRKLGEEKFKNMTLSNPTISWDEVKFSKGNVTKLYLTPKVTFNLAAKEEYEKSS
DSLETVTLTIRNLYKETNPDTNVFATQGASSSATPNNASQANDATAIEKVNVYLNYTG
PSIVLDEALPTVGATPNTSLNGTSNVTGEFNTKFRKLLVSVFRQGHAESSLFQAVINY
VNKFDPKYRATFVTNSINGVTITKVQNTKELRPGTLDDLVKNDRNNVFLQQIQRDTEA
VYLPVTAVTIDKWLNTVLVRIPLTKFVRSITPFTAPVVTPPAEEATEGSQTQASTPQT
MPNSSQ

>KXD88_RS01265-338
MPKIVVQGYQADGYVATGAGDNKTANEAANLEKLKTWFDIPANW
EKLSEQLTKKLGSDKFKNVVLTAPTVSYEEVTVNSNTWKNPKVTFNIQAKPGYQLTQP
TTDPKQISLTIRVLYSSQESNQNLLTIQGASPTAAPSNSSVDDANVKAKVNVYLNYTG
PNIVLDADLPTVGGKENTSINGTSNVEGTFNTKFKNLLVNVVGGSFTQTSLFQAIINY
VNKFDPKFPASFVLKTNGVAIASVQNNTQLKIGSLNDFLWNNKGFLQQVNTDTKAVYF
AVIGVASNRWLNTFLIRIPLTKFVKPLTEFQAQSPTAPTQEASGDQVQADPQATQMGT
QAQS

>KXD88_RS01270-330
MPKIVVEGYEADGEGNGKATHEDANKALLQQWFDTPANWEKLSE
QLTKKLGSDKFKNVTLANPVISYDDVSTGRDVRIPKVTFTVAAKDGYNLTEPTGNTKQ
ISLSIRVLYTSATSTQNALRYQGVAFSGAPSGSLVDDANVKAKVNVYMNYTGPAIVLD
QAVPAVGTATNTSLNGTSNVTGDFNNKFNKLLIRYKAETSLFQTIINYVNKFDPKFRA
QFVTNSINGVTITKVQNTKELRPGTLDDLVKNRNNVFLQQVHGDSSAVYFAVTALTND
KWLNTVLVRIPLTKFVRPISVLEEQSAQDQTPEASQQADGDQSTSSSPQGTAQT

>KXD88_RS01280-271
MPKIVLTDFDKTWAQNSENEGKIREWFATVSNWATLSDQLTKKL
GAERFKNVKLTFKEASFSSDNIKTPTVTFTVAAQDGYTLDNSTNEISLVVRVLYNRDN
ESTIQLPTQGDSSSAAPAGSTPSNNAAVIRDVNVYMNYTGPALILNAALPTVGNAANT
SLNGTSNVTDEDFNAAFRGLLFTNRYVNPLMQSVINYVNKFDPKYRAMFVTNAIDGAA
LTKVQNKKELRPGNLEDLLYNNNVFLQQMKNDTEAVYFAVTALTIDKWLNTVL

6443
19,271-394

>KXD88_RS01065-160

MPKIVVDDGYVENTEENNKENNKNTNQQKLEAWFKANQDKFTLV
ADQLTRKLGEEKFKNMTLSNPTISWDEVKFSKGNVTKLYLTPKVTFNLAAKEGYEKAS
DSSETVTLTIRNLYKEANPDTNVFATQGASSSAAPRGATVNDTNVKAKVNVYLNYTDI

>KXD88_RS01125-148
MPKIVLEGFDSTWGRNSENQTKIREWFGNAANWEGLSDQLTKKL
GAERFKNVKLTYKEVTFSTNAVKTPTVTFTVAAKDGYTLVDSGKNASSTEISLVVRVL
YNRDNESIIQLPTQGASSSAAPNNAKNANDKTTKRTVNVYLNYTDI

>KXD88_RS01145-148
MPKIVLTDFDKTWEQNSEENERKIRKWFDNAANWVGLSDQLTKK
LGAERFKNVKLTYKEVTFSTNAIKTPTVKFTVAAKEGYTLTTGDNASGTEISLVVRVL
YNRGSENVIQLPTQGASSSAAPNNAKNANDKTTKRTVNVYLNYTDI

>KXD88_RS01155-148
MPKIVLTDFDKTWEQNSENERKIREWFREAANWATLSDQLTKKL
GAERFKNVKLTFKEANFSISSDNIKTPTVKFTVKAKDGYTLTTGDNASATEISLVVRV
LYNHDDESTIQLPTQGASPSAAPSGSSVNDAKVKAKVNVYLNYTDI

>KXD88_RS01190-147
MPKIVLTDFDKTWGQNSEENERKIREWFREASNWAGLSDQLTKK
LGAERFKNVKLTYKEVTFSTNAVKTPTVTFTVAAKDGYTLTTGDNASATEISLVVRVL
YNHDDESTIQLPTQGASPSAAPSGSSVNDAKVKAKVNVYLNYTDI

>KXD88_RS01205-153
MPKIVLEGFDSTWRENSEENERKIREWFREASNWAGLSDQLTKK
LGAERFKNVKLTFKEASFSSDNIKTPTVTFTVAAKDGYTLTTGDNASATEISLVVRVL
YNHDDESTIQLPTQGASSSAAPNTASANNPALAIKNVNVYLNYINQFKLTN

>KXD88_RS01220-152
MPKIVLEGFDSTWRENSANQDKIRAWFGDAANWAGLSDQLTKKL
GAERFKNVKLTFKEASFSSDNFKTPTVTFTVAAKDGYTLTTGDNASATEISLVVRVLY
NHDDESTIQLPTQGASSSAAPNTASANNPALAIKNVNVYLNYINQFKLTN

>KXD88_RS01240-157
MPKIVVDDGYVEDTEENNKENNKNTNQKKLEAWFGDAANWAGLE
DQLTKRLGSDKFKNVKLTYTSVTLTDFTAGRGVTRWNPTVKFSVKANEGYTLDTNSTK
EIDLVVRVLYKSAEANAANVFTHEGASSSATSRGATFNDPNVKSKVNVYLNYTDI

>KXD88_RS01245-149
MPRIVLSPLPEEVARANKEYDASIRQGGVVFWKTRNGEILDVWF
AANQDKFTLVADQLTRKLGEEKFKNMTLSNPTISWDEVKFSKGNVTKLYLTPKVTFNL
AAKEGYEKATDSLEKVTLTIRNLYKEANPDTNVFATQGTASSSVSEV

>KXD88_RS01260-156
MPKIVVSGYEADGAGVNKAQHEEANKTKLQQWFATESNWEKLSE
QLTKKLGADKFKNVKLTYKSITLTDFTVDRATRWYPTVKFSVEANEGYTLNTNSTKEI
DLVVRVLYKSAEANAANVFTHEGASSSATPNNAKNANDKTTKRTVNVYLNYTDI

>KXD88_RS01290-166
MPKIVTTALPTTVAPMNREYNESPTGGHEFWKGRNKQVLDAWFA
ANQDKLSLVAEQLTKKLGKDKFKNMVLSTPTITWDEVEFSGRRYLTSKVTFTLTPKDG
YEKVSDSSETVTLTIRNLYKEANPDTNIFATQGTSSSAAPDNTTEPNNATVIKNVNVY
LNYTDI

1684
11,147-166

Mycoplasmopsis synoviae strain G3

>K6988_RS00080-334
MPKIVVEGYVADTTGSSKDTNETANKTKLQTWFDTKANWAGLED
QLTKKLGADKFKNVVLTSPMVSYEEITVNSNTWKNPKVTFTVEAKPGYELTQPTTDPK
QISLTIRVLYENQTSTQNLLTIQGASPTAAPSNSSVDDANVKAKVNVYLNYTGPAIVL
DQDLPTVGTANNTSLNGTSNVEGTFNTKFKNLLVNVVGGSFTQTSLLQAIINYVNKFD
PKFRAAFVTSINGVALTSVQSGTQLKIGSLNDFLYNNKGFLQQVNGDSSAVYFAVNGV
TNKGWLNTFLIRIPLAKFVKPLTEFRPTTPTSPSSDTQQQGTEQTGETQGSQPAGQSQ

>K6988_RS00085-343
MPKIVVDGYVAAGYEADSAGTNKAQHETANQEKLQQWFEANQDK
LSLVAEQLTKKLGEEKFKNMTLSNPTISWDEVKFSNKGSNKGYLTPKVTFNLAAKDGY
TLAKESANTVTLTIRVLYKDSNLTRNVLSTQGASSSAAPAGSTPSNNAAVIRDVNVYM
NYTGPAIVLDQAVPAVVTAANTLINGTSNVDGAFNTAFRGLLFTTRYANPLLKSVINY
VNRFDPKYRAEFVTNATNGVALTKVQKDRQKKDRELRPGNLEDLLYNDNVFLQQMKND
TEAVYFAVTALTNDKWLNTVLVRIPLTKFVKPLSVFTAPVVTPPAEEDTEGSQTQTST
PQTMPNSSQ

>K6988_RS00100-336
MPKIVNPALPEEVAPRNREYNESPNGGYPFWKERNALILTTWFN
QNQDKLSLVADQLTKKLGEEKFKNMTLSNPRITWDELVRTEGTILTPKVTFNLAAKDG
YTLAEGSATTVTLTIRVLYKDSNPTRNVLSTQGASYSSAPRGATVNDANVKAKVNVYM
NYTGPSIVLDEAHPTVGATPNTSINGTSNVDGDFNTKFKNLLVNVVGGSFTQTSLLQA
IINYVNKFDPKFRAAFVLNTNGVAITKVENTKELRPGTLDDLLSNKDIVFLQQIKGDT
KAVYFAVTAIASNNWLNTFLIRIPLTKFVRPLTAFTATPASAPTQTDSGTGAQTQPEA
QS

>K6988_RS00120-468
MQFTSGSVTAAKAVKEDAEYSKVTDTTHKTTLEEKYTAAADFLT

EDSKLANLDASSNLDTTKATLESAKTALDAAVAAVKPDLDFQKTKTSAAAKVTELESL
VNTALKAELQRQVNELTKEQAAQATTMLENLTSLKDSLTSLQDLVSKGLEMQVDYPRN
YYDADNKTAFDDALLKASSVFPAFKWTAQSIMVPAPEEGALPNPRAWTKAREKSEFVL
QNFVMAPAPAPAPAQAAPATQSDSTAAAAASATVRLATGTEEAVEDSESSETQTPTPA
ADLASTVSYLKSLSDSLKAETDKLNGDTTENKTAYYKADTGRTLYWDGFMPKIVLTDF
DKTWEQNSEENERKIRKWFDNAANWVGLSDQLTKKLGAERFKNVKLTFKEASFSSDNI
KTPTVTFTVEAQDGYTLDNSANEISLVVRVLYNRGSESTIQLPTQGASSSAAPRGATV
NDANVKAKVNVYLNYTDI

>K6988_RS00180-327
MPKIVVDDGYVENTEENNKENNKNTNQQKLEAWFAANQDKFTLV
ADQLTRKLGEEKFKNMTLSNPRITWDEVEFSNKGSNKLYLTPKVTFNLAAKGGYGKAT
DSLEKVTLTIRNLYKEANPDTNLFATQGASSSAAPSGSSVDDAKVKANVNVYLNYTGP
SIVLDKAIPAVGTANNTSINGTSNVEGTFNDKFKELLVDNNLVPREFLSTVINYVNKF
DPKFRAQLVTDINGVAITRVQSKKELRIGNLNDLLYNKNVFLQQVHGDSSAVYFAVNG
VTSEGWLNTFLIRIPLTKFVRPLTAFTATPASAPTQTDSGTGAQTQPEAQS

>K6988_RS00185-328
MPKIVVEDYVSDGDGNNRAQHEAANQQKLQEWFNTSSNWEKLSE
QLTKKLGADKFKNVTLTNPQVSYEEVTVNSNAWKNPKVTFNIQAKPGYQLTEPTTEES
QISLSIRVLYSSQESNQNLLTIQGASPTAAPNNTTMVSDPNVLRTVNVYLNYTGPNIE
LDAELPQVGNQNNTSINGTSNVDGAFNTAFRGNPSRGLLFTHRYANPLLKSVINYVNK
FDPKFRAEFVTDSKNGVTISNVTNNVQLRPGTLDDLNGNNVFLQQIQGDTKAVYFAVT
AIASNGWLNTFLIRIPLTKFVRPLTEFQAQSEAAPTEETSGEQDSAPTGTGS

>K6988_RS03650-325
MPKIVLNNFDKTWGQNTTNQDKIKDWFREASNWAGLSEQLTKKL
GVDKFKNVVLSQPNVTFEIINWNGQTWRVPTVTFNLAAKEGYELTGSTNTIALKIRVV
YDSNDANAILFPIQGASSSAVPNRASSPNDEKTIKTVNVYLNYTGPNIELDAELPKVG
SQENTSLNGTSNVTDDFNNKFNKLLIRYKTETSLFQTIINYVNKFDPKFPAKFVTNSI
NGVTITKVEKIKELRPGTLDDLLKNRNNVFLQQIKGDKEAVYFAVTAVTIDKWLNTVL
VRIPLTKFVRPISVLEEQSAQDQTPEASQQAPEGGSLASGTAPTEGAQS

>K6988_RS00195-339
MPKIVVPTYEPDTTADNKAAHEETNKTKLEAWFGDAANWVGLED
QLTKRLGSDKFKNVKLTYASVTLTDFTAGRGVTRWNPTVKFRVEANEGYTLDTNSTRE
IDLVVRVLYKSAKSNAANVFTHEGASSSAAPGNTKTPNDPKVIKNVNVYLNYTGPSIV
LDEALPTVGATPNTSINGTSNVTGAFNTAFRGNPSKGLLFTSRYPNPLLQSVINYVNK
FDPKFRAEFVTDSKNGVTITKVEKIKELRPGTLDDLNNNNVFLQQIQGDTKAIYFAVT
AIASNNWLNTFLIRIPLTKFVKPVTAFMPAAPTASVPTQDSSASNTIHKQQLKVHNNK
TKNMP

>K6988_RS00200-331
MPKIVVRLLTTKPANQEYDASVRQGGVVFRKTRNGEILDVWFAA

NQDKFTLVADQLTRKLGKDKFKNMTLSNPRITWDELTIPEGTILTPKVTFTLTPKEGY
EKASDSSETVTLTIRNLYKDSNPEVNVFQTQGSLPSAAPRGATVNDPNVKSKVNVYLN
YTGPSIVLDEALPTVGATPNTSINGTSNVEGTFNNAFRGNPSRGLLFTSRYPNPLLQS
VINYVNKFDPKYRAEFVTNSIDGVTITKVLKDKKQKDREELRPGTLDDLLSNKNKVFL
QQIKGDTEAVYFEVTAIASNNWLNTFLIRIPLTKFVRPISVFQATTEAPQDAAQN

>K6988_RS00205-311
MPKIVLEGFDSRWTNVNDNKSKLQTWFNTKANWSNLEDQLVKKL
GSEKFKNVVLSQPNVTLETFTWNRQTWGVPTVTFNLAAKEGYEKAKNSTNTIALKIRV
VYNSNNTNEILFPIQGASSSAAPSGANTADHTKTIKDVNVYLNYTGPAIILDATLPEV
GKVDNTTTNGTSNVTDDLNTKFKDLVTSDQRTASLFFQAIQKYINTFDPKYPANPISD
QKNGVTWARVENKALKIANLNNPNAVFVQQMKDDSEAVYLPVIGIANNRWLNTFLIRI
PLTKFVKPLTVFTATPASTPTQDSSASNTSPQTTT

3442
10,311-468

>K6988_RS00090-131
MPKIVVEGYVADTTGSSKDTNETANKTKLEAWFAANQDKFTLVA
DQLTRKLGSDKFKNVTLTNPIVTYEEITVNSNTWKNPKVTFNIQAKPGYQLTEPTKDP
KQISLTIRVLYKIRLLLKTCLLFKELLLL

>K6988_RS00130-162
MPKIVVEDYVSDGDGNNRAQHEAANQQKLQEWFNTSSNWEKLSE
QLTKKLGADKFKNVTLTNPQVSYEEVTVNTYTWKNPKVTFSIQAKPGYQLTEPTTNPK
QISITIRVLYLSQESNQNLLTIKGAFPSAAPNTASGDNPASVIKNVNVYLNYINQFKL
TN

>K6988_RS00145-187
MPKIVVEDYQPTSHGRNKENDETTNQNQEKLQNWFNAHANWEKL
SEQLTKKLGYDKFKNVTLTNPVISYDDVSTGRDVRIPKVTFTVAAKDGYNLTEPTGEK
KQITLSIRVLYTSQNQTENALRYQGVSFSAVPNRASNPNDANVMRNVNVYLNYTGPAI
VLDATLPEVGALIIQPLMVLLQLQMKL

>K6988_RS00155-135
MPKIVLEGFDKTWEQNSEENERKIREWFREASNWVGLSDQLTKK
LGAERFKNVKLTYKEVTFSTNAVKTPTVTFTVEAQDGYTLDNSANEISLVVRVLYNRG
SENVIQLPTQGASSSAAPGNPTEPNNATVIKKR

>K6988_RS00170-171
MADLASTASYLKSLSDSLKAETDKLNGDTTENKTAYYKADTTRT
LYWDGFMPKIVVEDYQSAGHGNNKDTNETANKTKLQNWFNTSSNWEKLAEQLTKKLGA
DKFKNVTLSSPTVTYETISTGSEVRIPKVTFTLQGKEGYELQTTDGASATLSLSIRVL
YTSENQTEKRT

786
5，131-187

Mycoplasmopsis synoviae strain HN01

>EJ916_RS00980-271
MPKIVLTDFDKTWAQNSENEGKIREWFATVSNWATLSDQLTKKL
GAERFKNVKLTFKEASFSSDNIKTPTVTFTVAAQDGYTLDNSTNEISLVVRVLYNRDN
ESTIQLPTQGDSSSAAPAGSTPSNNAAVIRDVNVYMNYTGPALILNAALPTVGNAANT
SLNGTSNVTDEDFNAAFRGLLFTNRYVNPLMQSVINYVNKFDPKYRAMFVTNAIDGAA
LTKVQNKKELRPGNLEDLLYNNNVFLQQMKNDTEAVYFAVTALTIDKWLNTVL

>EJ916_RS00990-330
MPKIVVEGYEADGEGNGKATHEDANKALLQQWFDTPANWEKLSE
QLTKKLGSDKFKNVTLANPVISYDDVSTGRDVRIPKVTFTVAAKDGYNLTEPTGNTKQ
ISLSIRVLYTSATSTQNALRYQGVAFSGAPSGSLVDDANVKAKVNVYMNYTGPAIVLD
QAVPAVGTATNTSLNGTSNVTGDFNNKFNKLLIRYKAETSLFQTIINYVNKFDPKFRA
QFVTNSINGVTITKVQNTKELRPGTLDDLVKNRNNVFLQQVHGDSSAVYFAVTALTND
KWLNTVLVRIPLTKFVRPISVLEEQSAQDQTPEASQQADGDQSTSSSPQGTAQT

>EJ916_RS00995-329
MPKIVVQGYQADGYVATGAGDNKTANEAANLEKLKTWFDIPANW
EKLSEQLTKKLGADKFKNVVLTAPTVSYEDNGSNTRIPKVTFTLQGKDGYILQTDNGA
TSSLTLSIRVLYTTQSPNANVLQMQGASPTAAPGNTMKPNDDAVIKNVNVYLNYTGPS
IVLDVAHPTVGGQENTSINGTSNVDGAFNTAFRENPSRGLLFTNRYANPFLKSVINYV
NKFDPKYRAAFVTEKNGVAIASVQSGTQLKIGSLNDFLYNNKGFLQQVHGDSTAVYFA
VTAIASNNWLNTFLIRIPLTKFVKPLIAFTATPASAPTQTDSGTASQPQQAAS

>EJ916_RS03710-331
MHKIVVPTYEPDTTSDSKATHETTNQQKLQNWFNAPANWEKLSE
QLTKKLGADKFKNVKLTYKSITLTDFTVDRATRWYPTVKFSVEANEGYTLNTNSTKEI
DLVVRVLYKSAEANAANVFTHEGASSSAAPKNTKTPNNAEVIKNVNVYLNYTGPSIVL
DVAHPTVGGQENTSINGTSNVDGAFNTAFRENPSKGLLFTNRYANPLLKSVINYVNKF
DPKYRAAFVTSINGVTITKVQNTKELRPGTLDDLNKNNVFLQQIKGDKEAVYFAVSTI
ASNNWLNTFLIRIPLTKFIKPLSVFTAPVVTPPAEEATETQEAQGDSASQSSLQT

>EJ916_RS01050-340
MPKIVVDDGYVEDTEENNKENNKNTNQKKLEAWFKANQDKFTLV
ADQLTRKLGEEKFKNMTLSNPTISWDEVKFSKGNVTKLYLTPKVTFNLAAKEEYEKSS
DSLETVTLTIRNLYKETNPDTNVFATQGASSSATPNNASQANDATAIEKVNVYLNYTG
PSIVLDEALPTVGATPNTSLNGTSNVTGEFNTKFRKLLVSVFRQGHAESSLFQAVINY
VNKFDPKYRATFVTNSINGVTITKVQNTKELRPGTLDDLVKNDRNNVFLQQIQRDTEA
VYLPVTAVTIDKWLNTVLVRIPLTKFVRSITPFTAPVVTPPAEEATEGSQTQASTPQT

MPNSSQ

>EJ916_RS01065-342
MPKIVLTDFDKTWGQNTANQTKIRDWFREASNWEGLSDQLTKKL
GVERFKNVVLSQPNVTFEIINWNGQTWRVPTVTFNLAAKEGYELIGSTNTIALKIRVV
YDSNDANAILFPIQGASSSAVPNRASNPNDATTIKNVNVYLNYTGPAIVLNADLPTVG
GQENTSINGTSNVDGTFNTAFRGNPSSGLLFTHRYANPLLKSVINYVNKFDPKFRAEF
VTDSKNGVTISNVTNNVQLRPGTLDDLLSNKNKVFLQQIKGDTEAVYFAVNGVTSEGW
LNTFLIRIPLTKFVRPLTEFQAQSATVPTQEGQTTQGQENSAGSGALGSTTGTQTTSG
GSSQASGS

>EJ916_RS01080-300
MPRIVVEGYQPAGHGSNKEAHETTNQNQEKLQNWFNAPANWEKL
AEQLTKKLGADKFKNVTLTNPIVTYEEITVDKYTWKNPKVTFAVEAKPGYQLASGAGT
STTLELKIRVLYTSESSTQNLLTIQEASSSAAPSGANTADHAKTIKDVNVYLNYTGPA
IVLDTTLPEVGNANNTIINGTSPITDETLAAKVKTLFNKTTNTNDPTELMLAITKYVQ
TFDPKYLSTRTDNGYRAWVSWMWTNRSALSQRELKQPADIFLQQMKDDTEAVYLPIGG
TTDDKWLNTFLIRIPLNKFVKAIL

>EJ916_RS01085-359
MPKIVVDDGYVEDTEENNKENNKNTNQQKLEAWFAANQDKFTLV
ADQLTRKLGEEKFKNMTLSNPTISWDESVWKTKTILTPKVTFNLTPKEGYEKATGSLE
TVTLTIRNLYKEANPDTNVFATQGASSSATPNNASQANDATTIEKVNVYLNYTGPSIV
LDEALPTVGATPNTSINGTSNVTGDFNTKFKKLLVSGTTQTPLLQTIINYVNKFDPKF
PASFVLKTNGVAIASVQNNTQLKIGSLNDFLWNNKGFLQQVNTDTKAVYFAVIGVASN
GWLNTFLIRIPLTKFVRSITPFTAPAVMTPTTEDSQEESRENSSQADNTEQNAENQAD
GQSGGEATETQEAQGDSASQSSPQT

>EJ916_RS01100-374
MPKIVVDGYVADGYEADSAGTNKAQHETANQEKLQQWFEANQDK
LSLVAEQLTKKLGEEKFKNMILSNPTISWDEVKFSNKGSNKGYLTPKVTFKLAAKDGY
TLAKESANTVTLTIRVLYKDSNLTRNVLSTQGASSSAAPAGSTPSNNAAVIRDVNVYM
NYTGPNIELDADLPRVGGQENTSINGTSNVTGDFNNAFRGNPSSGLLFTNRYPNPLLK
SVINYVNKFDPKFRAEFVTNATNGVTITKVQSGTQLRPGTLDDLNKNNVFLQQIKGDT
EAVYFAVSAIASNGWLNTFLIRIPLTKFVRPISVLQTPDQQQTQEGGEEATQPQNAGS
GTASTSEVQSGQSNGTVGSGTGSTQQAQSSSTTGTSSSSN

>EJ916_RS01130-394
MSEEAAAEAETQTPAPTPAADLASTVSYLKSLNDTLRAATDALN
GDNPTTKTAYYKADTGRTLYWDGFMPKIVVQGYQADGAGNGKATHEAANRPLLQQWFE
TPANWEKLSEQLTKKLGSDKFKNVVLTAPTVSYEEVTVNSNTWKNPKVTFNIQAKPGY
QLTQPTTDPKQISLTIRVLYSSQESNQNLLTIQGASPTAAPSNSSVDDANVKAKVNVY
LNYTGPNIVLDADLPTVGGKENTSINGTSNVEGTFNTKFKNLLVNVVGGNFTQTSLFQ
AIINYVNKFDPKFRAAFVTSINGVALTSVQSGTQLRIGNLNDFLYNKKVFLQQVHGDS

SAVYFAVTAIASNNWLNTFLIRIPLTKFVRPLTAFTATPASAPTQTDSGTGAQTQPEA
QS

>EJ916_RS01135-357
MPKIVVEDYQPAGHGNNKDTNETTNQQKVQEWFNTSSNWEKLAE
QLTKKLGANKFKNVVLTAPTVSYEDGGSNTRIPKVTFTLHGKDGYTLQTDNGATSSLT
LSIRVLYTSASSTQNALRYQGASSSAAPSGSTPSNNAAVIRNVNVYMNYTGPNIELDT
ELPQVGNQNNTSINGTSNVDGDFNTKFKNLLVNVVGGSFIQTSLLQAIINYVNKFDPK
FRAAFVTSINGVALTSVQNDTQLRIGSLNDFLYNNKGFLQQVNGDSSAVYFAVNGVTS
EGWLNTFLIRIPLTKFVRPITPFTASAVMIPTTEDYQEDSSNAGNTNENTETPVEDQT
GQESSESGSTQTDQAQQESPQTQ

>EJ916_RS01150-343
MPKIVVSLLTTKAANQEYDASVRQGGVVFWKTRNGEILDVWFAA
NQDKFTLVADQLTKKLGEEKFKNMTLSNPTITWDELTIPEGTILTPKVTFTLTPKDGY
TLADNSENTVTLTIRNLYKAADPNTNLFATQGASPSAAPKNTKTPNDAEVIKNVNVYL
NYTGPSIVLDEALPTVGGQENTSINGTSNVTGDFNNAFRGNPSSGLLFTDRYPNPLLK
SVINYVNKFDPKYRATFVTNSRDGVTITKVESDTQLRPGTLDDLNGNNIFLQQIKGDT
EAVYFAVSAIASNRWLNTFLIRIPLTKFVKPLTEFQAQSPTAPTQEASGDQVQADPQA
TQMGTQAQS

>EJ916_RS01160-246
MPKIVLTDFDKTWGQNTANQDKIRAWFGDAANWAGLSDQLTKKL
GAERFKNVVLSNPQIIFEDVRLLTRGSVKFPKVTFTVAAKDGYQLASGEGTSTTLELK
IRVLYSSSDPNALLLPYQGASSSAAPRGATVNDPNVKSKVNVYLNYTGPSIVLDQVVP
AVGTANNTSLNGTSNVTGDFNTKFKKLLVNVVRQGHAESSLFQAVINYVNKFDPKFRA
QFVTNSINGVTITKVQKDKQKRDRELRL

>EJ916_RS03720-317
MPKIVLEGFDSTWGRNSDNQDKIRAWFGDAANWAGLSEQLTKKL
GAERFKNVVLSNPQIIFEDVMLSSGSVKFPKVTFTVAAKDGYQLASGEGTSTTLELKI
RVLYSSSDPNALLLPYQGASSSAAPRGATVNDPNVKSKVNVYLNYTGPNIVLDADLPT
VGGKENTSINGTSNVEGTFNDKFKELLVDNNRVPREFLSTVINYVNKFDPKFRAQLVT
DTNGVALTSVQNGIQLRIGNLNDLLYNKKVFLQQVHGDSSAVYFAVTAIASNNWLNTF
LIRIPLTKFVRPLTAFTATPASAPTQTDSGTGAQTQPEAQS

>EJ916_RS01190-347
MPKIVVEDYVSDGDGNNRAQHEAANQQKLQEWFNTSSNWEKLSE
QLTKKLGADKFKNVTLTNPQVSYEEVTVNSNAWKNPKVTFNIQAKPGYQLTEPTTEES
QISLSIRVLYSSQESNQNLLTIQGASPTAAPNNTTMVSDPNVLRTVNVYLNYTGPNIE
LDAELPQVGNQNNTSINGTSNVEGTFNDKFKKLLVNVVGESITQTSLLQAIINYVNKF
DPKFPASFVLKTNGVAIASVQNNTQLRIGSLNDFLYNNKGFLQQVNTDTKAVYFAVMG
VTSNGWVNTFLIRIPLTKFVRPISVLEEQTTEEETQESQEGQENQEQGSTTQTNTESS
GTGQSSDLTEVSA

>EJ916_RS01195-361
MPKIVIDDYVADGYDVANNAENNRAQHEAANRPLLEQWFKVNQD
KLSLVAEQLTKKLGEEKFKNMTLSNPEISWDEVKFSNKVSNKGYLTPKVTFNLTPKED
YKKASDSSEKVTLTIRNLYKEANSDTNVFATQGAYYTATPSDNATTIKKVNVYLNYTG
PNIELDADLPRVGEQENTSLNGTSNVTDDFNNKFNKLLIRYKAETSLFQTIINYVNKF
DPKFPAKFVTNSINGVTITKVQSDTQLRPGTLDDLLKNRNNVFLQQMKNDTKAVYFAV
NGVTSQGWLNTLLIRIPLTKFVRPISVFTAPVVTPPTQDDSQEDSSNAGNTNENTETP
VEDQTGQESSESRSTQTDQAQQESPQT

>EJ916_RS01200-334
MTKIVLEGFDSRWTNVNDNKSKLQTWFNTKANWSNLEDQLVKKL
GSEKFKNVVLSQPNVTLETFTWNRQTWGVPTVTFNLAAKEGYEKAKNSTNTIALKIRV
VYNSNNTNEILFPIQGASSSAAPSGANTADHAKTIKDVNVYLNYTGPAIVLDATLPKV
GKVDNTTTNGTSNVTDDLNTKFKDLVTSDQRTASLFFQAIQKYVNTFDPKYPANSISD
QKNGVTWARVENKALKIANLNNPNAVFVQQMKDDSEAVYLPVIGVANNRWLNTFLIRI
PLTKFVKPLTVFLLLQLGLLLKIHQLQIQAHKQQLKVHNNKTKNMPQNVFLILRRVYT

>EJ916_RS01205-325
MPTYEPDTTDDNKAAHEEANKTKLEAWFTNSANWAGLEDQLTKR
LGSDKFKNVKLTYASLTLTDFTASRGVTRWNPTVKFSIKANEGYTLDTNSTKEIDLVV
RVLYKSAEANAANVFTHEGASSSAAPKNTMTPNNAEVIKNVNVYLNYTGPSIVLDEAL
PTVGGKENTSINGTSNVDGAFNNAFRGNPSKGLLFTDRYPNPLLKSVINYVNKFDPKY
RATFVTDSRDGVTISNVTNNVQLRPGTLDDLLSNKNKVFLQQIKGDTEAVYFAVTAIA
SNNWLNTFLIRIPLTKFVRPLTEFQAQSEAAPTEETSGEQDSAPTGTGL

>EJ916_RS01210-328
MPKIVVEGYQPAGHGSNKEAHETENTRLLQQWFATESNWEKLSE
QLTKKLGSDKFKNVTLTNPQVSYEEVTVNSNTWKNPKVTFTVEAKPGYELTQPTTDPK
QISLTIRVLYSSQESNQNLLTIQGASSSAAPRNATVNDANVKANVNVYLNYTGPSIVL
DADLPTVGEQENTSLNGTSNVDGDFNTAFRGNPSSGLLFTNRYANPLLKSVINYVNKF
DPKYRAEFVTNSKNGVTITKVESDTQLRPGTLDDLNKNNVFLQQIQGDTEAVYFAVTA
IASNNWLNTFLIRIPLTKFVKPLTEFRPTTPTSTSSDTQQQGTAQTQSNQGS

>EJ916_RS01215-334
MPKIVVQGYQADGYVADGQGNNRAAHEDANKTKLQNWFNAPANW
EKLSEQLTKKLGADKFKNVTLSSPTVTYETISTGRDVRIPKVTFTVTVKNGYNLIAPE
GDAKQISLSIRVLYTSASSTENALRYQGVSFSATPKDASVENPATAIKNVNVYLNYTG
PSIVLDQAVPAVGTANNTSINGTSNVEGTFNTKFKKLLVNVVRGGHAESSLFQAVINY
VNKFDPKFRAQFVTNSINGVTITKVESGTQLRPGTLDDLVKNDRNNVFLQQMKNDTEA
VYLPVTALTNDKWLNTVLVRIPLTKFVKTLSVFQATTAAPQDAAQTSEAQGSQPAGQS

6662
20, 246-394

>EJ916_RS00965-166
MPKIVTTALPTTVAPMNREYNESPTGGHEFWKGRNKQVLDAWFA
ANQDKLSLVAEQLTKKLGKDKFKNMVLSTPTITWDEVEFSGRRYLTSKVTFTLTPKDG
YEKVSDSSETVTLTIRNLYKEANPDTNIFATQGTSSSAAPDNTTEPNNATVIKNVNVY
LNYTDI

>EJ916_RS01000-156
MPKIVVSGYEADGAGVNKAQHEEANKTKLQQWFATESNWEKLSE
QLTKKLGADKFKNVKLTYKSITLTDFTVDRATRWYPTVKFSVEANEGYTLNTNSTKEI
DLVVRVLYKSAEANAANVFTHEGASSSATPNNAKNANDKTTKRTVNVYLNYTDI

>EJ916_RS01015-149
MPRIVLSPLPEEVARANKEYDASIRQGGVVFWKTRNGEILDVWF
AANQDKFTLVADQLTRKLGEEKFKNMTLSNPTISWDEVKFSKGNVTKLYLTPKVTFNL
AAKEGYEKATDSLEKVTLTIRNLYKEANPDTNVFATQGTASSSVSEV

>EJ916_RS01020-157
MPKIVVDDGYVEDTEENNKENNKNTNQKKLEAWFGDAANWAGLE
DQLTKRLGSDKFKNVKLTYTSVTLTDFTAGRGVTRWNPTVKFSVKANEGYTLDTNSTK
EIDLVVRVLYKSAEANAANVFTHEGASSSATSRGATFNDPNVKSKVNVYLNYTDI

>EJ916_RS01055-153
MPKIVLEGFDSTWRENSEENERKIREWFREASNWAGLSDQLTKK
LGAERFKNVKLTFKEASFSSDNIKTPTVTFTVAAKDGYTLTTGDNASATEISLVVRVL
YNHDDESTIQLPTQGASSSAAPNTASANNPALAIKNVNVYLNYINQFKLTN

>EJ916_RS01070-147
MPKIVLTDFDKTWGQNSEENERKIREWFREASNWAGLSDQLTKK
LGAERFKNVKLTYKEVTFSTNAVKTPTVTFTVAAKDGYTLTTGDNASATEISLVVRVL
YNHDDESTIQLPTQGASPSAAPSGSSVNDAKVKAKVNVYLNYTDI

>EJ916_RS01105-148
MPKIVLTDFDKTWEQNSENERKIREWFREAANWATLSDQLTKKL
GAERFKNVKLTFKEANFSISSDNIKTPTVKFTVKAKDGYTLTTGDNASATEISLVVRV
LYNHDDESTIQLPTQGASPSAAPSGSSVNDAKVKAKVNVYLNYTDI

>EJ916_RS01115-148
MPKIVLTDFDKTWEQNSEENERKIRKWFDNAANWVGLSDQLTKK
LGAERFKNVKLTYKEVTFSTNAIKTPTVKFTVAAKEGYTLTTGDNASGTEISLVVRVL
YNRGSENVIQLPTQGASSSAAPNNAKNANDKTTKRTVNVYLNYTDI

>EJ916_RS01140-148
MPKIVLEGFDSTWGRNSENQTKIREWFGNAANWEGLSDQLTKKL

GAERFKNVKLTYKEVTFSTNAVKTPTVTFTVAAKDGYTLVDSGKNASSTEISLVVRVL
YNRDNESIIQLPTQGASSSAAPNNAKNANDKTTKRTVNVYLNYTDI

>EJ916_RS01220-160
MPKIVVDDGYVENTEENNKENNKNTNQQKLEAWFKANQDKFTLV
ADQLTRKLGEEKFKNMTLSNPTISWDEVKFSKGNVTKLYLTPKVTFNLAAKEGYEKAS
DSSETVTLTIRNLYKEANPDTNVFATQGASSSAAPRGATVNDTNVKAKVNVYLNYTDI

1532
10, 147-166

Mycoplasmopsis synoviae strain MS-H

>mshv_RS03430-358
MPKIVTTALPTTVAPMNREYNESPTGGHEFWKGRNKQVLDAWFA
ANQDKLSLVAEQLTKKLGEEKFKNMVLSNPTISWDEVKFSNRLYLTPKVTFNLAAKEE
YEKSSDSLETVTLTIRNLYKAAAPNTNLFATQGASSSAAPGNTMKPNDATTIEKVNVY
LNHTGPSIVLDAEFPQVGNQNNTSLNGTSNVTGAFNNAFRGLLFTKRLVNPLMQSVIN
YVNKFDPKFPAAFVTNATNGVTITKVQKDRQKKDRELRPGNLEDLLYNNNVFLQQMKN
DTEAVYFAVTAIASNNWLNTFLIKIPLTKFVRPISVLEEQTTEEETQEGQENQEQEST
AQTNTEFSGTAQSSPSTGTNSGSK

>mshv_RS03445-319
MPKIVVKDYQPAGDGSNKEANKTTNQKKLEAWFGDAANWAGLED
QLTKRLGSDKFKNVKLTYKSITLTDFTVAGATRWNPTVKFSVKAKDDYALDSTSAREI
DIVVRVLYKSAEANAANVFTHEGASSSAAPENTKKPNDPKVIKNVNVYLNYTGPSIVL
DEAHPTVGATPNTSLNGTSNVTGEFNTKFRKLLVSVFRQGHAESSLFQAVINYVNKFD
PKFRAQFVTNSIDGVTITKVQSNTQLRPGTLDDLVKNDRDNVFLQQIQGDTEAVYLPV
TAIASNKWLNTFLIRIPLTKFVRPLTEFQAQSSPTTGTSSSSN

>mshv_RS03470-358
MPKIVTTALPTTVAPMNREYNESPTGGHEFWKGRNKQVLDAWFA
ANQDKLSLVAEQLTKKLGEEKFKNMVLSNPTISWDEVKFSNRLYLTPKVTFNLAAKEE
YEKSSDSLETVTLTIRNLYKAAAPNTNLFATQGASSSAAPGNTMKPNDATTIEKVNVY
LNHTGPSIVLDAEFPQVGNQNNTSLNGTSNVTGAFNNAFRGLLFTKRLVNPLMQSVIN
YVNKFDPKFPAAFVTNATNGVTITKVQKDRQKKDRELRPGNLEDLLYNNNVFLQQMKN
DTEAVYFAVTAIASNNWLNTFLIKIPLTKFVRPISVLEEQTTEEETQEGQENQEQEST
AQTNTEFSGTAQSSPSTGTNSGSK

>mshv_RS03485-306
MPKIVLTDFDKTWEQNSENERKIRKWFDNAVNWAGLSDQLTKKL
GAERFKNVKLTYKEVTFSTNAVKTPTVKFTVAAKEGYTLDNSANEISLVVRVLYNRDN
ESTIQLPTQGASSSAVPNRASSPNDETTIKTVNVYLNYTGPSIVLDEAHPTVGEQENT
SINGTSNVTGEFNTKFRKLLVSVFRQGHAESSLFQAAINYVNKFDPKFRAEFVTDARD

GVTITKVQSNTQLRPGTLDDLVKNDRDNVFLQQIQGDTEAVYLPVTAIASNKWLNTFL
IRIPLTKFVRPLTEFQAQSSPTTGTSSSSN

>mshv_RS03515-331
MPKIVVDDYVADTTENNKEEIKEANKTKLQNWFKAPANWEKLSE
QLTKKLGVDKFKNVKLTYKSITSTDFTAGRGATRWNPTVKFSVKANEGYTLDINSTRE
IDLVVRVLYKSAEANTDNVFTHEGASSSAAPGNTKKPNDPKVIKNVNVYLNYTGPSIV
LDEALPTVGRQENTSINGTSNVTGEFNNAFRGNPSSGLLFTHRYANPLLKSVINYVNK
FDPKYRATFVTNSIDGVTITNVTDKVQLRPGTLDDLKGNNVFLQQIKGDTEAVYFAVT
AIASNKWLNTFLIRIPLTKFVRPISVFQATTAAPQDAAQASEGQGSQPDQTGQTA

>mshv_RS03520-320
MPKIVVNGYVADTTGDNEAAHEEANKTKLQNWFNAPANWEKLSE
QLTKKLGADKFKNVTLSSPTVTYETISTGRDVRIPKVTFTVTAKNGYNLTAPTGNTKQ
ISLLIRVLYTSASSTENALRYQGVSFSATPKDTSVENPATAIKNVNVYLNYTGPSIVL
DQAVPAVGTANNTSLNGTSNVTGAFNNAFRGNPSRGLLFTNRYPNPLLQSVINYVNKF
DPKFPAVFVTNATNGVTITKVESDTQLRIGTLDDLVKNRNNVFLQQIQGDTEAVYLPV
TAIASNNWLNTVLVRIPLTKFVRSISVLEEQSAQDQTPEASQQS

>mshv_RS03525-348
MPKIVVDGYVADGEENNKENNKNTNQQKLEAWFTANQDKFTLVA
DQLTRKLGKEKFKNMTLSNPRITWDEVRFSKGNVTKLYLTPKVTFNLAAKEEYEKSSD
SSEKVTLTIRNLYKEANPDTNVFATQGAYYTATPSDNATTIEKVNVYLNYTGPAIMLN
ADLPTVGGQDNTSINGTSNVDDAFNTAFRGNPSSGLLFTNRYANPLLQSVINYVNKFD
PKFRAEFVTNSINGVTITKVEKTKELRPGTLDDLLKNRNNVFLQQMKNDTEAVYLPVT
ALTNDKWLNTVLVRIPLTKFVKPLSVFTAPVVTPPAEEATGGNQTQEARLQPAGYSNS
DSSGSTEQTPPSTT

>mshv_RS03540-328
MPKIVVDDGYVADTNEGSKEADKTTNQQKLQEWFNTSSNLEKLA
EQLTKKLGEDKFKNMVLSNPTITWDEVQFSNRLYLTPKVTFNLAAKEGYEKATGSLET
VTLTIRNLYKAADPNTNLFATQGASSSAAPGNTMKPNDAAVIKNINVYLNYTGPSIVL
DQAVPAVGTANNTSLNGTSNVTGAFNNAFRGKPSSGLLFTNRYPNPLLQSVINYVNKF
DPKYRVAFVTNSIDGVTITKVESGTQLRPGTLDDLNKNNVFLQQIQGDTEAVYFAVTA
IASNNWLNTFLIRIPLTKFVKPLTEFRPTTPTSTSSDTQQQGTAQTQSNQGS

>mshv_RS03545-396
MGEDSAAEGAQEEDAQAPQAPTTPDLASTVSYLKSLNDTLKAAT
DALNGDNPTTKTAYYKADAGRTLYWDGFMPKITLESFTASQTNVEDNKSKLQTWFNTK
ANWSNLEDQLVKKLGSEKFKNVKLTNPQVDWDIVNSNQYRPKVTFDVTQKEGYELQSG
SEFSQSLTMVIRVLYTTQSPNANVLQMQGASASAAPAGSTPSNNAAVIRDVNVYMNYT
GPNIELDAELPQVGNQNNTSINGTSNVDGDFNTKFKNLLVNVVGGNFTQTSLLQAIIN
YVNKFDPKFRAAFVTEKNGVALTSVQSGTQLRIGSLNDFLYNNKGFLQQVNGDSSAVY
FAVNGVTSEGWLNTFLIRIPLTKFVKPLSVFTAPVVTLPAEEATEGSQTQTSTPQTMP

NSSQ

>mshv_RS03550-367
MPKIVVEDYQPAGHGNNKDTNETTNQQKLQNWFNISSNWEKLSE
QLTKKLGADKFKNVTLSSPTVTYETISTGRDVRIPKVTFTVAAKDGYNLTKPTGETKQ
ISLSIRVLYTSQNQTENALRYQGVSFSAAPNGANSVNHAQTIKDVNVYLNYTGPSIVL
DEALPTVGTTPNTSINGTSNVEGTFNDKFKKLLVNVVRGGHAESSLFQAVINYVNKFD
PKFRAVFVTNSRDGVTITKVQSNTQLRPGTLDDLVKNDRNNVFLQQIQGDTEAVYLPV
TAIASNNWLNTVLVRIPLTKFVRPISVLQTPDQQQTQEGGEEATQPQDAGSGTTSTSE
AQSGQSNGPAGSGTGSTQQAQSSPKTGTSSSSN

>mshv_RS03555-327
MPKIVTTALPTTVAPMNREYNESPTSGYEFWKTRNALILTTWFG
QNQDKLSLVADQLTKKLGEEKFKNMTLSNPRITCDEVKFSNKGSNEDYLTPKVTFNLA
AKKGYALAQDSATSVTLTIRVLYKDSNPEVNVFQTQGSSPSAAPKNTKTPNNAEVIKN
VNVYLNYTGPNIELNAEFPKVGSQENTSLNGTSNVTDDFNNKFNKLLIRYKAETSLFQ
TIINYVNKFDPKFPAAFVTNATNGVTITKVESGTQHRPGTLDDLDKNNVFLQQIQGDT
KAVYFAVTPIASNNWLNTVLVRIPLTKFVRPISVLEEQSAQDQTPEALQQS

>mshv_RS03785-378
MPKIVVNGYVADTTGDNEAAHEEANKTNQKKLEAWFTNSANWAG
LEDQLTKKLGADKFKNVVLTTPTVSYEKVTVGKYTWKNPKVTFNIQAKPGYQLTEPTT
NPKQISLTIRVLYSSQESNQNLLTIKGAYPTAAPRNATVNDANVKSKVNVYVNYTGPA
IELNSDIPTVGAAQNTTINGTSKVEDSTLNNAVKALLSGNTTTDNVSRPLMDAITKYI
QTFDPKFKTNVTPSTYTNTFRAWTSWMETRQREMQGNVALVPALVQVELKMPDNLFLQ
QMKNDTEAVYFAVTAIASNGWLNTFLIRIPLTKFVKPLTVFTATPASASTQDSSASNT
SPQTQLKVLNNKTKNMPQNVSAILRRVYAENCNYSSTYYCCTNE

>mshv_RS03615-345
MPKIVVQGYEADGEEKGKATHKEANQKKLEDWFAANQDKLSLLA
DQLTRKLGKDKFKNMVLSTPTITWDEVEFSNKGSNKVYLTPKVTFNLAAKDGYALAEG
STTTVTLTIRVLYKDSNLTRNVLSTQGAYYTAAPKNAIVDDANVKAKVNVYLNYIGPS
IVLDEALPTVGEQENTSINGTSNVTGDFNTKFKKLLVRDNAQTSLLQAIINYVNKFDP
KFRAAFVTSINGVTVASVQNNTQLRIGSLNDFLWNKKGFLQQVNTDTKAVYFAVMGVT
SNGWVNTFLIRIPLTKFVRPISVLEEQTTEEETQESQEGQENQEQGSTTQTNTESSGT
GQSSDLTEAAA

4481
13,306-396

>mshv_RS03420-155
MPKIVVQGYVADTTGSSKDTNENANRPLLQQWFDTPANWEKLSE
QLTKKLGYDKFKNVTLTNPVISYDDVSTGRDVRIPKVTFTVAAKDGYNLTEPTGETKQ
ITLSIRVLYTSQNQTENALRYQGVAFSAAPSGSSVDDVNVKAKVNVYLNYTDI

>mshv_RS03440-146
MPKIVLEGFDSRWTQNSENQRKIREWFREASNWAGLSDQLTKKL
GAERFKNVKLTFKEASFSGDDIKTPTVKFTVKAKEGYTLTTGDNASATEISLVVRVLY
NRGSENVIQLPRQGSSPSAAPSNSSVDDANVKAKVNVYLNYTDI

>mshv_RS03460-155
MPKIVVQGYVADTTGSSKDTNENANRPLLQQWFDTPANWEKLSE
QLTKKLGYDKFKNVTLTNPVISYDDVSTGRDVRIPKVTFTVAAKDGYNLTEPTGETKQ
ITLSIRVLYTSQNQTENALRYQGVAFSAAPSGSSVDDVNVKAKVNVYLNYTDI

>mshv_RS03480-146
MPKIVLEGFDSRWTQNSENQRKIREWFREASNWAGLSDQLTKKL
GAERFKNVKLTFKEASFSGDDIKTPTVKFTVKAKEGYTLTTGDNASATKISLVVRVLY
NRGSENVIQLPRQGSSPSAAPSNSSVDDANVKAKVNVYLNYTDI

>mshv_RS03495-142
MPKIVLEGFDSTWGRNSDNQDKIRAWFGDAANWAGLSDQLTKKL
GAERFKNVKLTFKEASFSTNAVKTPTVKFTVEAKDGYTLDNSANEISLVIRVLYNRGS
ENVIQLPTQGASPSAAPSNSSVDDANVKAKVNVYLNYTDI

>mshv_RS03535-148
MPKIVLEGFDSSWTNDLDNQTKIREWFDNAANWAGLSDQLTKKL
GAERFKNVKLTFKNATFVGTNIKTPTVTFTVAAKEGYTLVDSGENASATEISLVVRIL
YNRGSESTIQMPTQGASSSAAPGNPTEPNNATVIKNVNVYLNYTDI

>mshv_RS03800-136
MPKIVLDNFDSSWTNANDSANQNKIKAWFREASNWASLSDQLTK
KLGAERFKNVKLTFKNATFVGTNIKTPTVTFTVAAKEGYTLAKGQAASAQEISLVVRV
LYNRDDESTIQLPTQGASSSAVLIRLQKLTMKMF

1028
7, 136-155

Mycoplasmopsis synoviae strain NCTC10124

>D2839_RS01145-320
MLALYWDGFMPKIVLEGFDSRWTNVNDNKSKLQTWFNTKANWSN
LEDQLVKKLGSEKFKNVVLSQPNVTLETFTWNRQTWGVPTVTFNLAAKEGYEKAKNST
NTIALKIRVVYNSNNTNEILFPIQGASSSAAPSGANTADHAKTIKDVNVYLNYTGPAI
VLDATLPEVGKVDNTTTNGTSNVTDDLNTKFKDLVTSDQRTASLFFQAIQKYVNTFDP
KYPANPVSDQKNGVTWARVENKALKIANLNNPNAVFVQQMKDDSEAVYLPVIGVANNR
WLNTFLIRIPLTKFVKPLTVFTATSASTPTQDSSASNTSPQTTT

>D2839_RS03940-334
MLLIYWDGFMPKIVVDDGYVEDTEENNKENNKNTNQQKLEAWFK
ANQDKFTLVADQLTRKLGSDKFKNVTLTNPQVSYEEVTVNSNTWKNPKVTFNIQTKPG
YQLTEPTTEESQISLSIRVLYSSQESNQNLLTIQGASPTAAPSGSSVDDAKVKAKVNV
YMNYTGPNIVLDADLPTVGGKENTSINGTSNVEGTFNDKFKELLVDNNRVPREFLSTV
INYVNKFDPKFRAQLVTDTNGVAITSVQSKKELRIGNLNDLLYNKKVFLQQVNGDSSA
VYFAVNGVTSEGWLNTFLIRIPLTKFVRPLTAFTAPPASAPTQTDSGTGAQTQPEAQS

>D2839_RS01185-362
MLLIYWDGFMPKIVIDDYVADGYDVANNAENNRAQHEAANRPLL
EQWFKVNQDKLSLVAEQLTKKLGEEKFKNMTLSNPEISWDEVKFSNKVSNKGYLTPKV
TFNLTPKEDYKKASDSSETVTLTIRNLYKEANSDTNVFATQGASSSTTPNNASQANDA
TTIEKVNVYLNYTGSNIELDADLPRVGEQENTSLNGTSNVTDDFNNKFNKLLIRYKAE
SSLFQAIINYVNKFDPKFRAQLVTDINGVTVTKVEKIKELRPGTLDDLLKNRNNVFLQ
QMKNDTKAVYFAVTAIASNGWLNTFLIRIPLTKFVKPLSVFTAPVVTPPAEEATGGSQ
TQEARLQPAGYSNSDSSGSTEQTPPSTT

>D2839_RS01220-322
MLLIYWDGFMPKIVVDDGYVEDTEENNKENNKNTNQQKLEAWFK
ANQDKFTLVADQLTRKLGSDKFKNVTLTNPTISWDEVRFSKGNVTKLYLTPKVTFNLA
AKEGYALAQDSATSVTLTIRVLYKDSNPEVNVFQTQGSSPSATPNGANSANHAQTIKD
VNVYLNYTGPNIELNAELPKVGSQENTSLNGTSNVDGDFNTKFKKLLVNVVKEGHAES
SLFQAIINYVNKFDPKFRAQFVTNSINGVTITKVQNTKELRPGTLDDLVKNDRDNVFL
QQMKNDTEAVYFAVSAIASNGWLNTFLIRIPLTKFVKPLTEFQAQS

>D2839_RS01225-367
MLLIYWDGFMPKIVIDDYVADGYDVANNAENNRAQHEAANRPLL
EQWFKVNQDKLSLVAEQLTKKLGEEKFKNMTLSNPTISWDEVRFSKGNVTKLYLTPKV
TFNLAAKEGYALAQDSATSVTLTIRVLYKDSNPEVNVFQTQGSSPSATPNGANSANHA
QTIKDVNVYLNYTGSNIELDADLPRVGEQENTSLNGTSNVDGDFNTKFKKLLVNVVKE
GHAESSLFQAIINYVNKFDPKFRAQFVTNSINGVTITKVQNTKELRPGTLDDLLKNRN
NVFLQQMKNDTKAVYFAVTAIASNGWLNTFLIRIPLTKFVKPLSVFTAPVVTPPAEEA
TGGSQTQEARLQPAGYSNSDSSGSTEQTPPSTT

>D2839_RS03945-383
MASTASYLKSLSDSLKAETDKLNGDTTENKTAYYKVDTGRTLYW
DGFMPKIVVQGYVADTTGSSKDTNENANRPLLQQWFEANQDKLSLVADQLTKKLGSDK
FKNVVLTSPMVSYEDTSSNTRVPKVTFTLQGKEGYELQTTDGASATLSLSIRVLYTSE
NSTQNVLRYQGASSSAAPNRASNPNNATTIKDVNVYLNYTGPNIELNAEAPTVGSQDN
TSINGTSNVDGDFNTKFKNLLVNVVGGDHAESSLFQAIINYVNKFDPKYRAAFVTNSR
DGVTITKVQNTKELRPGTLDDLLSNKNKVFLQQIKGDTEAVYFAVTAIASNNWLNTFL
IRIPLTKFVRPISVLEEQSAQDQTPEASQQAPEGGSLASGTAPTEGTQS

>D2839_RS01275-349

MLLIYWDGFMPKIVIDDYVADGYDVANNAENNRAQHEAANRPLL
EQWFKVNQDKLSLVAEQLTKKLGEEKFKNMTLSNPEISWDEVKFSNKVSNKGYLTPKV
TFNLTPKEDYKKASDSSETVTLTIRNLYKEANSDTNVFATQGASSSTTPNNASQANDA
TTIEKVNVYLNYTGPNIELDADLPTVGGQENTSLNGTSNVDGAFNTAFRGNPSRGLLF
INRYANPLLKSVINYVNKFDPKFRAQFVTNSINGVTITKVESGTQLRPGTLDDLLKNR
NNVFLQQIKGDTEAVYFAVSAIASNGWLNTFLIRIPLTKFVKPLTEFRPTTPTSPSSD
TQQQGTAQTQSNQGS

>D2839_RS01295-358
MLIKFWDGFMPKIVVQGYEADGEEKGKATHKEANQKKLEDWFAA
NQDKLSLVADQLTRKLGKDKFKNMVLSTPTITWDEVEFSNKGSNKVYLTPKVTFNLAA
KDGYALAEGSTTTVTLTIRVLYKDSNLTRNVLSTQGAYYTAAPKNAIVDDANVKAKVN
VYMNYTGPNIELDADLPTVGGKENTSINGTSNVEGTFNDKFKKLLVNVVGESITQTSL
LQAIINYVNKFDPKFPAEFVLKTNGVTVASVQNNTQLRIGSLNDFLYNNKGFLQQVNT
DTKAVYFAVMGVTSNGWVNTFLIRIPLTKFVRPISVLEEQTTEEETQESQEGQENQEQ
GSTTQTNTESSGTGQSSDLTEVSA

>D2839_RS03970-354
MLALYWDGFMPKIVVEGYVASDDARVDEKTHEDANTKLLQQWFD
TPANWEKLSEQLTKKLGADKFKNVTLSSPKVTYETISTGKNVKIPKVTFTLQGKEGYN
LQIDNGASQSLTLLIRVLYTSADATENALRYQGASSSTAPGSPTVNDANVKAKVNVYL
NYTGPSIVLDEALPTVGGKENTLINGTSNVEGTFNDKFKKLLVNVVGENITQTSLLQA
IINYVNKFDPKFPASFVLKTNGVAIASVQNNTQLRIGSLNDFLWNKKGFLQQVNGDSS
AVYFAVNGVTSQGWLNTFLIRIPLTKFVKPLTEFRPTTHTSPSSDTQQEQTEQTGGNQ
DASGGASESGTASQPQQAAS

>D2839_RS03975-360
MLSLYWDGFMPKITLESFTASQTNVEDNKSKLQKWFNTKANWSN
LEDQLVKKLGSKKFKNVKLTNPQVDWDIVNSNQYRPKVTFDVTQKEGYELQRGSEFSQ
SLTMVIRVLYTTQSPNANVLQMQGASSLAVPNDTTTVSDANVLRTVNVYLNYTGPNIV
LDADLPTVGGQENTSINGTSNVTGAFNNAFRGNPTSGLLFTNRYPNPLLKSVINYVNK
FDPKFKAEFVTNSKNGVTITKVEKNKKQKREELRPGTLDDLLSNKDIVFLQQIKGDTE
AVYFAVSAITNDRWLNTVLVRIPLTKFVRPISVLEAPQQEETQEDGEEMQQGNQDGTQ
AGQTDETAGSGTPQASSPQTMPNSSQ

>D2839_RS01465-328
MLALYWDGFMPKIVVPTYEPDTTDDNKAAHEEANKTKLEAWFTN
SANWAGLEDQLTKRLGSDKFKNVKLTYASVTLTDFTAGRGVTRWNPTVKFSVKANEGY
TLDTNSTKEIDLVVRVLYKSAEANAANVFTHEGASPSAAPKNTKTPNDPKVIKNVNVY
LNYTGPNIELDAEAPTVGSQDNTSINGTSNVDDAFNTAFRGNPSKGLLFTNRYANPLL
KSVINYVNKFDPKFRAEFVTDARDGVTITKVEKTKELRPGTLDDLVKNRNNVFLQQIK
GDKEAVYFAVTALTNDKWLNTVLVRIPLTKFIRSVAPFAAAAPAEPNSDTQE

>D2839_RS01475-331

MLIKFWDGFMPKIVLTDFDKTWEQNSEENERKIREWFREASNWA
GLSDQLTKKLGAERFKNVKLTFKEASFSGENNIKTPTVTFTVAAKDGYTLVDSGENAS
ATEISLVVRVLYNRGSENVIQLPYQGASSSAVPNKTTKVNDENVLRTVNVYLNYTGPS
IVLDADLPQVGAATNTSLNGTSNVEGTFNTAFRGNPTSGLLFTNRYANPLLKSVINYV
NKFDPKYRAEFVTDARDGVTITKVQNTRELRPGTLDDLDKDNVFLQQIKGDKEAVYFA
VTAVASNRWLNTFLIRIPLTKFVKPLTEFRPTTPTNPSSDTQQQGTAQTQSNQGS

>D2839_RS01480-337
MPKIVVEGYVADGEGNGKATHETTNQQKLQQWFATESNWEKLSE
QLTKKLGADKFKNVVLTSPMVSYEEVTVNTNTWKNPKVTFNIQAKPGYQLTEPTTEES
QISLSIRVLYSSQESNQNLLTIQGASPTAAPNNTRNANDETTKKTVNVYLNYTGPSIV
LDQAVPAVGTANNTSLNGTSNVTGDFNTKFKKLLVSVLRRGHAESSLFQAIINYVNKF
DPKFRAQFVTNSIDGVTITKVQNTKELRPGTLDDLVKNDRDNVFLQQMKNDTEAVYLP
VTALTNDKWLNTVLVRIPLTKFVRPLTEFQAQSEAAPTEEASGDQAQADPQATQMGTQ
AQS

>D2839_RS03865-342
MLLIYWDGFMPKIVLDNFDSGWTNDLDNQNKIKAWFGEAENWEG
LSDQLTKKLGTERFKNVVLSNPTISVETVTLSSGSVKFPKVTFRVAAKDGYQLASGEG
TTTTLELKIRVLYSSSNPNTLLLPYQGASPSAAPAGSTPSNNAAVIRDVNVYMNYTGP
NIELDADLPRVGEQENTSINGTLNVDGDFNTKFKKLLVNVVGESITQTSLLQAIINYV
NKFDPKFPAEFVLKTNGVALTSVQSGTQLKIGSLNDFLYNNKGFLQQVNGDSSAVYFA
VTAIASNNWLNTFLIRIPLTKFVKPVTAFMPAAPIAPAQQESQENTQTTQGSQQAPEG
GSSTSGTG

4847
14,320-383

>D2839_RS04365-196
MLNLYWDGFMPKIVLEGFDKTWEQNSEENERKIRKWFDNAANWV
GLSDQLTKKLGAERFKNVKLTYKEVTFSTNAVKTPTVTFTVEAQDGYTLDNSANEISL
VVRVLYNRGSENVIQLPTQGASSSAAPAGSTPSNNAAVIRDVNVYMNYTGPALILNAA
LPTVGNAANTSLNGTSNVTDEDFNAALEDCYLPIDM

>D2839_RS01355-156
MLALYWDGFMPKIVLTDFDKTWGQNSEENERKIRKWFDNAANWA
SLSEQLTKKLGAERFKNVKLTFKSARFSTDPAKVPTVTFTVAAKEGYTLTTGDNASAT
EISLVVRVLYNRESESTIQMPTQGASSSAAPRGATVNDAKAKAKVNVYLNYTDI

>D2839_RS01405-157
MLALYWDGFMPKIVVNDYKIVVDGYVTNTDEVKKEADKTTNQEK
LQNWFNDPANWKKLAEQLTKKLGEEKFKNMVLSTPTITWDEVEFSGTHYLTPKATFNL
ATKEGYALAQDSANTVTLTIRVLCKDSNPEVNVFQTQGSSPSAAPSNSSVNDANVKAK
VNVYLNYTDI

>D2839_RS01510-151
MLLIYWDGFMPKIVLEGFDSTWGRNSDNQDKIRAWFGDAANWVG
LSDQLTKKLGAETFKNVKLTYKEVTFSTNAVKTPTVTFTVEAQDGYTLDNSANEISLV
VRVLYNRGSENVIQLPRQGASSSAAPRGATVNDANVKAKVNVYLNYTDI

660
4,151-196

Mycoplasmopsis synoviae strain WF18

>JM201_RS01165-331
MPKIVVPTYEPDTTDDNKAAHEEANKTKLEAWFTNSANWAGLED
QLTKRLGSDKFKNVKLTYASLTLTDFTASRGVTRWNPTVKFSIKANEGYTLDTNSTKE
IDLVVRVLYKSAEANAANVFTHEGASSSAAPKNTMTPNNAEVIKNVNVYLNYTGPSIV
LDEALPTVGGKENTSINGTSNVDGDFNTAFRGNPSSGLLFTNRYANPLLKSVINYVNK
FDPKYRAEFVTNSKNGVTISNVTNNVQLRPGTLDDLLSNKNKVFLQQIKGDTKAVYFA
VTAIASNNWLNTFLIRIPLTKFVKPLTEFRPTTPTSTSSDTQQQGTAQTQSNQGS

>JM201_RS01170-314
MTKIVLEGFDSRWTNVNDNKSKLQTWFNTKANWSNLEDQLVKKL
GSEKFKNVVLSQPNVTLETFTWNRQTWGVPTVTFNLAAKEGYEKAKNSTNTIALKIRV
VYNSNNTNEILFPIQGASSSAAPSGANTADHAKTIKDVNVYLNYTGPAIVLDATLPVV
GKVDNTTTNGTSNVTDDLNTKFKDLVTSDQRTASLFFQAIQKYVNTFDPKYPANSISD
QKNGVTWARVENKALKIANLNNPNAVFVQQMKDDSEAVYLPVIGVANNRWLNTFLIRI
PLTKFVKPLTVFTATPTWAPTQDSSASNTSPQTTTQSS

>JM201_RS01175-361
MPKIVIDDYVADGYDVANNAENNRAQHEAANRPLLEQWFKVNQD
KLSLVAEQLTKKLGEEKFKNMTLSNPEISWDEVKFSNKVSNKGYLTPKVTFNLTPKED
YKKASDSSEKVTLTIRNLYKEANSDTNVFATQGAYYTATPSDNATTIKKVNVYLNYTG
PNIELDADLPRVGEQENTSLNGTSNVTDDFNNKFNKLLIRYKAETSLFQTIINYVNKF
DPKFPAKFVTNSINGVTITKVQSDTQLRPGTLDDLLKNRNNVFLQQMKNDTKAVYFAV
NGVTSQGWLNTLLIRIPLTKFVRPISVFTAPVVTPPTQDDSQEDSSNAGNTNENTETP
VEDQTGQESSESRSTQTDQAQQESPQT

>JM201_RS01180-347
MPKIVVEDYVSDGDGNNRAQHEAANQQKLQEWFNTSSNWEKLSE
QLTKKLGADKFKNVTLTNPQVSYEEVTVNSNAWKNPKVTFNIQAKPGYQLTEPTTEES
QISLSIRVLYSSQESNQNLLTIQGASPTAAPNNTTMVSDPNVLRTVNVYLNYTGPNIE
LDAELPQVGNQNNTSINGTSNVEGTFNDKFKKLLVNVVGESITQTSLLQAIINYVNKF
DPKFPASFVLKTNGVAIASVQNNTQLRIGSLNDFLYNNKGFLQQVNTDTKAVYFAVMG
VTSNGWVNTFLIRIPLTKFVRPISVLEEQTTEEETQESQEGQENQEQGSTTQTNTESS
GTGQSSDLTEVSA

>JM201_RS01185-317
MPKIVLEGFDSTWGRNSDNQDKIRAWFGDAANWAGLSEQLTKKL
GAERFKNVVLSNPQIIFEDVMLSSGSVKFPKVTFTVAAKDGYQLASGEGTSTTLELKI
RVLYSSSDPNALLLPYQGASSSAAPRGATVNDPNVKSKVNVYLNYTGPNIVLDADLPT
VGGKENTSINGTSNVEGTFNDKFKELLVDNNRVPREFLSTVINYVNKFDPKFRAQLVT
DTNGVALTSVQNGIQLRIGNLNDLLYNKKVFLQQVHGDSSAVYFAVTAIASNNWLNTF
LIRIPLTKFVRPLTAFTATPASAPTQTDSGTGAQTQPEAQS

>JM201_RS01210-246
MPKIVLTDFDKTWGQNTANQDKIRAWFGDAANWAGLSDQLTKKL
GAERFKNVVLSNPQIIFEDVRLLTRGSVKFPKVTFTVAAKDGYQLASGEGTSTTLELK
IRVLYSSSDPNALLLPYQGASSSAAPRGATVNDPNVKSKVNVYLNYTGPSIVLDQVVP
AVGTANNTSLNGTSNVTGDFNTKFKKLLVNVVRQGHAESSLFQAVINYVNKFDPKFRA
QFVTNSINGVTITKVQKDKQKRDRELRL

>JM201_RS01240-357
MPKIVVEDYQPAGHGNNKDTNETTNQQKVQEWFNTSSNWEKLAE
QLTKKLGANKFKNVVLTAPTVSYEDGGSNTRIPKVTFTLHGKDGYTLQTDNGATSSLT
LSIRVLYTSASSTQNALRYQGASSSAAPSGSTPSNNAAVIRNVNVYMNYTGPNIELDT
ELPQVGNQNNTSINGTSNVDGDFNTKFKNLLVNVVGGSFIQTSLLQAIINYVNKFDPK
FRAAFVTSINGVALTSVQNDTQLRIGSLNDFLYNNKGFLQQVNGDSSAVYFAVNGVTS
EGWLNTFLIRIPLTKFVRPITPFTASAVMIPTTEDYQEDSSNAGNTNENKETPVEDQT
GQESSESGSTQTDQAQQESPQTQ

>JM201_RS01245-326
MPKIVVQGYQADGAGNGKATHEAANRPLLQQWFETPANWEKLSE
QLTKKLGSDKFKNVVLTAPTVSYEEVTVNSNTWKNPKVTFNIQAKPGYQLTQPTTDPK
QISLTIRVLYSSQESNQNLLTIQGASPTAAPSNSSVDDANVKAKVNVYLNYTGPNIVL
DADLPTVGGKENTSINGTSNVEGTFNTKFKNLLVNVVGGSFTQTSLFQAIINYVNKFD
PKFRAAFVTSINGVALTSVQSGTQLRIGNLNDFLYNKKVFLQQVHGDSSAVYFAVTAI
ASNNWLNTFLIRIPLTKFVRPLTAFTATPASAPTQTDSGTGAQTQPEAQS

>JM201_RS01270-374
MPKIVVSLLTTKAANQEYDASVRQGGVVFWKKRNGEILDVWFAA
NQDKFTLVADQLTKKLGEEKFKNMTLSNPTITWDELTIPEGTILTPKVTFTLTPKDGY
TLADNSENTVTLTIRNLYKAADPNTNLFATQGASPSAAPKNTKTPNDAEVIKNVNVYL
NYTGPSIVLDQDLPTVGGQENTSINGTSNVTGDFNNAFRGNPSSGLLFTNRYPNPLLK
SVINYVNKFDPKFRAEFVTNATNGVTITKVQSGTQLRPGTLDDLNKNNVFLQQIKGDT
EAVYFAVSAIASNGWLNTFLIRIPLTKFVRPISVLQTPDQQQTQEGGEEATQPQNAGS
GTASTSEVQSGQSNGAVGSGTGSTQQAQSSSTTGTSSSSN

>JM201_RS01290-359
MPKIVVDDGYVEDTEENNKENNKNTNQQKLEAWFAANQDKFTLV

ADQLTRKLGEEKFKNMTLSNPTISWDESVWKTKTILTPKVTFNLTPKEGYEKATGSLE
TVTLTIRNLYKEANPDTNVFATQGASSSATPNNASQANDATTIEKVNVYLNYTGPSIV
LDEALPTVGATPNTSINGTSNVTGDFNTKFKKLLVSGTTQTPLLQTIINYVNKFDPKF
PASFVLKTNGVAIASVQNNTQLKIGSLNDFLWNNKGFLQQVNTDTKAVYFAVIGVASN
GWLNTFLIRIPLTKFVRSITPFTAPAVMTPTTEDSQEESRENSSQADNTEQNAENQAD
GQSGGEATETQEAQGDSASQSSPQT

>JM201_RS01295-298
MHKIVVPTYEPDTTSDSKATHETTNQQKLQNWFNAPANWEKLAE
QLTKKLGADKFKNVTLTNPIVTYEEITVDKYTWKNPKVTFAVEAKPGYQLASGAGTST
TLELKIRVLYTSESSTQNLLTIQEASSSAAPSGANTADHAKTIKDVNVYLNYTGPAIV
LDTTLPEVGNANNTIINGTSPITDETLAAKVKTLFNKTTNTNDPTELMLAITKYVQTF
DPKYLSTRTDNGYRAWVSWMWTNRSALSQRELKQPADIFLQQMKDDTEAVYLPIGGTT
DDKWLNTFLIRIPLNKFVKAIL

>JM201_RS01310-342
MPKIVLTDFDKTWGQNTANQTKIRDWFREASNWEGLSDQLTKKL
GVERFKNVVLSQPNVTFEIINWNGQTWRVPTVTFNLAAKEGYELIGSTNTIALKIRVV
YDSNDANAILFPIQGASSSAVPNRASNPNDATTIKNVNVYLNYTGPAIVLNADLPTVG
GQENTSINGTSNVDGTFNTAFRGNPSSGLLFTHRYANPLLKSVINYVNKFDPKFRAEF
VTDSKNGVTISNVTNNVQLRPGTLDDLLSNKNKVFLQQIKGDTEAVYFAVNGVANNRW
LNTFLIRIPLTKFVRPLTEFQAQSATVPTQEGQTTQGQENSAGSGALGSTTGTQTTSG
GSSQASGS

>JM201_RS01330-340
MPKIVVDDGYVEDTEENNKENNKNTNQKKLEAWFKANQDKFTLV
ADQLTRKLGEEKFKNMTLSNPTISWDEVKFSKGNVTKLYLTPKVTFNLAAKEEYEKSS
DSLETVTLTIRNLYKETNPDTNVFATQGASSSATPNNASQANDATAIEKVNVYLNYTG
PSIVLDEALPTVGATPNTSLNGTSNVTGEFNTKFRKLLVSVFRQGHAESSLFQAVINY
VNKFDPKYRATFVTNSINGVTITKVQNTKELRPGTLDDLVKNDRNNVFLQQIQRDTEA
VYLPVTAVTIDKWLNTVLVRIPLTKFVRSITPFTAPVVTPPAEEATEGSQTQASTPQT
MPNSSQ

>JM201_RS01350-331
MHKIVVPTYEPDTTSDSKATHETTNQQKLQNWFNAPANWEKLSE
QLTKKLGADKFKNVKLTYKSITLTDFTVDRATRWYPTVKFSVEANEGYTLNTNSTKEI
DLVVRVLYKSAEANAANVFTHEGASSSAAPKNTKTPNNAEVIKNVNVYLNYTGPSIVL
DVAHPTVGGQENTSINGTSNVDGAFNTAFRENPSKGLLFTNRYANPLLKSVINYYVNKF
DPKYRAAFVTSINGVTITKVQNTKELRPGTLDDLNKNNVFLQQIKGDKEAVYFAVSTI
ASNNWLNTFLIRIPLTKFIKPLSVFTAPVVTPPAEEATETQEAQGDSASQSSLQT

>JM201_RS01385-324
MPKIVVQGYQADGAGVNKAQHEEANKTKLQQWFATESNWEKLSE
QLTKKLGSDKFKNVVLTAPTVSYEDNGSNTRIPKVTFTLQGKDGYILQTDNGATSSLT

LSIRVLYTTQSPNANVLQMQGASPTAAPGNTMKPNDDAVIKNVNVYLNYTGPSIVLDV
AHPTVGGQENTSINGTSNVDGAFNTAFRENPSRGLLFTNRYANPFLKSVINYVNKFDP
KYRAAFVTEKNGVAIASVQSGTQLKIGSLNDFLYNNKGFLQQVHGDSTAVYFAVTAIA
SNNWLNTFLIRIPLTKFVKPLIAFTATPASAPTQTDSGTASQPQQAAS

>JM201_RS01390-330
MPKIVVEGYEADGEGNGKATHEDANKALLQQWFDTPANWEKLSE
QLTKKLGSDKFKNVTLANPVISYDDVSTGRDVRIPKVTFTVAAKDGYNLTEPTGNTKQ
ISLSIRVLYTSATSTQNALRYQGVAFSGAPSGSLVDDANVKAKVNVYMNYTGPAIVLD
QAVPAVGTATNTSLNGTSNVTGDFNNKFNKLLIRYKAETSLFQTIINYVNKFDPKFRA
QFVTNSINGVTITKVQNTKELRPGTLDDLVKNRNNVFLQQVHGDSSAVYFAVTALTND
KWLNTVLVRIPLTKFVRPISVLEEQSAQDQTPEASQQADGDQSTSSSPQGTAQT

>JM201_RS01400-298
MPKIVLTDFDKTWAQNSENEGKIREWFATVSNWATLSDQLTKKL
GAERFKNVKLTFKEASFSSDNIKTPTVTFTVAAQDGYTLDNSTNEISLVVRVLYNRDN
ESTIQLPTQGDSSSAAPAGSTPSNNAAVIRDVNVYMNYTGPALILNAALPTVGNAANT
SLNGTSNVTDEDFNAAFRGLLFTNRYVNPLMQSVINYVNKFDPKYRAMFVTNAIDGAA
LTKVQNKKELRPGNLEDLLYNNNVFLQQMKNDTEAVYFAVTALTIDKWLNTVLVRILL
TKFVRPLTEFQAQSSPTTGTSS

5595
16,246-374

>JM201_RS01160-160
MPKIVVDDGYVENTEENNKENNKNTNQQKLEAWFKANQDKFTLV
ADQLTRKLGEEKFKNMTLSNPTISWDEVKFSKGNVTKLYLTPKVTFNLAAKEGYEKAS
DSSETVTLTIINLYKEANPDTNVFATQGASSSAAPRGATVNDANVKAKVNVYLNYTDI

>JM201_RS01235-148
MPKIVLEGFDSTWGRNSENQTKIREWFGNAANWEGLSDQLTKKL
GAERFKNVKLTYKEVTFSTNAVKTPTVTFTVAAKDGYTLVDSGKNASSTEISLVVRVL
YNRDNESIIQLPTQGASSSAAPNNAKNANDKTTKRTVNVYLNYTDI

>JM201_RS01255-148
MPKIVLTDFDKTWEQNSEENERKIRKWFDNAANWVGLSDQLTKK
LGAERFKNVKLTYKEVTFSTNAIKTPTVKFTVAAKEGYTLTTGDNASGTEISLVVRVL
YNRGSENVIQLPTQGASSSAAPNNAKNANDKTTKRTVNVYLNYTDI

>JM201_RS01265-148
MPKIVLTDFDKTWEQNSENERKIREWFREAANWATLSDQLTKKL
GAERFKNVKLTFKEANFSISSDNIKTPTVKFTVKAKDGYTLTTGDNASATEISLVVRV
LYNHDDESTIQLPTQGASPSAAPSGSSVNDAKVKAKVNVYLNYTDI

>JM201_RS01305-147
MPKIVLTDFDKTWGQNSEENERKIREWFREASNWAGLSDQLTKK
LGAERFKNVKLTYKEVTFSTNAVKTPTVTFTVAAKDGYTLTTGDNASATEISLVVRVL
YNHDDESTIQLPTQGASPSAAPSGSSVNDAKVKAKVNVYLNYTDI

>JM201_RS01325-153
MPKIVLEGFDSTWRENSEENERKIREWFREASNWAGLSDQLTKK
LGAERFKNVKLTFKEASFSSDNIKTPTVTFTVAAKDGYTLTTGDNASATEISLVVRVL
YNHDDESTIQLPTQGASSSAAPNTASANNPALAIKNVNVYLNYINQFKLTN

>JM201_RS01340-152
MPKIVLEGFDSTWRENSANQDKIRAWFGDAANWAGLSDQLTKKL
GAERFKNVKLTFKEASFSSDNFKTPTVTFTVAAKDGYTLTTGDNASATEISLVVRVLY
NHDDESTIQLPTQGASSSAAPNTASANNPALAIKNVNVYLNYINQFKLTN

>JM201_RS01365-157
MPKIVVDDGYVEDTEENNKENNKNTNQKKLEAWFGDAANWAGLE
DQLTKRLGSDKFKNVKLTYTSVTLTDFTAGRGVTRWNPTVKFSVKANEGYTLDTNSTK
EIDLVVRVLYKSAEANAANVFTHEGASSSATSRGATFNDPNVKSKVNVYLNYTDI

>JM201_RS01370-149
MPRIVLSPLPEEVARANKEYDASIRQGGVVFWKTRNGEILDVWF
AANQDKFTLVADQLTRKLGEEKFKNMTLSNPTISWDEVKFSKGNVTKLYLTPKVTFNL
AAKEGYEKATDSLEKVTLTIRNLYKEANPDTNVFATQGTASSSVSEV

>JM201_RS01380-156
MPKIVVSGYEADGAGVNKAQHEEANKTKLQQWFATESNWEKLSE
QLTKKLGADKFKNVKLTYKSITLTDFTVDRATRWYPTVKFSVEANEGYTLNTNSTKEI
DLVVRVLYKSAEANAANVFTHEGASSSATPNNAKNANDKTTKRTVNVYLNYTDI

>JM201_RS01410-166
MPKIVTTALPTTVAPMNREYNESPTGGHEFWKGRNKQVLDAWFA
ANQDKLSLVAEQLTKKLGKDKFKNMVLSTPTITWDEVEFSGRRYLTSKVTFTLTPKDG
YEKVSDSSETVTLTIRNLYKEANPDTNIFATQGTSSSAAPDNTTEPNNATVIKNVNVY
LNYTDI

1684
11,147-166
