# Supplementary material for: Residues Ser83, Arg85, Tyr88, Asn124, and Lys192 of C-Terminal Lipid-Associated Membrane Hemagglutinin Affect Mycoplasma synoviae Agglutination of Erythrocytes
Source: Microorganisms. 2026 Sep 12;14(9):2031. doi: 10.3390/microorganisms14092031 (PMC13609628; doi:10.3390/microorganisms14092031)
Supplement: Supplementary file 1 [file microorganisms-14-02031-s001.zip › Supplementary Data S3-Conserved structural features of long- and short-chain hemagglutinins.pdf]

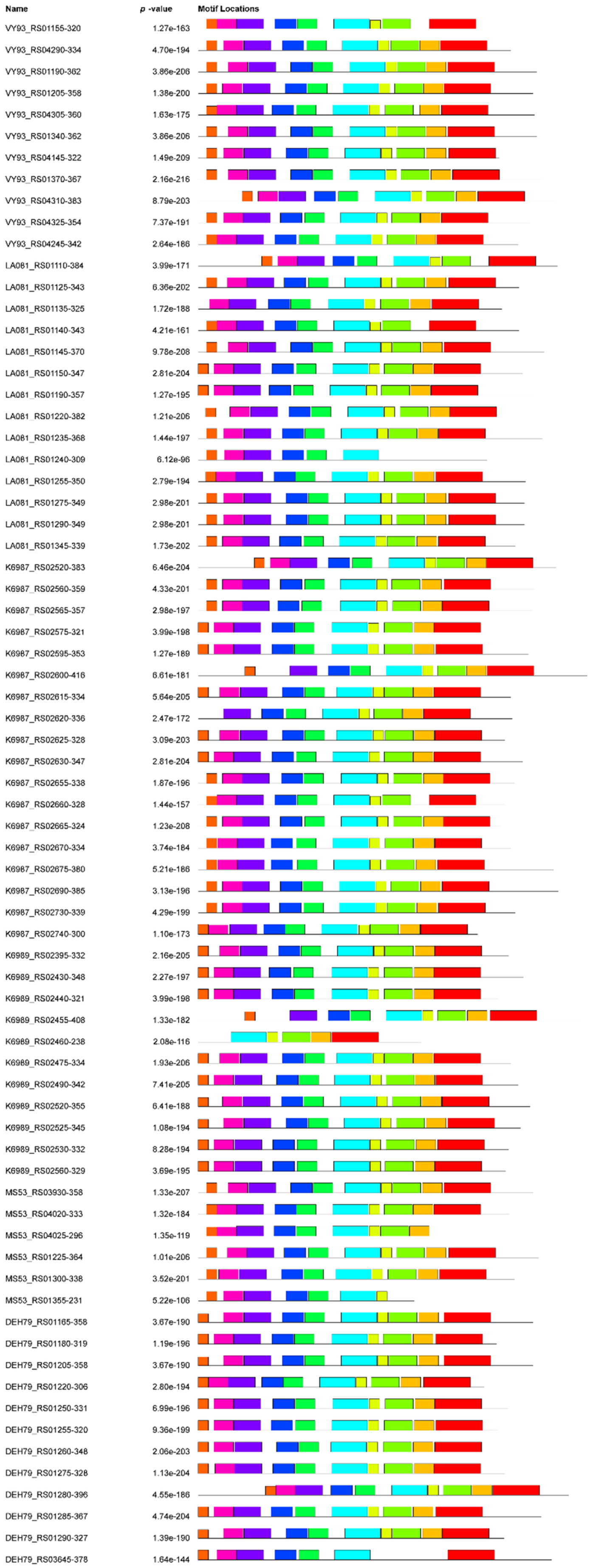
Name
p -value
Motif Locations
VY93_RS01155-320 1.27e-163
VY93_RS04290-334 4.70e-194
VY93_RS01190-362 3.86e-206
VY93_RS01205-358 1.38e-200
VY93_RS04305-360 1.63e-175
VY93_RS01340-362 3.86e-206
VY93_RS04145-322 1.49e-209
VY93_RS01370-367 2.16e-216
VY93_RS04310-383 8.79e-203
VY93_RS04325-354 7.37e-191
VY93_RS04245-342 2.64e-186
LA081_RS01110-384 3.99e-171
LA081_RS01125-343 6.36e-202
LA081_RS01135-325 1.72e-188
LA081_RS01140-343 4.21e-161
LA081_RS01145-370 9.78e-208
LA081_RS01150-347 2.81e-204
LA081_RS01190-357 1.27e-195
LA081_RS01220-382 1.21e-206
LA081_RS01235-368 1.44e-197
LA081_RS01240-309 6.12e-96
LA081_RS01255-350 2.79e-194
LA081_RS01275-349 2.98e-201
LA081_RS01290-349 2.98e-201
LA081_RS01345-339 1.73e-202
K6987_RS02520-383 6.46e-204
K6987_RS02560-359 4.33e-201
K6987_RS02565-357 2.98e-197
K6987_RS02575-321 3.99e-198
K6987_RS02595-353 1.27e-189
K6987_RS02600-416 6.61e-181
K6987_RS02615-334 5.64e-205
K6987_RS02620-336 2.47e-172
K6987_RS02625-328 3.09e-203
K6987_RS02630-347 2.81e-204
K6987_RS02655-338 1.87e-196
K6987_RS02660-328 1.44e-157
K6987_RS02665-324 1.23e-208
K6987_RS02670-334 3.74e-184
K6987_RS02675-380 5.21e-186
K6987_RS02690-385 3.13e-196
K6987_RS02730-339 4.29e-199
K6987_RS02740-300 1.10e-173
K6989_RS02395-332 2.16e-205
K6989_RS02430-348 2.27e-197
K6989_RS02440-321 3.99e-198
K6989_RS02455-408 1.33e-182
K6989_RS02460-238 2.08e-116
K6989_RS02475-334 1.93e-206
K6989_RS02490-342 7.41e-205
K6989_RS02520-355 6.41e-188
K6989_RS02525-345 1.08e-194
K6989_RS02530-332 8.28e-194
K6989_RS02560-329 3.69e-195
MS53_RS03930-358 1.33e-207
MS53_RS04020-333 1.32e-184
MS53_RS04025-296 1.35e-119
MS53_RS01225-364 1.01e-206
MS53_RS01300-338 3.52e-201
MS53_RS01355-231 5.22e-106
DEH79_RS01165-358 3.67e-190
DEH79_RS01180-319 1.19e-196
DEH79_RS01205-358 3.67e-190
DEH79_RS01220-306 2.80e-194
DEH79_RS01250-331 6.99e-196
DEH79_RS01255-320 9.36e-199
DEH79_RS01260-348 2.06e-203
DEH79_RS01275-328 1.13e-204
DEH79_RS01280-396 4.55e-186
DEH79_RS01285-367 4.74e-204
DEH79_RS01290-327 1.39e-190
DEH79_RS03645-378 1.64e-144

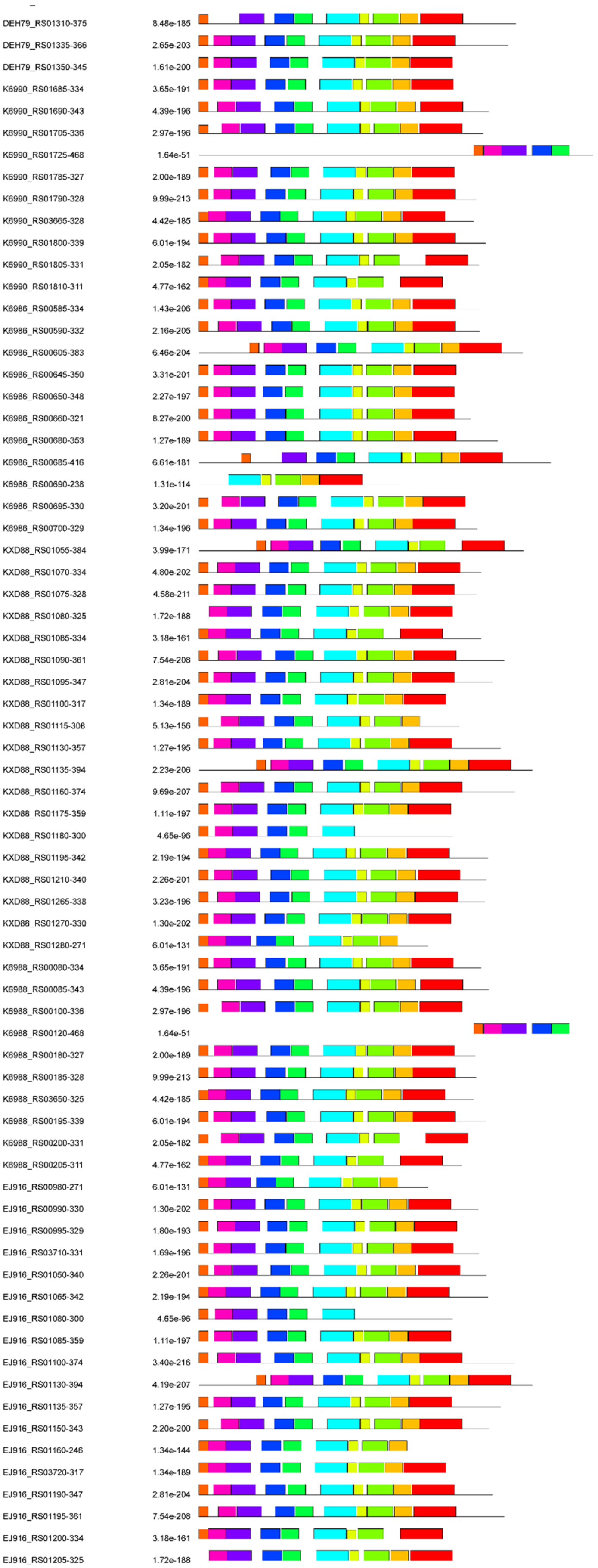
DEH79_RS01310-375 8.48e-185
DEH79_RS01335-366 2.65e-203
DEH79_RS01350-345 1.61e-200
K6990_RS01685-334 3.65e-191
K6990_RS01690-343 4.39e-196
K6990_RS01705-336 2.97e-196
K6990_RS01725-468 1.64e-51
K6990_RS01785-327 2.00e-189
K6990_RS01790-328 9.99e-213
K6990_RS03665-328 4.42e-185
K6990_RS01800-339 6.01e-194
K6990_RS01805-331 2.05e-182
K6990_RS01810-311 4.77e-162
K6986_RS00585-334 1.43e-206
K6986_RS00590-332 2.16e-205
K6986_RS00605-383 6.46e-204
K6986_RS00645-350 3.31e-201
K6986_RS00650-348 2.27e-197
K6986_RS00660-321 8.27e-200
K6986_RS00680-353 1.27e-189
K6986_RS00685-416 6.61e-181
K6986_RS00690-238 1.31e-114
K6986_RS00695-330 3.20e-201
K6986_RS00700-329 1.34e-196
KXD88_RS01055-384 3.99e-171
KXD88_RS01070-334 4.80e-202
KXD88_RS01075-328 4.58e-211
KXD88_RS01080-325 1.72e-188
KXD88_RS01085-334 3.18e-161
KXD88_RS01090-361 7.54e-208
KXD88_RS01095-347 2.81e-204
KXD88_RS01100-317 1.34e-189
KXD88_RS01115-308 5.13e-156
KXD88_RS01130-357 1.27e-195
KXD88_RS01135-394 2.23e-206
KXD88_RS01160-374 9.69e-207
KXD88_RS01175-359 1.11e-197
KXD88_RS01180-300 4.65e-96
KXD88_RS01195-342 2.19e-194
KXD88_RS01210-340 2.26e-201
KXD88_RS01265-338 3.23e-196
KXD88_RS01270-330 1.30e-202
KXD88_RS01280-271 6.01e-131
K6988_RS00080-334 3.65e-191
K6988_RS00085-343 4.39e-196
K6988_RS00100-336 2.97e-196
K6988_RS00120-468 1.64e-51
K6988_RS00180-327 2.00e-189
K6988_RS00185-328 9.99e-213
K6988_RS03650-325 4.42e-185
K6988_RS00195-339 6.01e-194
K6988_RS00200-331 2.05e-182
K6988_RS00205-311 4.77e-162
EJ916_RS00980-271 6.01e-131
EJ916_RS00990-330 1.30e-202
EJ916_RS00995-329 1.80e-193
EJ916_RS03710-331 1.69e-196
EJ916_RS01050-340 2.26e-201
EJ916_RS01065-342 2.19e-194
EJ916_RS01080-300 4.65e-96
EJ916_RS01085-359 1.11e-197
EJ916_RS01100-374 3.40e-216
EJ916_RS01130-394 4.19e-207
EJ916_RS01135-357 1.27e-195
EJ916_RS01150-343 2.20e-200
EJ916_RS01160-246 1.34e-144
EJ916_RS03720-317 1.34e-189
EJ916_RS01190-347 2.81e-204
EJ916_RS01195-361 7.54e-208
EJ916_RS01200-334 3.18e-161
EJ916_RS01205-325 1.72e-188

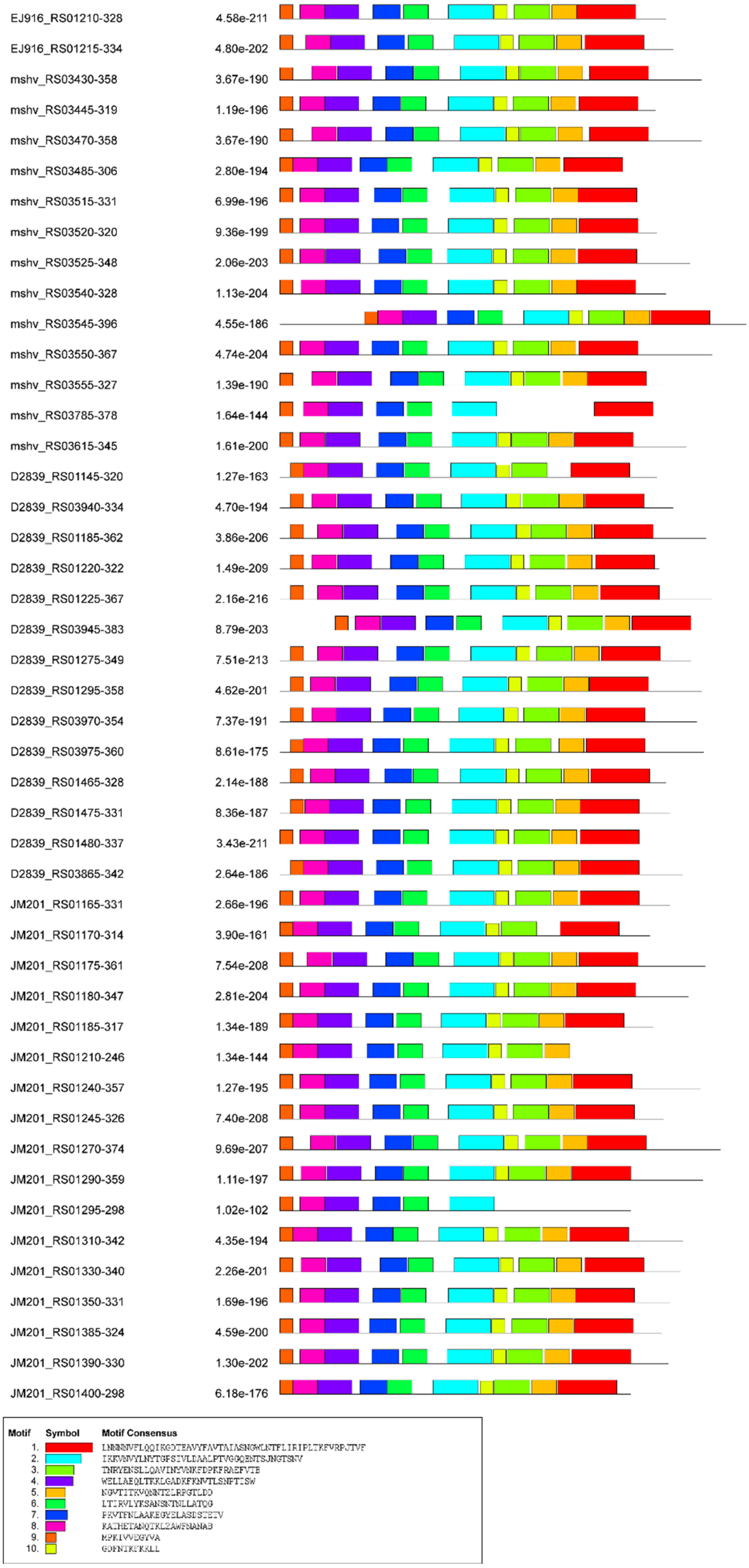

Figure S1. Long chain discovered motif and motif locations.

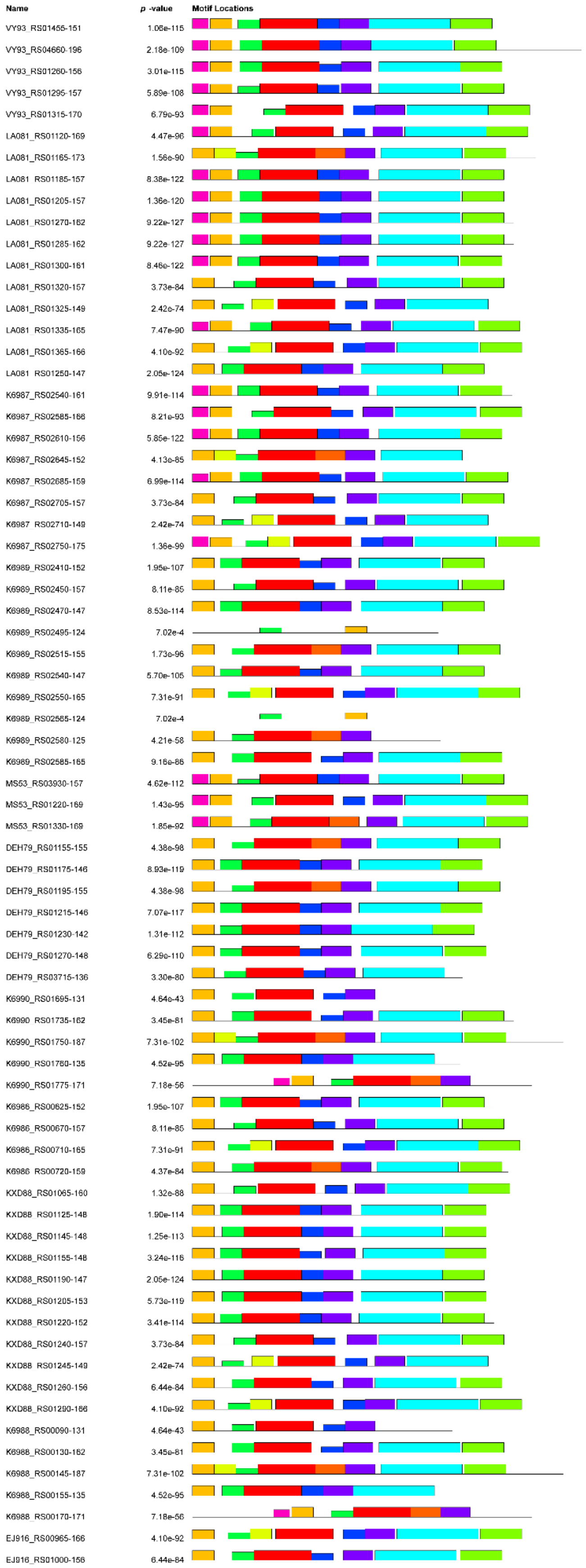

Name
p -value
Motif Locations
VY93_RS01455-151 1.06e-115
VY93_RS04660-196 2.18e-109
VY93_RS01260-156 3.01e-115
VY93_RS01295-157 5.89e-108
VY93_RS01315-170 6.79e-93
LA081_RS01120-169 4.47e-96
LA081_RS01165-173 1.56e-90
LA081_RS01185-157 8.38e-122
LA081_RS01205-157 1.36e-120
LA081_RS01270-162 9.22e-127
LA081_RS01285-162 9.22e-127
LA081_RS01300-161 8.46e-122
LA081_RS01320-157 3.73e-84
LA081_RS01325-149 2.42e-74
LA081_RS01335-165 7.47e-90
LA081_RS01365-166 4.10e-92
LA081_RS01250-147 2.05e-124
K6987_RS02540-161 9.91e-114
K6987_RS02585-166 8.21e-93
K6987_RS02610-156 5.85e-122
K6987_RS02645-152 4.13e-85
K6987_RS02685-159 6.99e-114
K6987_RS02705-157 3.73e-84
K6987_RS02710-149 2.42e-74
K6987_RS02750-175 1.36e-99
K6989_RS02410-152 1.95e-107
K6989_RS02450-157 8.11e-85
K6989_RS02470-147 8.53e-114
K6989_RS02495-124 7.02e-4
K6989_RS02515-155 1.73e-96
K6989_RS02540-147 5.70e-105
K6989_RS02550-165 7.31e-91
K6989_RS02565-124 7.02e-4
K6989_RS02580-125 4.21e-58
K6989_RS02585-165 9.16e-86
MS53_RS03930-157 4.62e-112
MS53_RS01220-169 1.43e-95
MS53_RS01330-169 1.85e-92
DEH79_RS01155-155 4.38e-98
DEH79_RS01175-146 8.93e-119
DEH79_RS01195-155 4.38e-98
DEH79_RS01215-146 7.07e-117
DEH79_RS01230-142 1.31e-112
DEH79_RS01270-148 6.29e-110
DEH79_RS03715-136 3.30e-80
K6990_RS01695-131 4.64e-43
K6990_RS01735-162 3.45e-81
K6990_RS01750-187 7.31e-102
K6990_RS01760-135 4.52e-95
K6990_RS01775-171 7.18e-56
K6986_RS00625-152 1.95e-107
K6986_RS00670-157 8.11e-85
K6986_RS00710-165 7.31e-91
K6986_RS00720-159 4.37e-84
KXD88_RS01065-160 1.32e-88
KXD88_RS01125-148 1.90e-114
KXD88_RS01145-148 1.25e-113
KXD88_RS01155-148 3.24e-116
KXD88_RS01190-147 2.05e-124
KXD88_RS01205-153 5.73e-119
KXD88_RS01220-152 3.41e-114
KXD88_RS01240-157 3.73e-84
KXD88_RS01245-149 2.42e-74
KXD88_RS01260-156 6.44e-84
KXD88_RS01290-166 4.10e-92
K6988_RS00090-131 4.64e-43
K6988_RS00130-162 3.45e-81
K6988_RS00145-187 7.31e-102
K6988_RS00155-135 4.52e-95
K6988_RS00170-171 7.18e-56
EJ916_RS00965-166 4.10e-92
EJ916_RS01000-156 6.44e-84

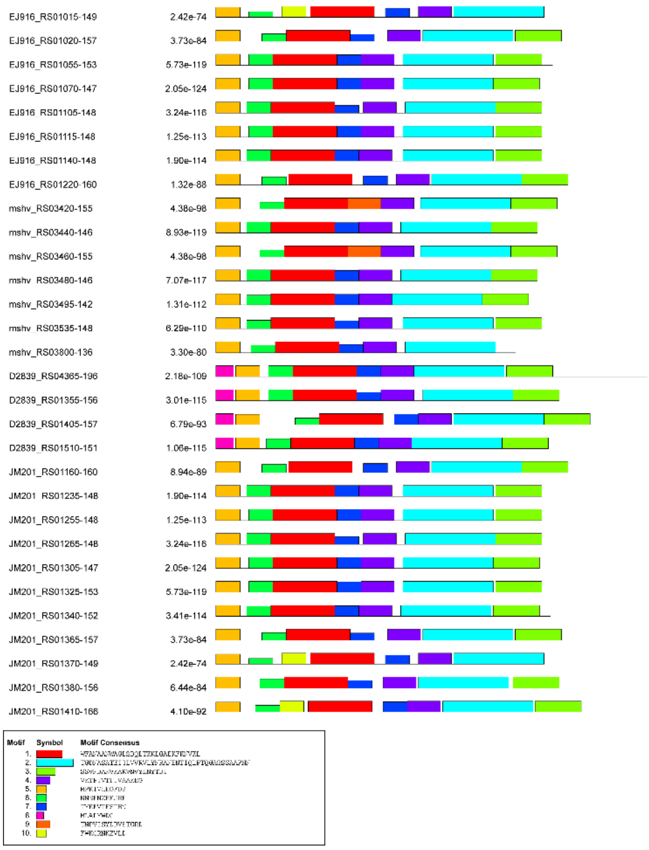

Figure S2. Short chain discovered motif and motif locations.
