# Supplementary material for: Residues Ser83, Arg85, Tyr88, Asn124, and Lys192 of C-Terminal Lipid-Associated Membrane Hemagglutinin Affect Mycoplasma synoviae Agglutination of Erythrocytes
Source: Microorganisms. 2026 Sep 12;14(9):2031. doi: 10.3390/microorganisms14092031 (PMC13609628; doi:10.3390/microorganisms14092031)
Supplement: Supplementary file 1 [file microorganisms-14-02031-s001.zip › Supplementary Data S4-Molecular docking analysis of interacting residues and hydrogen bonds.pdf]

Statistical Analysis of Docking Results between LAM HA and Representative Host Protein Interactors

1 Results and Analysis of LAM HA Connecting with SDCBP

Table S2. Cluster results of LAM HA and SDCBP docking.

Cluster	Members	Representative	Weighted Score
0	84	Center	-1081.0
		Lowest Energy	-1081.0
1	59	Center	-1019.1
		Lowest Energy	-1132.6
2	46	Center	-990.9
		Lowest Energy	-1209.8
3	42	Center	-1073.0
		Lowest Energy	-1226.2
4	41	Center	-1254.5
		Lowest Energy	-1292.9
5	36	Center	-1104.2
		Lowest Energy	-1207.5
6	33	Center	-993.9
		Lowest Energy	-1151.0
7	28	Center	-991.3
		Lowest Energy	-1135.9
8	26	Center	-1080.2
		Lowest Energy	-1211.0
9	25	Center	-1082.8
		Lowest Energy	-1138.8
10	24	Center	-1252.1

Cluster	Members	Representative	Weighted Score
		Lowest Energy	-1252.1

Table S3. Statistics of amino acid residues involved in hydrogen bond formation between LAM HA and SDCBP proteins.

LAM HA amino acid residues involved in the formation of hydrogen bonds	SDCBP amino acid residues and bond lengths that form hydrogen bonds with LAM HA
Pro191	Ser36 (2.6)、Ser37 (2.3)
Val86	Tyr46 (2.5)
Arg85	Tyr46 (2.1)
Thr89	Arg48 (2.3)
Asn2	Tyr50 (2.0)
Asn15	Gly44 (2.2)、Gly45 (2.6)
Lys18	Asp42 (2.0, 1.7)
Gln22	Gly44 (2.4)
Arg194	Ser36 (1.8, 2.1)、Leu32 (1.8, 1.9)、Ser33 (2.4)、Glu34 (1.8)

2 Results and Analysis of LAM HA Connecting with UBE2V2

Table S4. Cluster results of LAM HA docking with UBE2V2.

Cluster	Members	Representative	Weighted Score
0	185	Center	-611.4
		Lowest Energy	-694.8
1	63	Center	-770.7
		Lowest Energy	-863.3
2	60	Center	-694.7
		Lowest Energy	-710.4
3	55	Center	-637.3

Cluster	Members	Representative	Weighted Score
		Lowest Energy	-793.8
4	50	Center	-607.3
		Lowest Energy	-666.5
5	46	Center	-714.1
		Lowest Energy	-722.1
6	45	Center	-598.5
		Lowest Energy	-672.6
7	43	Center	-708.1
		Lowest Energy	-737.6
8	43	Center	-604.1
		Lowest Energy	-655.1
9	41	Center	-640.9
		Lowest Energy	-696.3
10	39	Center	-781.4
		Lowest Energy	-781.4

Table S5. Amino acid residue statistical table of LAM HA and UBE2V2 involved in hydrogen bond formation.

LAM HA amino acid residues involved in the formation of hydrogen bonds	UBE2V2 amino acid residues and bond lengths that form hydrogen bonds with LAM HA
Trp9	Thr5 (2.0)
Val86	Ser4 (2.0, 1.9)
Thr89	Val8 (2.5)
Thr92	Ser111 (2.1, 1.9)
Ala91	Asn110 (1.9)

3 Results and Analysis of LAM HA Connecting with HMBS

Table S6. Cluster results of LAM HA docking with HMBS.

Cluster	Members	Representative	Weighted Score

Cluster	Members	Representative	Weighted Score
0	105	Center	-592.4
		Lowest Energy	-724.9
1	88	Center	-577.2
		Lowest Energy	-691.5
2	71	Center	-673.4
		Lowest Energy	-750.5
3	59	Center	-586.3
		Lowest Energy	-650.1
4	42	Center	-653.6
		Lowest Energy	-670.8
5	42	Center	-623.7
		Lowest Energy	-687.2
6	39	Center	-761.3
		Lowest Energy	-761.3
7	37	Center	-691.5
		Lowest Energy	-691.5
8	35	Center	-706.8
		Lowest Energy	-707.9
9	34	Center	-596.1
		Lowest Energy	-653.0

Table S7. Statistical table of amino acid residues involving LAM HA and HMBS in hydrogen bond formation.

LAM HA amino acid residues involved in the formation of hydrogen bonds	HMBS amino acid residues and bond lengths that form hydrogen bonds with LAM HA
Arg171	Glu37 (1.8, 2.2, 1.8)
Arg270	Asp58 (2.0, 1.8)
Glu276	Arg170 (2.4, 1.8)、 Ser143 (2.1)

Glu277	Lys173 (1.8)、 Arg164 (1.8)
Gln278	Lys67 (1.7) 、Glu70 (2.0)
Ser273	Gln26 (1.9)
Ile272	Arg24 (2.4)

4 Results and Analysis of LAM HA Connecting with MAP2K1

Table S8. Cluster results of LAM HA docking with MAP2K1.

Cluster	Members	Representative	Weighted Score
0	118	Center	-696.4
		Lowest Energy	-863.8
1	85	Center	-728.5
		Lowest Energy	-782.0
2	53	Center	-828.9
		Lowest Energy	-838.9
3	45	Center	-724.4
		Lowest Energy	-854.1
4	37	Center	-774.5
		Lowest Energy	-803.0
5	34	Center	-864.6
		Lowest Energy	-864.6
6	32	Center	-835.5
		Lowest Energy	-835.5
7	31	Center	-743.0
		Lowest Energy	-775.6
8	29	Center	-773.0
		Lowest Energy	-773.0
9	25	Center	-787.3
		Lowest Energy	-787.3

Table S9. Statistical table of amino acid residues involved in hydrogen bond formation.

LAM HA amino acid residues involved in the formation of hydrogen bonds	MAP2K1 amino acid residues and bond lengths that form hydrogen bonds with LAM HA
Arg194	Gln230 (1.8)
Thr94	Leu229 (1.9)
Lys188	D311 (1.7, 1.8)、 Glu308 (1.8)
Gln22	Pro303 (2.4)
Glu17	Arg301 (2.1, 1,8)
Asn15	Thr220 (1.9)
Gln8	Gly74 (1.9)
Ala91	Ser222 (1.8)
Asn2	Asn215 (2.0)，Glu96 (2.0, 2.0)
Lys18	Pro302 (1.9)，Arg301 (1.8)

5 Results and Analysis of LAM HA Connecting with ARHGAP12

Table S10. Cluster results of LAM HA docking with ARHGAP121.

Cluster	Members	Representative	Weighted Score
0	48	Center	-1244.7
		Lowest Energy	-1599.9
1	44	Center	-1405.5
		Lowest Energy	-1474.9
2	40	Center	-1331.4
		Lowest Energy	-1414.6
3	34	Center	-1305.9
		Lowest Energy	-1345.5
4	32	Center	-1318.7
		Lowest Energy	-1374.2
5	27	Center	-1267.9
		Lowest Energy	-1534.5
6	26	Center	-1578.3

Cluster	Members	Representative	Weighted Score
		Lowest Energy	-1578.3
7	25	Center	-1430.6
		Lowest Energy	-1439.3
8	21	Center	-1275.4
		Lowest Energy	-1329.0
9	20	Center	-1245.1
		Lowest Energy	-1407.2

Table S11. Statistical table of amino acid residues involved in the formation of hydrogen bonds between LAM HA protein and ARHGAP12 protein.

LAM HA amino acid residues involved in the formation of hydrogen bonds	ARHGAP12 amino acid residues and bond lengths that form hydrogen bonds with LAM HA
Ser241	Ser187 (2.1)
Tyr244	Lys185 (1.7)
Arg262	Thr186 (1.9)、Ser187 (1.9,1.8)
Asn128	Thr186 (1.9,1.8)
Arg85	Ile183 (1.9)、Glu182 (2.0,1.8)
Ala121	Leu181 (1.8)
Lys122	Glu179 (1.8)
Asn124	Glu182 (2.1)、Glu179 (2.1)
Leu256	Glu179 (2.4)
Lys254	His174 (1.7)、Phe175 (2.1)
Thr266	Phe188 (1.8)、His190 (2.1,2.2)
Pro218	Gly173 (2.6)
Arg217	Ser171 (1.8)
Gln211	Ser171 (2.2)
Asn230	Thr169 (2.1)
Asn229	Arg168 (2.0,1.8)
Arg228	Arg168 (1.9)、Thr169 (2.7)
Asn227	Arg168 (2.6,1.8)、Gln166 (2.0)
Arg270	Asp195 (2.1,1.9)、Gln192 (1.9)
Ser273	Asp195 (2.5, 2.0, 2.0, 1.9)
Glu276	Glu199 (2.6, 2.1)、Gly198 (2.0)
Arg51	Glu179 (1.8)

Cluster	Members	Representative	Weighted Score

LAMHA & MRPL28

Cluster	Members	Representative	Weighted Score
0	184	Center	-804.4
		Lowest Energy	-987.3
1	98	Center	-771.4
		Lowest Energy	-962.2
2	65	Center	-784.3
		Lowest Energy	-853.2
3	53	Center	-828.7
		Lowest Energy	-930.2
4	40	Center	-764.2
		Lowest Energy	-848.7
5	34	Center	-875.2
		Lowest Energy	-1002.8
6	34	Center	-833.3
		Lowest Energy	-1029.0
7	30	Center	-992.2
		Lowest Energy	-998.7
8	25	Center	-762.2
		Lowest Energy	-819.6
9	24	Center	-938.1
		Lowest Energy	-996.5
10	23	Center	-926.8
		Lowest Energy	-926.8

LAMHA & CHAF1A

Cluster	Members	Representative	Weighted Score
0	57	Center	-1361.8
		Lowest Energy	-1478.6
1	21	Center	-1217.4
		Lowest Energy	-1351.0
2	21	Center	-1377.0
		Lowest Energy	-1377.0
3	20	Center	-1228.1
		Lowest Energy	-1297.0
4	20	Center	-1156.1
		Lowest Energy	-1244.2
5	18	Center	-1632.5
		Lowest Energy	-1829.6
6	18	Center	-1375.2
		Lowest Energy	-1436.2
7	17	Center	-1309.2
		Lowest Energy	-1446.7
8	15	Center	-1288.8
		Lowest Energy	-1288.8
9	14	Center	-1259.7
		Lowest Energy	-1356.5
10	14	Center	-1354.9
		Lowest Energy	-1402.8

LAMHA & ARFGAP2

Cluster	Members	Representative	Weighted Score
0	38	Center	-1482.7
		Lowest Energy	-1482.7
1	27	Center	-1304.1
		Lowest Energy	-1509.1
2	20	Center	-1418.3
		Lowest Energy	-1577.4
3	19	Center	-1436.8
		Lowest Energy	-1625.7
4	17	Center	-1260.7
		Lowest Energy	-1369.7
5	17	Center	-1459.7
		Lowest Energy	-1459.7
6	17	Center	-1364.9
		Lowest Energy	-1446.3
7	16	Center	-1301.9
		Lowest Energy	-1474.1
8	16	Center	-1455.9
		Lowest Energy	-1501.6
9	15	Center	-1260.1
		Lowest Energy	-1407.6
10	14	Center	-1357.4
		Lowest Energy	-1368.4

LAMHA & ACADL

Cluster	Members	Representative	Weighted Score
0	85	Center	-942.0
		Lowest Energy	-968.7
1	72	Center	-969.7
		Lowest Energy	-1214.8
2	62	Center	-951.3
		Lowest Energy	-1098.7
3	61	Center	-997.3
		Lowest Energy	-997.3
4	43	Center	-836.3
		Lowest Energy	-980.7
5	32	Center	-821.0
		Lowest Energy	-1131.4
6	30	Center	-895.3
		Lowest Energy	-1051.6
7	28	Center	-866.9
		Lowest Energy	-906.9
8	27	Center	-851.2
		Lowest Energy	-934.3
9	26	Center	-922.0
		Lowest Energy	-955.4
10	23	Center	-833.9
		Lowest Energy	-987.0

LAMHA & ERGIC2

Cluster	Members	Representative	Weighted Score
0	43	Center	-1219.9
		Lowest Energy	-1219.9
1	34	Center	-1110.4
		Lowest Energy	-1236.0
2	32	Center	-1175.3
		Lowest Energy	-1176.3
3	27	Center	-1070.1
		Lowest Energy	-1296.3
4	26	Center	-1103.9
		Lowest Energy	-1382.3
5	25	Center	-1163.2
		Lowest Energy	-1180.4
6	24	Center	-1196.1
		Lowest Energy	-1196.1
7	23	Center	-1208.8
		Lowest Energy	-1277.1
8	23	Center	-1207.7
		Lowest Energy	-1207.7
9	22	Center	-1072.5
		Lowest Energy	-1193.4
10	20	Center	-1253.9
		Lowest Energy	-1253.9

LAMHA & SPTSSA

Cluster	Members	Representative	Weighted Score
0	157	Center	-1015.0
		Lowest Energy	-1265.4
1	86	Center	-1017.0
		Lowest Energy	-1171.0
2	71	Center	-1031.7
		Lowest Energy	-1265.9
3	60	Center	-1123.5
		Lowest Energy	-1150.1
4	58	Center	-996.4
		Lowest Energy	-1214.7
5	54	Center	-1107.7
		Lowest Energy	-1302.9
6	45	Center	-1186.5
		Lowest Energy	-1186.5
7	40	Center	-1016.1
		Lowest Energy	-1116.3
8	28	Center	-1028.3
		Lowest Energy	-1107.5
9	27	Center	-1061.3
		Lowest Energy	-1186.6
10	26	Center	-1055.1
		Lowest Energy	-1145.3
