# Supplementary material for: Residues Ser83, Arg85, Tyr88, Asn124, and Lys192 of C-Terminal Lipid-Associated Membrane Hemagglutinin Affect Mycoplasma synoviae Agglutination of Erythrocytes
Source: Microorganisms. 2026 Sep 12;14(9):2031. doi: 10.3390/microorganisms14092031 (PMC13609628; doi:10.3390/microorganisms14092031)
Supplement: Supplementary file 1 [file microorganisms-14-02031-s001.zip › Supplementary Data S5-Multiple sequence alignment of LAM HA showing conserved residues S83, R85, Y88, N124, and K192.pdf]

Ser83, Arg85, Tyr88

```
LAMHA                 RDVRIPKVTFTVAAKDGYNLTEP--TGNTKQISLSIRVLYTSATST-QNALRYQGVAF--
LA081_RS01345-339     RDVRIPKVTFTVAAKDGYNLTEP--TGNTKQISLSIRVLYTSATST-QNALRYQGVAF--
KXD88_RS01270-330     RDVRIPKVTFTVAAKDGYNLTEP--TGNTKQISLSIRVLYTSATST-QNALRYQGVAF--
EJ916_RS00990-330     RDVRIPKVTFTVAAKDGYNLTEP--TGNTKQISLSIRVLYTSATST-QNALRYQGVAF--
JM201_RS01390-330     RDVRIPKVTFTVAAKDGYNLTEP--TGNTKQISLSIRVLYTSATST-QNALRYQGVAF--
DEH79_RS01285-367     RDVRIPKVTFTVAAKDGYNLTKP--TGETKQISLSIRVLYTSQNQT-ENALRYQGVSF--
mshv_RS03550-367      RDVRIPKVTFTVAAKDGYNLTKP--TGETKQISLSIRVLYTSQNQT-ENALRYQGVSF--
DEH79_RS01335-366     RDVRIPKVTFTVTAKDGYNLIAP--EGDAKQISLSIRVLYTSQNQT-ENALRYQGVSF--
D2839_RS01480-337     NTWKNPKVTFNIQAKPGYQLTEP--TTEESQISLSIRVLYSSQESN-QNLLTIQGASP--
K6987_RS02560-359     YTWKNPKVTFNIQAKPGYQLTEP--TTNPKQISLTIRVLYSSQESN-QNLLTIKGASP--
K6986_RS00645-350     YTWKNPKVTFNIQAKPGYQLTEP--TTNPKQISLTIRVLYSSQESN-QNLLTIKGASP--
DEH79_RS01255-320     RDVRIPKVTFTVTAKNGYNLTAP--TGNTKQISLLIRVLYTSASST-ENALRYQGVSF--
mshv_RS03520-320      RDVRIPKVTFTVTAKNGYNLTAP--TGNTKQISLLIRVLYTSASST-ENALRYQGVSF--
K6986_RS00585-334     RDVRIPKVTFTVAAKDGYNLTVP--TGNTKQISLSIRVLYTSENST-QNALRYQGASS--
MS53_RS01225-364      RDVRIPKVTFTVTAKNGYNLTAP--TGNTKQISLSIRVLYTSASST-ENALRYQGVSF--
LA081_RS01125-343     RDVRIPKVTFTVTVKNGYNLIAP--EGDAKQISLSIRVLYTSASST-ENALRYQGVSF--
KXD88_RS01070-334     RDVRIPKVTFTVTVKNGYNLIAP--EGDAKQISLSIRVLYTSASST-ENALRYQGVSF--
EJ916_RS01215-334     RDVRIPKVTFTVTVKNGYNLIAP--EGDAKQISLSIRVLYTSASST-ENALRYQGVSF--
```

Asn124

```
LAMHA                 SGAPSGSLVD-DANVKAKVNVYMNYTGPAIVLDQAVPAVGTATNTSLNGTSNVTGDF-NN
LA081_RS01345-339     SGAPSGSLVD-DANVKAKVNVYMNYTGPAIVLDQAVPAVGTATNTSLNGTSNVTGDF-NN
KXD88_RS01270-330     SGAPSGSLVD-DANVKAKVNVYMNYTGPAIVLDQAVPAVGTATNTSLNGTSNVTGDF-NN
EJ916_RS00990-330     SGAPSGSLVD-DANVKAKVNVYMNYTGPAIVLDQAVPAVGTATNTSLNGTSNVTGDF-NN
JM201_RS01390-330     SGAPSGSLVD-DANVKAKVNVYMNYTGPAIVLDQAVPAVGTATNTSLNGTSNVTGDF-NN
DEH79_RS01285-367     SAAPNGANSVNHAQTIKDVNVYLNYTGPSIVLDEALPTVGTTPNTSINGTSNVEGTF-ND
mshv_RS03550-367      SAAPNGANSVNHAQTIKDVNVYLNYTGPSIVLDEALPTVGTTPNTSINGTSNVEGTF-ND
DEH79_RS01335-366     SAAPSGSS-VDDANVKAKVNVYLNYTGPSIVLDQNLPTVGTANNTSLNGTSNVTGDF-NT
D2839_RS01480-337     TAAPNNTRNANDETTKKTVNVYLNYTGPSIVLDQAVPAVGTANNTSLNGTSNVTGDF-NT
K6987_RS02560-359     SEAPSNSSVDDA-NVKAKVNVYMNYTGPDIVLDQAVPAVGTANNTSLNGTSNVTGEF-NT
K6986_RS00645-350     SEAPSNSSVDDA-NVKAKVNVYMNYTGPDIVLDQAVPAVGTANNTSLNGTSNVTGEF-NT
DEH79_RS01255-320     SATPKDTSVENPATAIKNVNVYLNYTGPSIVLDQAVPAVGTANNTSLNGTSNVTGAF-NN
mshv_RS03520-320      SATPKDTSVENPATAIKNVNVYLNYTGPSIVLDQAVPAVGTANNTSLNGTSNVTGAF-NN
K6986_RS00585-334     SAATSNSSVDDA-NVKAKVNVYLNYTGPEIMLNAELPQVGNKNNTSINGTSNVDGDF-NT
MS53_RS01225-364      SATPKDASVENPATAIKNVNVYLNYTGPSIVLDQAVPAVGTANNTSLNGTSNVDGDF-NT
LA081_RS01125-343     SATPKDASVENPATAIKNVNVYLNYTGPSIVLDQAVPAVGTANNTSINGTSNVEGTF-NT
KXD88_RS01070-334     SATPKDASVENPATAIKNVNVYLNYTGPSIVLDQAVPAVGTANNTSINGTSNVEGTF-NT
EJ916_RS01215-334     SATPKDASVENPATAIKNVNVYLNYTGPSIVLDQAVPAVGTANNTSINGTSNVEGTF-NT
```

```
                                                                    Lys192
LAMHA                  KFNKLLIR-----YKAETSLFQTIINYVNKFDPKFRAQFVTN-SINGVT--ITK------
LA081_RS01345-339      KFNKLLIR-----YKAETSLFQTIINYVNKFDPKFRAQFVTN-SINGVT--ITK------
KXD88_RS01270-330      KFNKLLIR-----YKAETSLFQTIINYVNKFDPKFRAQFVTN-SINGVT--ITK------
EJ916_RS00990-330      KFNKLLIR-----YKAETSLFQTIINYVNKFDPKFRAQFVTN-SINGVT--ITK------
JM201_RS01390-330      KFNKLLIR-----YKAETSLFQTIINYVNKFDPKFRAQFVTN-SINGVT--ITK------
DEH79_RS01285-367      KFKKLLVN-VVRGGHAESSLFQAVINYVNKFDPKFRAVFVTN-SRDGVT--ITK------
mshv_RS03550-367       KFKKLLVN-VVRGGHAESSLFQAVINYVNKFDPKFRAVFVTN-SRDGVT--ITK------
DEH79_RS01335-366      KFKKLLVR-VLNGGHAESSLFQAVINYVNKFDPKFRAVFVTN-SRDGVT--ITK------
D2839_RS01480-337      KFKKLLVS-VLRRGHAESSLFQAIINYVNKFDPKFRAQFVTN-SIDGVT--ITK------
K6987_RS02560-359      KFKKLLVN-VVSRGHAESSLFQAVINYVNKFDPKFRAQFVTN-SINGVT--ITK------
K6986_RS00645-350      KFKKLLVN-VVSRGHAESSLFQAVINYVNKFDPKFRAQFVTN-SINGVT--ITK------
DEH79_RS01255-320      AFRGNPSRGLLFTNRYPNPLLQSVINYVNKFDPKFPAVFVTN-ATNGVT--ITK------
mshv_RS03520-320       AFRGNPSRGLLFTNRYPNPLLQSVINYVNKFDPKFPAVFVTN-ATNGVT--ITK------
K6986_RS00585-334      KFKKLLVN-VVSRGHAESSLFQAVINYVNKFDPKFRAQFVTN-SINGVT--ITK------
MS53_RS01225-364       KFKKLLVR-VLNGGHAESSLFQAVINYVNKFDPKFRAQFVTN-SINGVT--ITK------
LA081_RS01125-343      KFKKLLVN-VVRGGHAESSLFQAVINYVNKFDPKFRAQFVTN-SINGVT--ITK------
KXD88_RS01070-334      KFKKLLVN-VVRGGHAESSLFQAVINYVNKFDPKFRAQFVTN-SINGVT--ITK------
EJ916_RS01215-334      KFKKLLVN-VVRGGHAESSLFQAVINYVNKFDPKFRAQFVTN-SINGVT--ITK------
```

Figure S3. Conservation analysis of Ser83, Arg85, Tyr88, Asn124, and Lys192.
