# Supplementary material for: Residues Ser83, Arg85, Tyr88, Asn124, and Lys192 of C-Terminal Lipid-Associated Membrane Hemagglutinin Affect Mycoplasma synoviae Agglutination of Erythrocytes
Source: Microorganisms. 2026 Sep 12;14(9):2031. doi: 10.3390/microorganisms14092031 (PMC13609628; doi:10.3390/microorganisms14092031)
Supplement: Supplementary file 1 [file microorganisms-14-02031-s001.zip › Supplementary Data S6-Statistical data for molecular dynamics simulations.pdf]

Table S12. Analysis parameters.

| Parameter | Description |
| --- | --- |
| Analysis metrics | RMSD, RMSF, Rg, SASA, and hydrogen bonds (HB) |
| pH conditions | pH 7.0–7.5, pH 6.0–6.5, and pH 5.0–5.5 |
| Statistical analysis | Mean ± standard deviation (SD) calculated from the equilibrated last 50% of simulation trajectories |
| Units | RMSD, RMSF and Rg in Å; SASA in nm²; HB as count |

Table S13. Summary of statistical results for each system.

| System | pH | RMSD (Å) | RMSF (Å) | Rg (Å) | SASA (nm²) | HB (number) |
| --- | --- | --- | --- | --- | --- | --- |
| LAM HA | 7.0–7.5 | 11.27 ± 0.40 | 2.72 ± 1.86 | 38.53 ± 0.96 | 134.70 ± 8.55 | 134.70 ± 8.55 |
| LAM HA | 6.0–6.5 | 12.14 ± 0.21 | 2.83 ± 1.88 | 40.48 ± 1.31 | 125.63 ± 7.76 | 125.63 ± 7.76 |
| LAM HA | 5.0–5.5 | 13.69 ± 0.19 | 3.10 ± 1.98 | 39.95 ± 1.25 | 127.07 ± 7.33 | 127.07 ± 7.33 |
| Mut_LAM HA | 7.0–7.5 | 13.18 ± 0.27 | 3.22 ± 1.98 | 43.68 ± 1.68 | 113.80 ± 7.41 | 113.80 ± 7.41 |
| Mut_LAM HA | 6.0–6.5 | 11.17 ± 1.33 | 4.18 ± 2.65 | 54.16 ± 4.47 | 113.50 ± 7.59 | 113.50 ± 7.59 |
| Mut_LAM HA | 5.0–5.5 | 12.50 ± 1.21 | 4.42 ± 2.71 | 42.39 ± 4.20 | 113.59 ± 7.77 | 113.59 ± 7.77 |
